# Mathematical modeling of pneumococcal transmission dynamics in response to PCV13 infant vaccination in Germany predicts increasing IPD burden due to serotypes included in next-generation PCVs

**DOI:** 10.1371/journal.pone.0281261

**Published:** 2023-02-15

**Authors:** Matthias Horn, Christian Theilacker, Ralf Sprenger, Christof von Eiff, Ernestine Mahar, Julia Schiffner-Rohe, Mathias W. Pletz, Mark van der Linden, Markus Scholz

**Affiliations:** 1 Institute for Medical Informatics, Statistics and Epidemiology, University of Leipzig, Leipzig, Germany; 2 Pfizer Pharma GmbH, Berlin, Germany; 3 Institute for Infectious Diseases and Infection Control, Jena University Hospital, Jena, Germany; 4 Institute of Medical Microbiology, German National Reference Centre for Streptococci, University Hospital RWTH Aachen, Aachen, Germany; Health Directorate, LUXEMBOURG

## Abstract

**Introduction:**

Two next-generation pneumococcal conjugate vaccines (PCVs), a 15- and a 20-valent PCV (PCV15 and PCV20), have recently been licensed for use in adults, and PCV15 has also been licensed in children. We developed a dynamic transmission model specific for Germany, with the aim to predict carriage prevalence and invasive pneumococcal disease (IPD) burden for serotypes included in these vaccines.

**Methods:**

The model allows to follow serotype distributions longitudinally both in the absence and presence of PCV vaccinations. We considered eight age cohorts and seven serotype groups according to the composition of different pneumococcal vaccines. This comprises the additional serotypes contained in PCV15 and PCV20 but not in PCV13.

**Results:**

The model predicted that by continuing the current vaccine policy (standard vaccination with PCV13 in children and with PPSV23 in adults) until 2031, IPD case counts due to any serotype in children <2 years of age will remain unchanged. There will be a continuous decrease of IPD cases in adults aged 16-59y, but a 20% increase in adults ≥60y. Furthermore, there will be a steady decrease of the proportion of carriage and IPD due to serotypes included in PCV7 and PCV13 over the model horizon and a steady rise of non-PCV13 serotypes in carriage and IPD. The highest increase for both pneumococcal carriage and absolute IPD case counts was predicted for serotypes 22F and 33F (included in both PCV15 and PCV20) and serotypes 8, 10A, 11A, 12F, and 15B (included in PCV20 only), particularly in older adults. Between 2022 and 2031, serotypes included in PCV20 only are expected to cause 19.7–25.3% of IPD cases in adults ≥60y.

**Conclusions:**

We conclude that introduction of next-generation PCVs for adults may prevent a substantial and increasing proportion of adult IPDs, with PCV20 having the potential to provide the broadest protection against pneumococcal disease.

## Introduction

*Streptococcus pneumoniae* is among the most frequent causes of vaccine-preventable morbidity and mortality both in children and adults globally [1]. It causes invasive infections, such as bacteremic pneumonia, meningitis, or primary sepsis, and non-invasive infections, such as non-bacteremic pneumonia, otitis media or sinusitis [2]. In adults, non-bacteremic pneumonia is by far the most common clinical presentation of pneumococcal disease, and more than 80% of pneumococcal pneumonia is non-bacteremic [3]. The pneumococcus primarily resides in the nasopharynx of young children, from which it transmits by respiratory droplets to other individuals [4]. Upper airway carriage of the pneumococcus is a prerequisite to cause disease. With a carriage prevalence between 20–60% among healthy children, this young population is considered the main reservoir for spreading infection to other children as well as to adults [4, 5]. The pneumococcal polysaccharide capsule is the most common virulence factor of *S*. *pneumoniae*, and with 100 known capsular serotypes it is also highly diverse [6]. Serotypes differ in propensity to cause disease and case fatality [7–9].

Currently, two pneumococcal vaccines are recommended for use in adults in Germany: a 23-valent pneumococcal polysaccharide vaccine (PPSV23; containing serotypes 1, 2, 3, 4, 5, 6B, 7F, 8, 9N, 9V, 10A, 11A, 12F, 14, 15B, 17F, 18C, 19A, 19F, 20, 22F, 23F, 33F) and a 13-valent pneumococcal conjugate vaccine (PCV13; containing serotypes 1, 3, 4, 5, 6A, 6B, 7F, 9V, 14, 18C, 19A, 19F, 23F) for immunocompromised individuals. Vaccines recommended for pneumococcal disease prevention in children are PCV13 and a 10-valent pneumococcal conjugate vaccine (PCV10; containing serotypes 1, 4, 5, 6B, 7F, 9V, 14, 18C, 19F, 23F). PPSV23 has been recommended for adults ≥60y in Germany since 1998 [10]. In children, PCV7 (containing serotypes 4, 6B, 9V, 14, 18C, 19F, 23F) was first universally recommended in 2006 and replaced by either PCV10 or PCV13 in 2009, with PCV13 being used almost exclusively in recent years [11]. Pneumococcal vaccination is also recommended for all individuals with underlying chronic conditions as well as for individuals with congenital or acquired immunodeficiency [12]. Vaccination rates for PCVs in children <2y are suboptimal with 90% and 75% for the primary and booster dose, respectively [13]. The uptake of pneumococcal vaccination among older adults has been low, with only 22.5% of adults aged 60-73y having received a dose of PPSV23 [14].

Similar to observations in other high-income countries, pediatric PCV vaccination in Germany led to substantial reductions of overall and vaccine-type IPD in vaccinated children [15, 16]. The impact of universal pediatric PCV13 vaccination also led to substantial changes in the IPD serotype distribution in this age group, with near eradication of IPD caused by PCV7 serotypes. Furthermore, substantial reductions of the six additional serotypes contained in PCV13 were observed. These large absolute reductions in IPD due to PCV13 serotypes in children have been accompanied by both relative and absolute increases in IPD due to serotypes not included in the PCV13 formulation [17, 18]. Due to the ability of PCVs to reduce nasopharyngeal carriage and transmission to others, the introduction of pediatric PCV vaccinations has also led to rapid reductions in IPD incidence rates (IRs) across all age groups [19]. However, in older adults, the herd protection effects for the additional six serotypes in PCV13 were not as pronounced as the initial effects seen after introduction of PCV7. The initial decline was followed by an increase in IPD IRs after 2015, reducing the net indirect benefits of PCV10/PCV13 programs [19]. The most prevalent serotypes among adults ≥60y with IPD in Germany in 2017/2018 were serotypes 3 (20.9%), 8 (9.8%), 22F (7.6%), 9N (7.0%), and 19A (7.0%) [18].

In contrast to IPD, data on serotype distributions of non-bacteremic community-acquired pneumonia (CAP) are sparse. In routine medical care, many cases of pneumococcal CAP are diagnosed using a urinary antigen test (BinaxNow *S*. *pneumoniae*®) [20], which does not discriminate between different serotypes. However, investigation of urine samples from a German cohort of patients with radiologically confirmed CAP (CAPNETZ) using a serotype-specific urinary antigen test, verified the trends observed for IPD [21–23]. Among adults ≥18y with CAP in Germany, serotypes 3, 8, 22F, and 11A were the most prevalent serotypes between 2013 and 2019 [21].

The observed changes in global serotype epidemiology since the introduction of universal PCV vaccination have led to the development of next-generation PCVs of higher serotype valency, including a 15-valent PCV (PCV15) and a 20-valent PCV (PCV20) [24, 25], both of which were recently licensed for adults ≥18y (PCV15: European Commission [EC] decision Oct 14, 2021; PCV20: EC decision Feb 14, 2022). PCV15 was also licensed for use in children (EC decision Sep 15, 2022). PCV15 contains all serotypes included in PCV13 plus serotypes 22F and 33F. PCV20 contains serotypes 8, 10A, 11A, 12F, and 15B in addition to those included in PCV15. Recommendations for these vaccines need to be informed by burden estimates from pneumococcal disease surveillance and model predictions on future disease epidemiology. However, for disease burden predictions, dynamic transmission models are needed. To better understand the complex interplay of different pneumococcal serotypes in different age groups in Germany, and to provide predictions on future serotype distributions, we developed a dynamic model of pneumococcal serotype distributions both in the absence and presence of PCV vaccinations use. This model is the first to study potential dynamics of the additional serotypes included in PCV15 and PCV20 but not in PCV13. Our approach is based on a model originally developed on behalf of the Robert Koch-Institute for a health economic evaluation of adult pneumococcal vaccinations in Germany, which was published as a final study report [26]. Similar models have been successfully applied elsewhere [27–29].

We used our model to predict longitudinal serotype dynamics spanning over 20 years and found that carriage of PCV13 serotypes continuously decreases in all age groups, although predictions for serotype 3 were less certain. In contrast, carriage of non-PCV13 serotypes is predicted to increase considerably. More precisely, we predict serotypes 22F and 33F as well as serotypes 8, 10A, 11A, 12F, and 15B to be associated with the highest increase over the next 10 years for both pneumococcal carriage and absolute IPD case counts, particularly in older adults. These serotypes are included in neither PCV7 nor PCV13, but in novel vaccines PCV15 and PCV20, respectively.

## Material and methods

### Modeling approach

To build the dynamic model, we adapted previous approaches [26, 29, 30]. Our model consists of an epidemiological submodel, describing transmission of pneumococcal serotypes, and a demographic submodel of population dynamics in Germany and its age structure. The basic epidemiological model first described undisturbed pneumococcal carriage dynamics, resulting in a long-term steady-state. Relatively constant IPD IRs in different age groups pre-PCV introduction supported our steady-state assumption [31]. By coupling the epidemiological model to a vaccination model, the steady-state was altered, resulting in a new steady-state of differing serotype distributions after a dynamic period.

We aggregated pneumococcal serotypes into seven groups (see Table 1). Due to cross-protection of antibodies raised against serotype 6A [32], we assumed PCV13 serotypes to additionally include serotype 6C as done by other models [33]. Serotypes within one group were assumed to behave equally with respect to transmission dynamics and PCV impact on carriage. Every individual was assumed to simultaneously carry either no serotype (non-carriers; denoted as *S*), serotypes of exactly one serotype group (single carriers; *G*1, …, *G*7) or serotypes of exactly two serotype groups (double carriers; *G*1*G*2, …, *G*6*G*7). Co-carriage of more than two serotype groups was excluded to reduce model complexity. Hence, 29 different states of carriage (including *S*) were described. Carriage of two or more serotypes belonging to the same group was considered as single carriage.

**Table 1:** Overview of the seven serotype groups defined in the model.

Serotype group	Identifier	Serotypes
*G*1	PCV7	4, 6B, 9V, 14, 18C, 19F, 23F
*G*2	PCV13 non-PCV7–3 +6C	1, 5, 6A, 6C, 7F, 19A
*G*3	Serotype 3 only	3
*G*4	PCV15 non-PCV13	22F, 33F
*G*5	PCV20 non-PCV15	8, 10A, 11A, 12F, 15B
*G*6	PPSV23 non-PCV20	2, 9N, 17F, 20
*G*7	Other serotypes	all other serotypes

Note the additive definition, i.e., every serotype is included in exactly one group only. Groups are based on the serotypes included in the vaccines of the same names, but not covered by other groups, with one notable exception: *G*2 additionally includes serotype 6C due to reported cross-protection from 6A.

The epidemiological model was derived from Susceptible/Infected/Recovered (SIR) principles but differs from the standard model in that “recovered” is associated with a loss of pneumococcal carriage but not with immunity (so-called Susceptible/Infected/Susceptible [SIS] model).

The propensity of a non-carrier to become a single carrier was described by serotype group-specific transmission risks λ. These risks were calculated from the number of carriers within the respective serotype group in the population at a certain time (sum of single and double carriers) and their contact frequencies β with non-carriers [34]. Transmission probabilities α (assumed to be constant over time) describe the probability of a serotype transmission per contact. These probabilities are potentially different for each of the seven serotype and eight age groups (see below).

Single carriers can acquire serotypes of a different serotype group. Due to competition of serotypes for colonization of the nasopharyngeal area, we assumed a reduction of the transmission risk by multiplying λ by a competition parameter *c* (fixed at 0.5, for sensitivity analysis ranging from 0 to 1 in steps of 0.1). Loss of carriage was described by the parameter *r*. This rate was assumed to be independent of serotype group and carriage state (single/double carrier), but as reported by Högberg et al. [35], it was assumed to be age-dependent.

**Fig 1:**
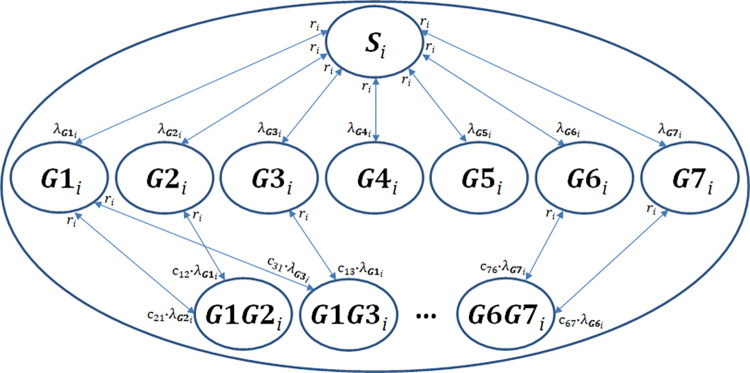
Scheme of the epidemiological model. We assumed acquisition and loss of carriage to be stepwise processes, i.e., non-carriers can become single carriers, who can become double carriers or non-carriers again; double carriers first become single carriers before becoming non-carriers again. For a detailed description see text. *S*: non-carriers; *G*1,…,*G*7: carriers of serotype group 1,…,7; *G*1*G*2,…,*G*6*G*7: double carriers (2 serotype groups); *i* = 1,…,400: age stratum; *λ*_**G1**_,…,*λ*_**G7**_: age-dependent transmission risks; *c*_12_,…,*c*_76_: competition parameters; *r*: age-dependent rates of loss of carriage.

We considered 400 age strata (subscript i) representing 3 months each, resulting in a total age range of [0; 100] years. Every 3 months the model population aged simultaneously by 3 months while newborns (assumed to be non-carriers) entered the model system. Except for 99.75-year-old subjects leaving the model system every 3 months, mortality was not considered. The model was later calibrated based on actual age distributions. Based on parsimony considerations, we summarized age strata to eight different groups representing different parameter settings (<2, 2–4, 5–15, 16–44, 45–59, 60–74, 75–84, ≥85y). For a simplified model scheme see Fig 1.

**Fig 2:**
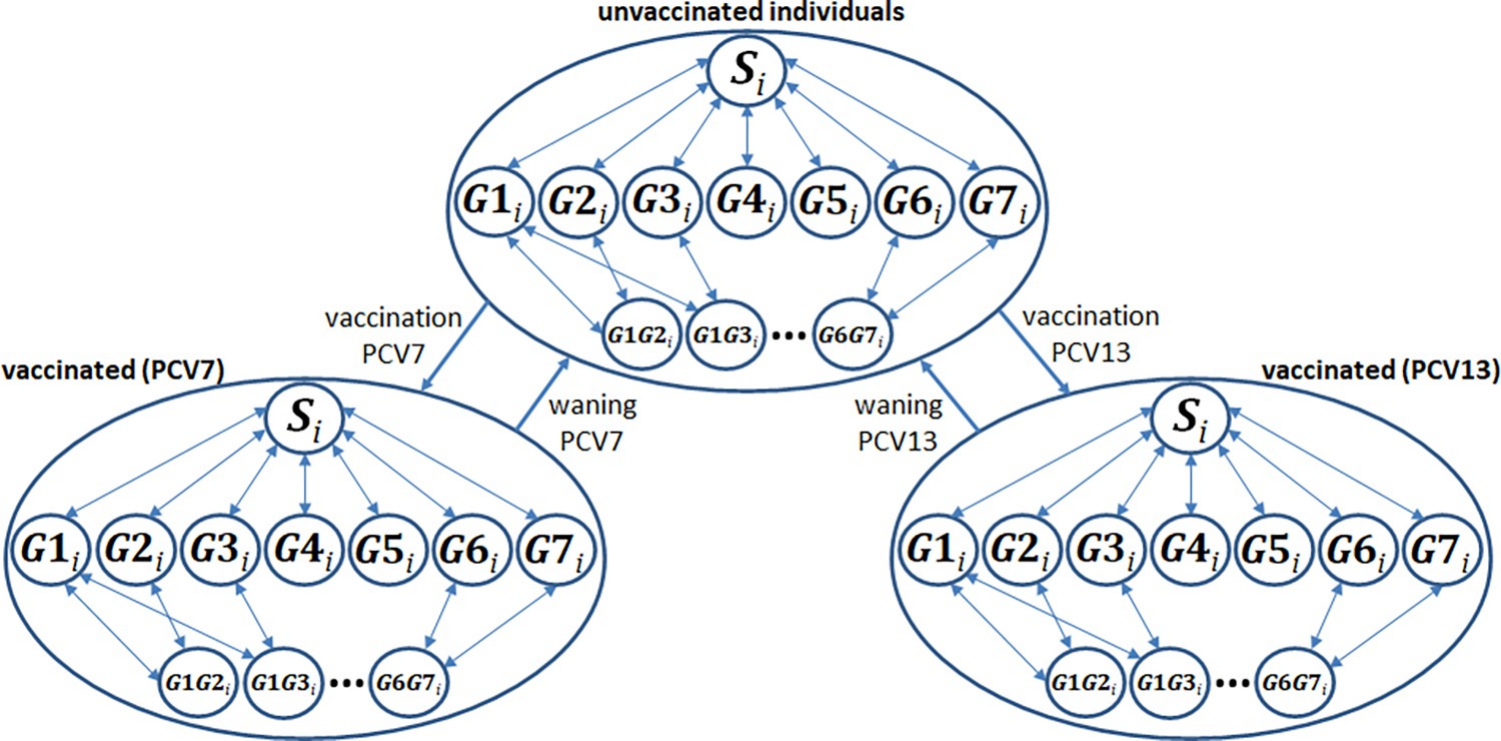
Scheme of the vaccination model. The model is an extension of the epidemiological model depicted in Fig 1. Three different states were distinguished: individuals can be unvaccinated or vaccinated with either PCV7 or PCV13. Transitions between states were described via vaccination and waning rates. For a detailed description see text. *S*: non-carriers; *G*1,…,*G*7: carriers of serotype group 1,…,7; *G*1*G*2,…,*G*6*G*7: double carriers (2 serotype groups); *I* = 1,…,400: age stratum.

The basic epidemiological model was amended by a vaccination model describing the impact of two different vaccines (PCV7 and PCV13) on serotype dynamics. For a representation of the vaccination model see Fig 2. In detail, we triplicated the basic model by introducing two vaccinated populations. For each unvaccinated state we added a PCV7 (denoted as ν1) and a PCV13 (ν2) vaccinated state. Transitions from an unvaccinated to a vaccinated state and vice versa were described by age-dependent vaccination (denoted as π) and waning rates (ω), respectively. We additionally introduced age-independent parameters (ν) describing the impact of a vaccine on carriage of a given serotype group. We distinguish between vaccine (serotypes included in *G*1-*G*3) and non-vaccine serotypes (all others). Impact on carriage must not be confused with the potentially differing clinical impact on e.g. IPD. Regarding pneumococcal transmission, no difference between vaccinated and unvaccinated carriers was assumed. For model equations see S1 File.

The full model (including vaccinations) consists of 34,800 ordinary differential equations (400 age strata, 87 state parameters). Equations were solved numerically by daily updates using C++ code embedded into the statistical programming environment R [36].

### Vaccination history

For model fitting, we reproduced the German PCV vaccination history except for the PCV10 vaccine, administered only in a small and decreasing percentage of children starting from 2009 [16], which was excluded for reasons of simplicity. Also excluded was PPSV23 vaccination, please refer to the discussion section for full rationale on this exclusion. We considered universal pediatric vaccination starting in 2005/06 (PCV7) and 2009/10 (PCV13), and PCV13 vaccination of older age groups (≥60y) was assumed to start in 2012/13. Due to lack of data, vaccinations of immunocompromised individuals were not described separately. Waning rates were assumed to be identical for all children age groups. Additionally, we assumed all vaccination parameters to be constant over time, except for PCV7 vaccination rates, which were set to zero upon PCV13 introduction. Constant vaccination parameters were also assumed for future vaccinations, i.e., PCV13 vaccinations were assumed to remain unchanged until 2031. We did not consider hypothetical vaccination effects of PCV15/PCV20 potentially introduced in the future.

### Carriage data

Due to a lack of suitable pneumococcal carriage studies regarding the population in Germany, carriage for each age and serotype group was estimated using German IPD IRs in combination with case-carrier ratios (CCR) reported for the respective serotype group [28]. We assumed the year 2005/06 to be representative for the pre-PCV vaccination steady-state situation of serotype distributions. After 2006, serotype distributions were assumed to be affected by PCV vaccinations. For carriage estimation, we used two data sources of IPD incidences: (i) IPD IRs taken from the literature (“adjusted IRs”), which were estimated using the capture-recapture method [17, 31, 37], and (ii) raw data of potentially underreported IPD case counts (“reported IRs”) obtained courtesy of Mark van der Linden from the German National Reference Center for Streptococci. This database comprises the years 1992 to 2022 however only data up to 2019/20 was used in the model. For each epidemiological year (from July 1^st^ to June 30^th^ of the following year) we applied the following algorithm:

From the raw data we calculated reported IPD IRs separately for children (0-15y) and adults (≥16y). For these calculations we used age distributions reported by the German Federal Office of Statistics. For forecasting of future age distributions, we applied a model that assumes moderate increases in life expectancy and migration net-balance, while birth rates are assumed to remain unchanged. Comparing these reported IRs to the adjusted IPD IRs of children and adults from the literature, we calculated year-specific underreporting factors (UFs) for both children and adults as well as corresponding 95% confidence intervals (CIs). Using the raw data again, we calculated reported IPD IRs for each serotype and age group (56 values total per year). These IRs were multiplied by the UFs to obtain adjusted IRs (and 95% CIs) for each subgroup.

Pneumococcal carriage was calculated from the age and serotype group-specific IPD IRs via IPD CCRs for serotype groups *G*1 (PCV7), *G*2 (PCV13 non-PCV7–3 +6C), and *G*7 (other serotypes) [28]. CCRs of serotype group *G*3 (serotype 3) were assumed to be identical to *G*2 CCRs. For groups *G*4 (PCV15 non-PCV13), *G*5 (PCV20 non-PCV15), and *G*6 (PPSV23 non-PCV20) the assumption was made that those groups behave identical to group *G*7. We assumed CCRs to be constant over time. For a detailed explanation see S1 File. For comparison, we also provided carriage prevalence data from other European countries before PCV introduction (S6 Table in S1 File). Conversely, IPD IRs for each subgroup were calculated from pneumococcal carriage prevalence at a given time using CCRs. For annual IPD case counts, the reported or projected age structure in the German population, as described in the demographic submodel, was applied to the previously calculated IPD IRs.

An ethical approval was not required since the study was performed with *Streptococcus pneumoniae* isolates that resulted from routine microbiological diagnostic procedures as requested by the treating physician. No additional biological specimens were taken for the purpose of this study. Specimens were anonymized and only data on year and month of birth, sex, vaccination status, type of specimen, and hospital/laboratory where the case was diagnosed were registered.

### Model parameterization

Although many parameter values were taken from literature data (rates of carriage loss *r*, contact frequencies β; see S1 File), several parameters of the epidemiological model were unknown or only known within ranges. Therefore, it was necessary to estimate these parameters by fitting the model to pneumococcal carriage data (see previous section). We first estimated the 56 transmission probabilities α using data of the steady-state of the basic epidemiological model. To approximate the steady-state, we initialized the model with arbitrary starting values. Then, the model system was simulated for 50 years. To fit the more complex vaccination model, we kept the previously estimated parameters constant, i.e., we only estimated vaccine-related parameters such as vaccination and waning rates as well as impact parameters within sensible ranges. We excluded age group 2-4y from fitting the vaccination model, but not from fitting the basic epidemiological model. This was decided due to numerical artifacts in calculated carrier prevalences caused by very low reported IPD case numbers in this age group after PCV introduction. For simultaneous estimation of many parameters, we used the gradient-based Broyden-Fletcher-Goldfarb-Shanno (BFGS) algorithm (function “optim” in the R package “stats”) [36]. According to downscaled pilot calculations, this was the most performant optimization algorithm pre-implemented in R. Application of other algorithms (e.g., Levenberg-Marquardt) resulted in similar parameter values, but at a slower convergence rate.

To analyze the sensitivity of the model predictions to parameter changes (e.g., different vaccination rates), we calculated 95% prediction intervals based on 1,000 simulations with varying vaccination parameters. As highly sensitive microarray-based serotyping methods are rarely applied, only the most dominant serotype is usually reported for each patient. In contrast, our model additionally considers double carriage. To compare model and data, we redistributed double carriers uniformly among their corresponding single carrier compartments. For instance, 50% of *G*1*G*3 double carriers were added to *G*1 and 50% to *G*3 single-carrier states. This redistribution was performed for fitting (and visualization) purposes only.

## Results

### Unvaccinated steady-state

**Fig 3:**
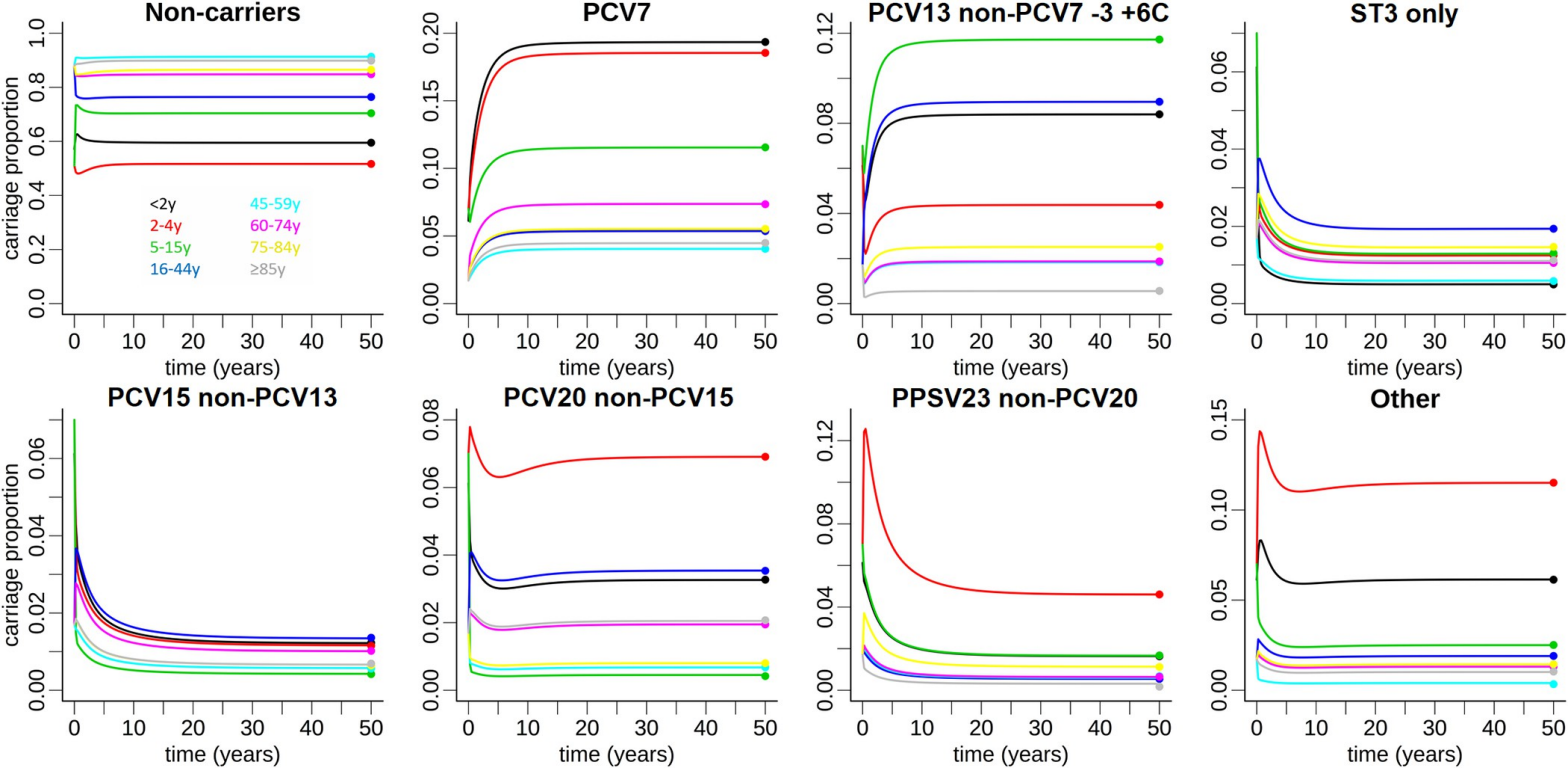
Results of the fitting process of the basic epidemiological model (i.e., without vaccinations). Initializing the model with arbitrary starting values, the model system was simulated for 50 years until a steady-state in all compartments was reached. The rightmost timepoint corresponds to the epidemiological year 2005/06 (pre-PCV vaccinations), where the agreement between model and data points (pneumococcal carriage proportions in Germany) was optimized. While the results are depicted separately for each serotype group (figure headings), age groups are visualized by colors: black (<2y), red (2-4y), green (5-15y), blue (16-44y), cyan (45-59y), purple (60-74y), yellow (75-84y), gray (≥85y). Model oscillations due to the assumption of simultaneous aging were smoothed out.

Transmission probabilities were determined such that an optimal agreement between data and simulated steady-state of the model (Fig 1) could be observed in all compartments and subgroups (Fig 3). In the steady-state, we predict approximately 0.14–5.5% of persons (including non-carriers) to be double carriers, with the highest proportions observed in the 2-4y, followed by the <2y age group. Estimated transmission probabilities are summarized in S5 Table in S1 File.

### Vaccination model

For the vaccination model (Fig 2), all parameters of the baseline model were kept constant. We only estimated vaccination-related parameters (vaccination and waning rates, impact on carriage) using PCV era (post-2005) data of pneumococcal carriage in Germany, which are given in Table 2.

**Table 2:** Estimated parameters of the vaccination model (with 95% CIs) in percent.

	PCV7 vaccine (%)	PCV13 vaccine (%)
**Vaccination rate <2y (per year)**	80.5 [14.3; 100.0]	78.3 [16.2; 100.0]
**Vaccination rate 2-4y (per year)**	1.32 [0.25; 7.51]	2.55 [0.53; 13.3]
**Vaccination rate 60-74y (per year)**	–	2.07 [0.42; 10.5]
**Vaccination rate 75-84y (per year)**	–	1.71 [0.32; 9.12]
**Vaccination rate ≥85y (per year)**	–	0.14 [0.03; 0.71]
**Waning rate <2y (per year)**	2.14 [0.45; 9.77]	3.24 [0.57; 16.3]
**Waning rate 2-4y (per year)**	2.14 [0.45; 9.77]	3.24 [0.57; 16.3]
**Waning rate 60-74y (per year)**	–	2.84 [0.55; 14.8]
**Waning rate 75-84y (per year)**	–	3.53 [0.65; 18.4]
**Waning rate ≥85y (per year)**	–	3.97 [0.79; 23.1]
**Impact on carriage of *G*1 serotypes**	96.4 [84.8; 99.4]	92.2 [70.7; 98.5]
**Impact on carriage of *G*2 serotypes**	–	93.0 [71.7; 98.6]
**Impact on carriage of serotype 3**	–	41.2 [11.6; 78.2]

All parameters were assumed constant over time, except for PCV7 vaccination rates, which were set to zero upon PCV13 introduction. Note the parsimony assumption for waning of children age groups. As the model follows vaccination recommendations in Germany, no PCV7 vaccination of older individuals was assumed. Rates are given as “per year” to facilitate interpretation. Impact parameters are independent of age.

### Model predictions for pneumococcal carriage

**Fig 4:**
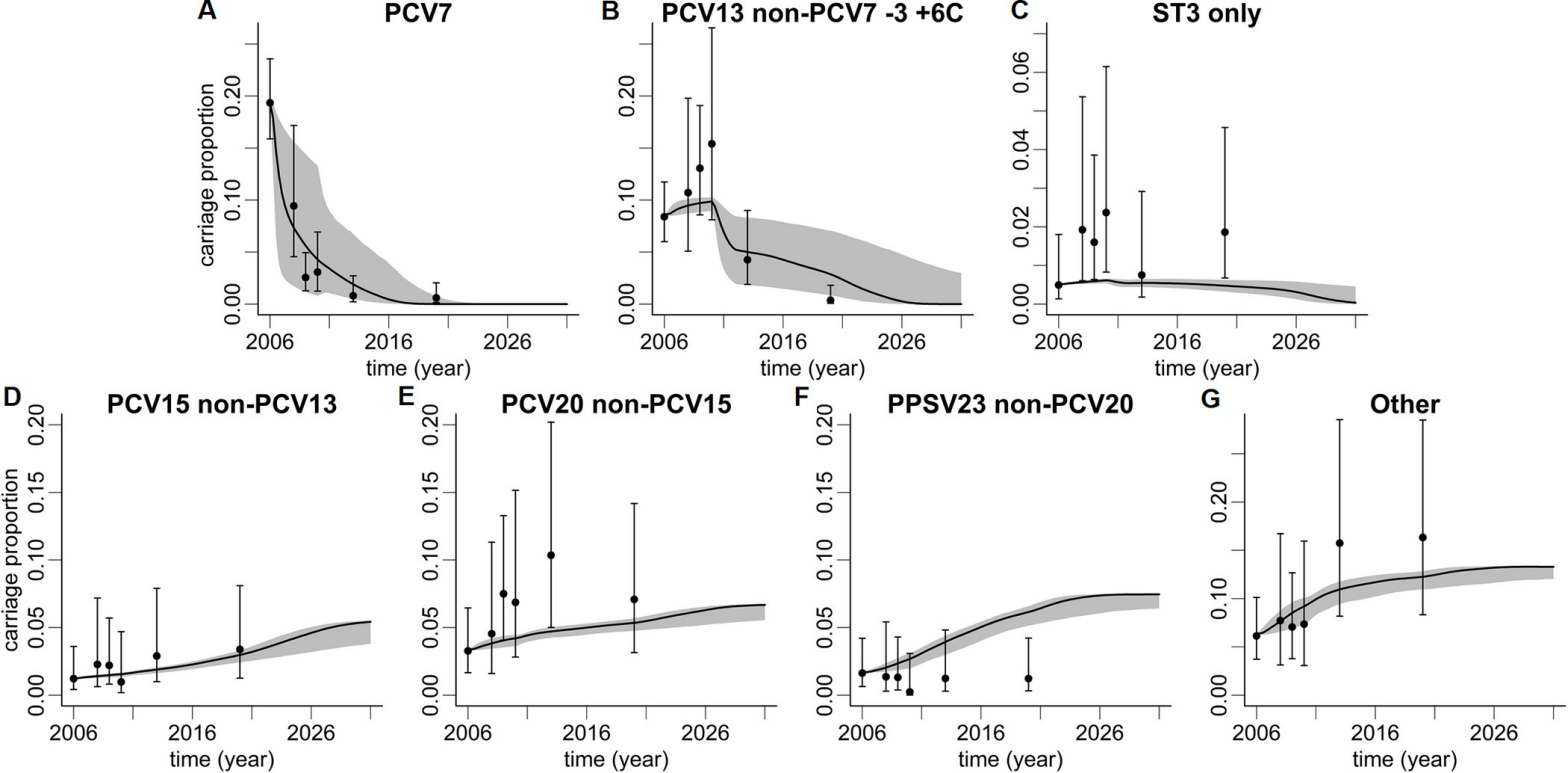
Predictions of vaccination model for children aged <2y. Shown are vaccine serotype groups (upper row) PCV7 (A), PCV13 non-PCV7–3 +6C (B), serotype 3 (C), and non-vaccine serotype groups (lower row) PCV15 non-PCV13 (D), PCV20 non-PCV15 (E), PPSV23 non-PCV20 (F), and Other (G). Data points (pneumococcal carriage proportions regarding all individuals of the age group in Germany, including non-carriers) are shown as filled circles with 95% CIs. Data points were calculated from adjusted IPD incidence rates via case-carrier ratios. Simulation results are depicted by lines with shaded 95% prediction intervals. The wide shades observed for PCV7 and PCV13 non-PCV7–3 +6C are caused by uncertainty of direct vaccination effects while the variability observed in non-vaccine serotype groups is indirect. Thus, respective prediction intervals are narrower.

Using the previously estimated parameters, we predicted future pneumococcal carriage dynamics until 2031. We focused here on two age groups. We considered children <2y of age because of the highest annual vaccination uptake among the age groups and the highest disease rates. Adults aged 60-74y were chosen as a representative age group with high pneumococcal disease burden and low rates of direct PCV13 vaccination, and thus largely reflective of indirect effects from pediatric PCV13 vaccination. The resulting model prediction for age group <2y is shown in Fig 4. Starting in 2006 with the introduction of PCV7, a continued decline of PCV7 serotype carriage was observed in model simulations and data (Fig 4A). After the introduction of pediatric PCV7 vaccination, the relative carriage of serotypes 1, 5, 6A, 6C, 7F and 19A (PCV13 non-PCV7–3 +6C serotypes) continued to increase until the introduction of PCV13 vaccination in 2009/10 (Fig 4B). Thereafter, a continued decline of PCV13 non-PCV7–3 +6C serotypes was predicted, albeit less pronounced than the decline of PCV7 serotypes. The predictions with respect to the directionality and effect size of the vaccination impact on serotype 3 carriage were not as clear (Fig 4C). While on average PCV13 vaccination was associated with a continuing slow reduction of serotype 3 in age group <2y, some simulations predicted a constant serotype 3 carriage prevalence over time, as evidenced by the upper bounds of the 95% prediction interval.

The model predicted an increase of relative carriage in all four non-vaccine serotype groups over time. Importantly, the model predicted substantial increases in the additional serotypes included in PCV15 (PCV15 non-PCV13) and serotypes only included in PCV20 (PCV20 non-PCV15) over the full model horizon until 2031 (Fig 4D and 4E). Increases in carriage prevalence were also predicted for the additional serotypes in PPSV23 (PPSV23 non-PCV20) and for other serotypes (*G*7). These increases were predicted to plateau around 2025 (Fig 4F and 4G). The relative increase was most pronounced for PCV15 non-PCV13 serotypes, resulting in an approximately four-fold higher prevalence in 2031 as compared to the 2006 baseline. Between 2006 and 2031, carriage of serotypes of the PCV15 non-PCV13 and PCV20 non-PCV15 groups combined was predicted to increase from 4% to around 12% and from 3% to 8% for children <2y and adults 60-74y of age, respectively.

**Fig 5:**
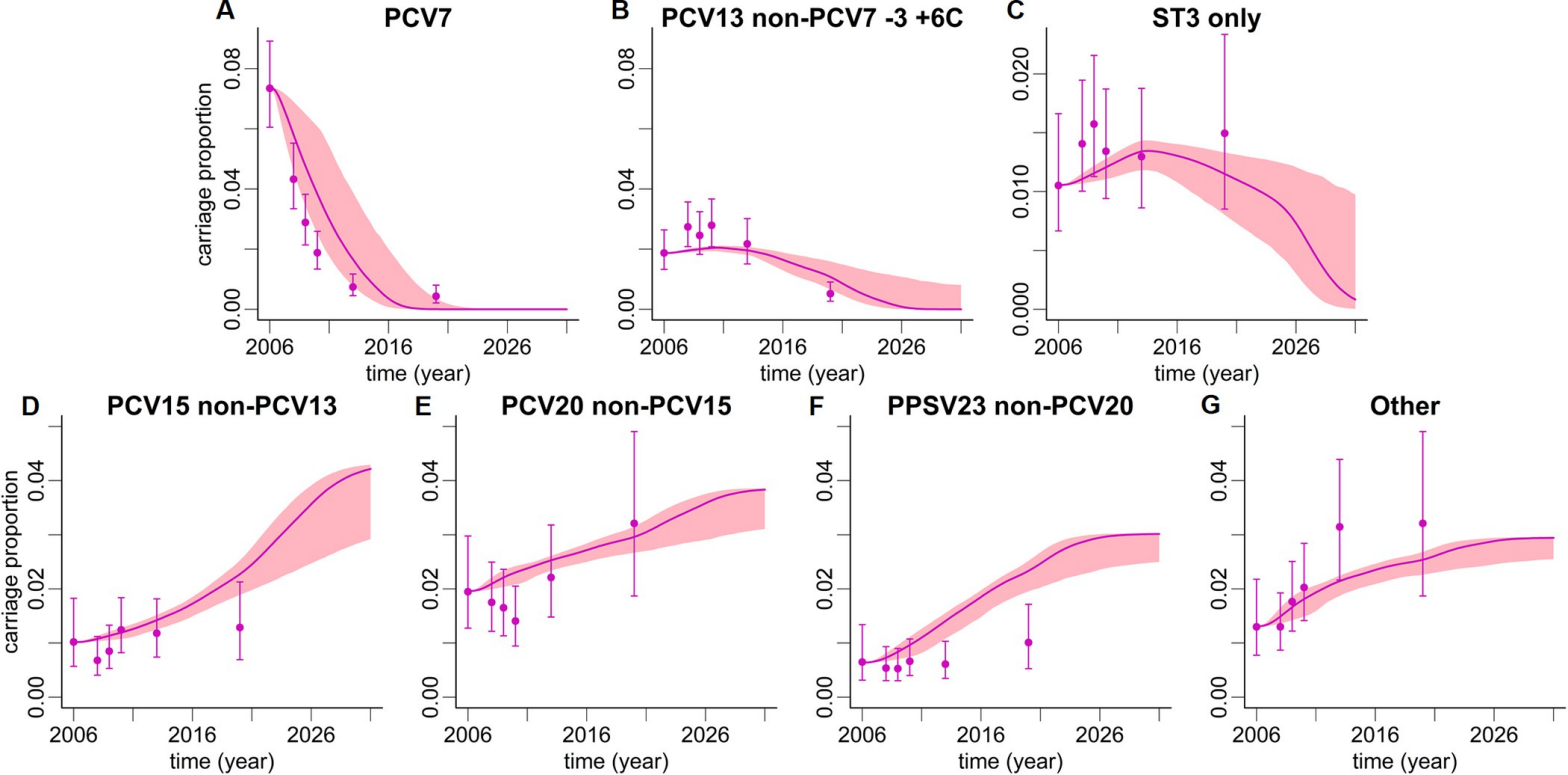
Predictions of vaccination model for adults aged 60-74y. Shown are vaccine serotype groups (upper row) PCV7 (A), PCV13 non-PCV7–3 +6C (B), serotype 3 (C), and non-vaccine serotype groups (lower row) PCV15 non-PCV13 (D), PCV20 non-PCV15 (E), PPSV23 non-PCV20 (F), and Other (G). Data points (pneumococcal carriage proportions regarding to all individuals of the age group in Germany, including non-carriers) are shown as filled circles with 95% CIs. Data points were calculated from adjusted IPD incidence rates via case-carrier ratios. Lines with shaded 95% prediction intervals depict simulation results.

Results of the vaccination model for pneumococcal carriage of adults aged 60-74y are shown in Fig 5. Although a decline in PCV7 and PCV13 non-PCV7–3 +6C serotypes with similar magnitude was observed, this effect was less pronounced than for children <2y of age. While there was generally a good agreement between model simulations and serotype carriage data, the model prediction appeared to lag behind the data points, i.e., indirect herd effects were visible more readily in the measured serotype distribution data as compared to the model, which was most pronounced for the PCV7 serotype group (Fig 5A). With respect to non-vaccine serotypes, the predictions were qualitatively similar to the <2y age group. It should be noted that total carriage was not predicted to change considerably in any age group over time.

### Model predictions for IPD

**Fig 6:**
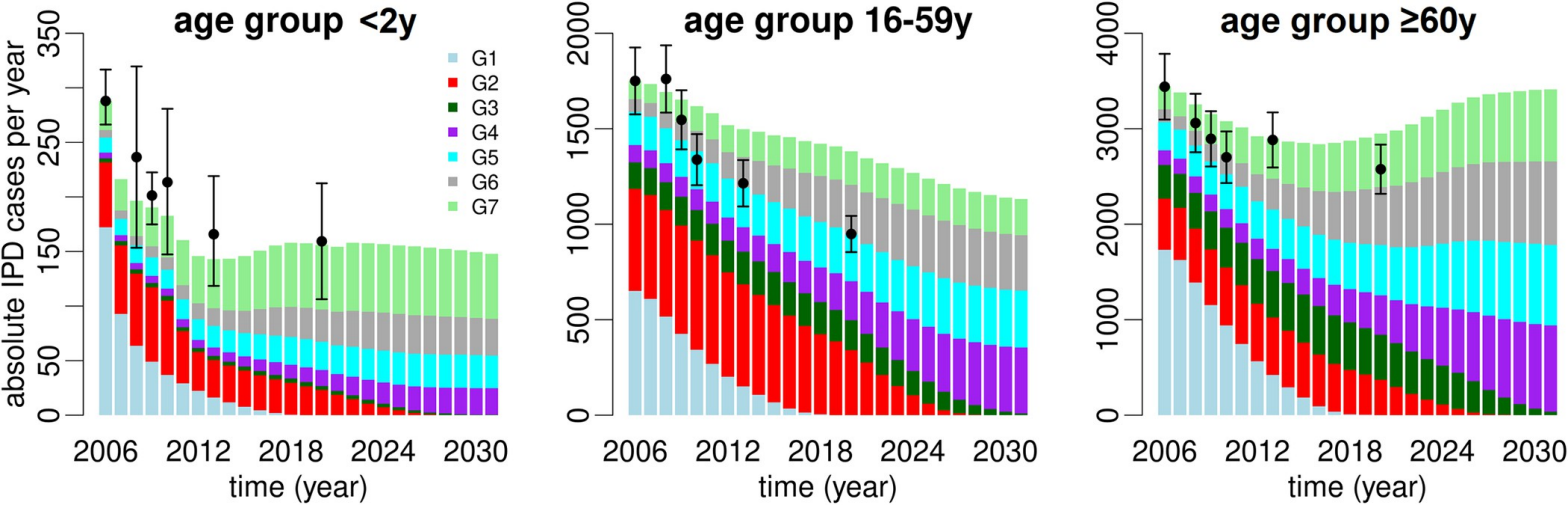
Stacked bar plots of predicted absolute IPD cases per year from 2006 to 2031 in Germany for three different age groups (<2y, 16-59y, ≥60y). The stacked bars illustrate the serotype groups defined in Table 1: PCV7 (*G*1; light blue), PCV13 non-PCV7–3 +6C (*G*2; red), serotype 3 only (*G*3; dark green), PCV15 non-PCV13 (*G*4; purple), PCV20 non-PCV15 (*G*5; cyan), PPSV23 non-PCV20 (*G*6; gray), Other serotypes (*G*7; light green). Heights of the individual bars correspond to the average model prediction without considering sensitivity of the model parameters (i.e., prediction intervals). Data points (reported IPD case numbers in Germany multiplied by an estimated year-specific underreporting factor) are shown as filled circles with 95% CIs. Age group 16-59y combines two (16-44y, 45-59y) and age group ≥60y combines three (60-74y, 75-84y, ≥85y) age groups of the model.

**Fig 7:**
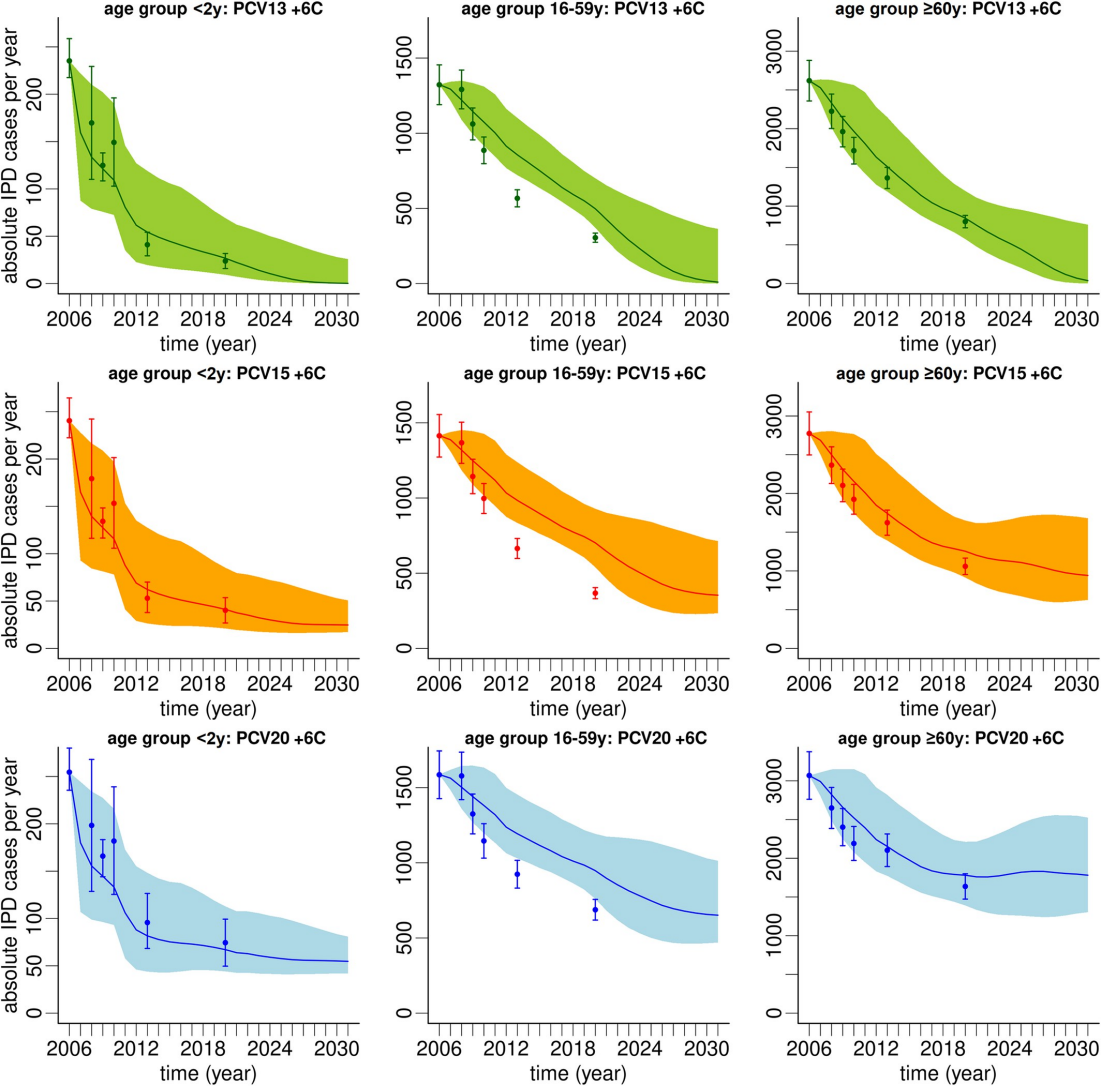
Prediction of absolute IPD cases per year from 2006 to 2031 in Germany for three different vaccine serotype groups (PCV13, PCV15, PCV20; rows) and three age groups (<2y, 16-59y, ≥60y; columns). In this figure, vaccine serotype groups are defined additively: PCV13 (green) is defined as the sum of *G*1 (PCV7), *G*2 (PCV13 non-PCV7–3 +6C), and *G*3 (serotype 3 only). PCV15: *G*1+*G*2+*G*3+*G*4 (orange), PCV20: *G*1+*G*2+*G*3+*G*4+*G*5 (blue). Serotype 6C is additionally included in each group, because it was included in the *G*2 group (Table 1). As in Fig 6, age group 16-59y combines two (16-44y, 45-59y) and age group ≥60y combines three (60-74y, 75-84y, ≥85y) age groups of the model. Data points (reported IPD cases in Germany multiplied by year-specific underreporting factor) are depicted as filled circles with 95% CIs. Average model predictions are shown by lines with shaded 95% prediction intervals accounting for sensitivity of the model parameters.

Model predictions for annual IPD case counts in Germany are shown for three different age groups: <2y, 16-59y, and ≥60y (Figs 6 and 7). With the introduction of PCV7 and PCV13, dropping overall IPD case counts were observed across all ages until 2013. Thereafter, cases were predicted to continue to decline among adults aged 16-59y but to stagnate among children <2y. For adults ≥60y a trend of decreasing IPD case numbers was reversed around 2015, and an increasing number of cases was predicted between 2016 and 2031. This led to a 1.2-fold increase (from approximately 2900 overall IPD cases in 2016 to 3450 cases in 2031) in this age group. It is also noteworthy that non-PCV13 serotypes, particularly PCV15 non-PCV13 and PCV20 non-PCV15, but also PPSV23 non-PCV20 and other (*G*7) serotypes, were predicted to play an increasing role in future IPD cases. The model predicted that in 2031, irrespective of age group, almost all new IPD cases were associated with these four serotype groups. Furthermore, serotypes included in PCV20 were predicted to cause approximately 1.8-fold more IPD cases in 2031 compared to PCV15 serotypes (Fig 7). This applied to all three depicted age groups. In contrast to a continued decline of PCV15 serotypes, the prediction average suggested stagnating case counts of PCV20-type IPD in older adults over the model horizon, while the upper bound of the prediction interval suggested increasing absolute case numbers.

We additionally performed a sensitivity analysis of the parameters representing serotype competition while keeping previously estimated vaccination parameters fixed. We did not consider the full complexity, but parsimoniously assumed all 42 *c* parameters to be identical, ranging from 0 to 1 in steps of 0.1. The corresponding modeling results are shown in S7 and S8 Figs in S1 File. A large variability with respect to predicted carriage prevalences in all age and serotype groups could be observed. We also provided predictions of annual IPD case counts in Germany for these parameter settings. Assuming extreme values for competition parameters, IPD case counts were heavily under- (*c* = 0) or overestimated (*c* = 1). As compared to our standard assumption (*c* = 0.5; middle row of S9 Fig in S1 File), we observed slightly underestimated case numbers for children aged <2y if assuming *c* = 0.4 (upper row). In contrast, a better agreement between model prediction and data was obtained for adults ≥60y. For *c* = 0.6 (lower row), predicted IPD cases were overestimated for adults aged 16-59y and ≥60y. Of note, all three scenarios depicted in S9 Fig in S1 File predicted that in 2031 almost all new IPD cases were associated with the four non-vaccine serotype groups described above, irrespective of age group.

## Discussion

This study used a mathematical transmission model to predict pneumococcal serotype carriage prevalence and IPD cases specific for Germany. By taking historic pediatric vaccination with PCV7 and PCV13 into account and assuming a continuation of the current recommendations for pneumococcal vaccination in the future, we aimed to predict serotype distributions over the next ten years. Heterogeneity between serotypes was addressed by considering a total of seven different serotype groups with potentially differing properties, including, for the first time, PCV15 non-PCV13 and PCV20 non-PCV15 serotypes, which represents an extension of previous modeling approaches. Furthermore, we considered eight age groups to account for age-specific differences in serotype distributions. Our model predicted that between 2016 and 2031, IPD IRs will stagnate at current levels in children <2y of age, will further decrease in adults aged 16-59y, but will increase considerably in adults ≥60y of age. Serotype distributions in carriage and IPD are also predicted to change substantially over the modeled period, with a near eradication of PCV7 and PCV13 serotypes (excluding serotype 3) in all age groups by 2015 and 2018, respectively. The model predicted a slow but steady decline of serotype 3 carriage and IPD across all ages, even though more uncertainties were associated with this serotype. Finally, the model predicted a continuous expansion of carriage and IPD by the additional serotypes in PCV15 and PCV20 and, to a lesser degree, also by serotypes only contained in PPSV23 and serotypes included in none of the currently licensed vaccine formulations. Among the PCV formulations available for adults, PCV20 would offer the highest coverage of IPD for all ages.

Dynamic transmission models aid our understanding of the complex evolution of pneumococcal serotypes after the introduction of PCVs into pediatric vaccination programs, and predictions from these models help to estimate future vaccine preventable disease burden. In contrast to data exploration and extrapolation alone, dynamic transmission modeling can contribute to a deeper understanding of the underlying epidemiological processes. Furthermore, it can provide long-term predictions and simulations of a multitude of scenarios, e.g., regarding different vaccination rates. We therefore proposed a mathematical model of pneumococcal serotype distributions accounting for both vaccinated (PCV7/PCV13) and unvaccinated states. In our predictions, we focused on children <2y and older adults aged ≥60y. In children <2y, the modeled impact of PCV13 represents a combination of direct protection and indirect effects through interruption of transmission from children of the same or adjacent age groups [38, 39]. Due to the low uptake of PCV13 vaccination among older adults, the predicted changes in carriage and IPD in this age group are largely indirect effects from pediatric PCV vaccination. As a key public health consideration, our model predicted a steady increase of IPD in older adults over the next 10 years that is in line with similar projections by the transmission model by Kuhlmann et al. [26]. These predictions are also confirmed by observations from IPD surveillance systems in Germany [18] and other European countries that have detected a continuous increase of IPD burden among older adults since 2015 [19, 40, 41]. A possible mechanism for this increase could be a serotype distribution reshaped by the introduction of pediatric PCV use leading to diverging IPD trends between children and adults due to the higher propensity of non-PCV13 serotypes to cause disease in older adults [42]. Such higher invasiveness in adults compared to children has been reported for serotypes 8, 12F, 13, 9L, 9N, 20, and 29 [26, 42, 43]. Additionally, our model predicted that the projected upward shift in age distribution in an aging society like Germany contributes to approximately half of the overall increase of IPD burden among older adults.

Another important finding of our model is an expansion of carriage prevalence and increase of IPD cases due to non-PCV13 serotypes and most notably of the serotypes in PCV20 that are not included in PCV13. The transmission model by Kuhlmann *et al*. also predicted an increasing number of IPD cases in older adults due to non-PCV13 serotypes in Germany, but this model did not consider specific groups for serotypes included in the novel PCV15 and PCV20 vaccines [26]. Dynamics of serotype epidemiology in nasopharyngeal carriage and pneumococcal disease after PCV introduction are complex and include competition advantages for serotypes not included in PCVs as well as serotype-independent virulence factors that determine the bacterial fitness in the nasopharyngeal niche [42]. Also, higher proportions of non-vaccine serotypes prior to the introduction of pediatric PCV programs seem to result in a greater magnitude of their expansion post-introduction [44]. Furthermore, some serotypes like 1, 5, 7F, 12F, and 18C undergo multiyear epidemic cycles that are independent of vaccine use [42, 44], an issue not considered in our current modeling. Regardless of the underlying mechanisms, it is noteworthy that many of the serotypes projected to increase are included in PPSV23, a vaccine that has been used with low uptake rates around 20% in Germany for many years [45]. As our model used adjusted IPD IRs as source data to infer carriage rates in adults which were then back-calculated to IPD cases via CCR, the model design allowed for impact assessments of PPSV23 vaccination among older adults in Germany. Of note, these predictions do not reflect the mechanism of action of PPSV23 vaccination, as the vaccine has no effects on carriage acquisition [46]. The lack of population-level impact of PPSV23 on vaccine-type disease has also been described in the UK, a country with a robust IPD surveillance system and PPSV23 uptake rates of >70% in the target population [40].

Our model predicted a large and growing burden of IPD in older adults due to serotypes included in next-generation PCVs, and in PCV20 in particular. When interpreting these results, one should keep in mind that our burden estimates are conservative and that the true pneumococcal burden potentially preventable by vaccines will be several times higher than projected [47]. This underestimation of the disease burden results from several sources of bias. For example, laboratory-confirmed IPD is markedly underdiagnosed in children as exemplified by a vaccine-probe study from Finland [48, 49], and similar considerations will also likely apply for adults. A recent systematic literature review has shown that relying on microbiologically confirmed pneumococcal disease outcomes tends to underestimate the public health benefits of PCVs when compared with clinically defined outcomes [50]. Furthermore, we exclusively modeled IPD cases, which represents only a small proportion of pneumococcal disease presentations that are preventable by PCVs both in adults and children [47, 51].

While our findings have important implications for planning vaccine interventions in Germany, there are some limitations to the generalizability of the model findings. Due to the SARS-CoV2 pandemic and subsequent infection containment policies, marked reductions of IPD compared to pre-pandemic levels were observed globally and in Germany as well [52]. The first case of COVID-19 in Germany was reported in January 2020, but major non-pharmaceutical interventions were not imposed until the end of March 2020 [53]. Although IPD case numbers could be affected by these restrictions between April and June 2020, we decided to neglect the pandemic influences on the 2019/20 IPD data. We did not use IPD data from the epidemiological years 2020/21 and 2021/22. A pneumococcal transmission model for England and Wales predicted COVID-19 containment measures would continue to result in profound reductions of IPD IRs in all age groups for up to five years after the end of the lockdown periods [30]. If proven true, this would question the generalizability of our model predictions. However, recent trends in IPD surveillance reported by the IRIS network and from Germany and Switzerland suggest that IPD has already returned to pre-pandemic levels during the winter of 2021/22 [54, 55], disproving the model predictions by Choi et al. [30]. Furthermore, a rebound of non-SARS CoV2 respiratory tract virus infection (e.g., RSV and influenza) above pre-pandemic levels has been projected by some epidemiological models [56, 57], and given the association of RSV and influenza infection with *S*. *pneumoniae* (co-infection), may also affect the pneumococcal disease burden [58–60].

Also, our model assumed a continuation of universal PCV13 vaccination of children in Germany until 2031. This might be an unrealistic assumption given that both PCV15 and PCV20 have already been licensed for use in adults, and PCV15 has been licensed for use in children in the EU. Furthermore, phase 3 clinical studies provided data for a pediatric indication of PCV20. However, it is unlikely that either vaccine will be implemented in Germany before 2023. As the magnitude of the indirect effects of pediatric immunization programs differs substantially by serotype, it also remains to be seen to what extent PCV20 use in children will protect unvaccinated adults against the additional serotypes [61]. Likewise, it remains speculative at this point how PCV15 and PCV20 differ in this regard. These questions need to be addressed in future studies.

Because there were no age-specific nasopharyngeal pneumococcal carriage rates available to inform the model directly, carriage rates in children and adults had to be derived from reported and adjusted IRs of IPD using grouped CCRs from the literature [28]. This approach was associated with several uncertainties and sources of bias. Firstly, IPD surveillance in children prior to the introduction of PCVs in Germany was potentially underestimating IRs due to underreporting of less severe non-meningitis cases [31]. Secondly, adjusted IPD IRs of adults in Germany have only been reported for the pre-PCV era and only for one federal state [37]. Trends after 2005 had to be extrapolated from IPD trends reported in the UK [62]. Despite these limitations, carriage prevalences estimated in this paper for the pre-PCV era in Germany were similar to those reported for England [63, 64]. In contrast, percentages of pneumococcal carriers among children were reportedly higher in Southern Europe [65, 66].

Due to lack of suitable CCR estimates in the literature, the model assumed the same CCRs for all non-PCV13 serotypes, which most likely is an oversimplification. Although serotype-specific CCRs are given in some publications [8, 63, 64, 67, 68], none of these authors provided age-specific CCRs, which are required to reliably estimate IPD case numbers for all age groups. Furthermore, due to small sample sizes, most estimates were provided with relatively broad 95% CIs. Substantial discrepancies of mean estimates between publications could also be observed. Assumptions also had to be made on the vaccine-related serotypes 6C and 15C. In contrast to the approach by Kuhlmann et al., we assumed that vaccines including the serotype 6A antigen (i.e., PCV13, PCV15, and PCV20) cross-protect against carriage and IPD due to serotype 6C. This assumption has been validated in PCV13 effectiveness and impact studies [32, 69], and the same approach has also been used in a recent pharmaco-economic model [33]. Serotype 15C, in contrast, was classified in our model as a G7 (i.e., other) serotype, as data on the real-world effectiveness of PCV20 against serotype 15C are missing and cross-protection is currently only supported by the structural relatedness of the 15B and 15C capsule [70].

Furthermore, our model had a limited ability to predict short-term IPD trends for some of the serotype groups. While its predictions of near elimination of PCV7 serotypes were largely reflective of observed trends in IPD both among children and adults [18], the model failed to predict the persistent IPD burden due to the five additional PCV13 serotypes 1, 5, 7F, 6A, and 19A. For these serotypes, the model predicted near elimination by 2018, while observed IPD case counts among older adults have remained constant since 2015 with PCV13-type IPD (excluding serotype 3) contributing to about 15% of IPD cases in this age group [18, 55]. In this regard, our model matched the Kuhlmann model, which also predicted near elimination of the additional PCV13 serotypes [26]. The suboptimal prediction of PCV13-type carriage and IPD might arise from differences with respect to vaccine impact on carriage of specific serotypes within individual serotype groups and oversimplifying assumptions for the aggregated impact. Also, we assumed that CCRs are causally related to the serotype and remain constant over time. Increasing evidence suggests, however, that relationships might be more indirect where serotypes are associated with genetic loci like antimicrobial resistance genes promoting bacterial fitness unrelated to capsule expression [71].

Model predictions around the burden of serotype 3 were also not well aligned with observed IPD trends in Germany. However, interpretation was complicated by the broad 95% CIs associated with estimates of serotype 3 carriage in the German population. The model predicted a stagnant to declining serotype carriage in children <2y and declining carriage and IPD cases in older adults, although the predictive uncertainty in older adults was substantial. If the impact of PCV13 vaccinations on serotype 3 carriage is assumed to be zero (which is not included in the estimated confidence interval), IPD cases due to serotype 3 are predicted to increase. These model predictions differ from the results of Kuhlmann *et al*., which projected an unchanged serotype 3 IPD burden in the years to come [26]. Predictions of the latter model align well with the observed serotype 3 IPD burden, which has remained constant around 20% among adults ≥60y of age since 2015 [18, 55]. Although PCV13 protection against serotype 3 is supported by several lines of evidence, available data are not without conflict. In the CAPiTA trial [72], PCV13 efficacy against CAP due to serotype 3 was consistent with the overall PCV13 efficacy against vaccine-type CAP. A recent meta-analysis that evaluated PCV13 effectiveness against IPD caused by serotype 3 in children also reported significant protection [73], while a large test-negative design study from the UK concluded that PCV13 vaccination was not protective [32]. PCV13 also did not protect against serotype 3 carriage acquisition in a randomized controlled trial that evaluated PCV13 efficacy in Israeli children, but effects on carriage could not be excluded by the study [74]. If serotype 3 disease rates are compared between countries that have used PCV10 (a vaccine that does not contain serotype 3) and countries using PCV13 in their pediatric immunization programs, lower serotype 3 IPD rates are found in countries that use PCV13, suggesting that the vaccine provides protection against this serotype [75]. Lastly, serotype 3 vaccine impact is potentially reduced by the recent spread of serotype 3 clades that are better adapted to survival in the nasopharyngeal niche and thus more efficient to circulate and transmit in the population [76].

Generally, when interpreting potentially suboptimal agreement between model predictions and data, it needs to be kept in mind that we only fitted a small subset of parameters of the vaccination model to the data (vaccination and waning rates in a few age groups) to avoid overfitting. The vast majority of parameters (estimated for the epidemiological model) was kept constant. As illustrated by our sensitivity analysis of the competition parameters, a much better agreement between simulations and data can be achieved with additional parameter fittings. However, we decided against too many degrees of freedom since the available data base is too small to support full-scale parameter fittings purely based on epidemiological data.

Competition between serotypes in nasopharyngeal carriage is currently incompletely understood. In principle, multiple serotype carriage is inversely correlated to intraspecific competition causing one pneumococcal serotype to exclude others from the host [71]. However, the detection of multiple serotype carriage is highly dependent on the measurement method [77]. With more sensitive techniques such as microarray-based molecular serotyping, a much higher proportion of co-colonization of different serotypes is found compared to culture-based methods [78]. The biological significance of this observation for the transmission dynamics of *S*. *pneumoniae* remains unclear. We therefore took a conservative approach and kept competition parameters constant at 0.5.

In summary, the results from this dynamic pneumococcal transmission model for Germany suggest an increasing burden of IPD in adults, of which the majority is due to serotypes that are included in PCV20. Given that results from the phase 3 clinical development program of PCV20 support the assumption that this vaccine will have an effectiveness similar to PCV13 in the prevention of pneumonia and IPD due to vaccine serotypes [24, 72, 79], direct vaccination with PCV20 has the strong potential to offer broad protection against pneumococcal disease in older adults.

## Supporting information

S1 File This file includes S1-S6 Tables, S1-S9 Figs as well as model equations, model parameters, and additional technical details. (DOCX)
