# Supplementary material for: Mathematical modeling of pneumococcal transmission dynamics in response to PCV13 infant vaccination in Germany predicts increasing IPD burden due to serotypes included in next-generation PCVs
Source: PLoS One. 2023 Feb 15;18(2):e0281261. doi: 10.1371/journal.pone.0281261 (PMC9931105; doi:10.1371/journal.pone.0281261)
Supplement: S1 File — (DOCX) [file pone.0281261.s001.docx]

**Mathematical modeling of pneumococcal transmission dynamics in response to PCV13 infant vaccination in Germany predicts increasing IPD burden due to serotypes included in next-generation PCVs**

Matthias Horn,^1^* Christian Theilacker,^2^ Ralf Sprenger,^2^ Christof von Eiff,^2^ Ernestine Mahar,^2^ Julia Schiffner-Rohe,^2^ Mathias W. Pletz,^3^ Mark van der Linden,^4^ and Markus Scholz^1^

^1^Institute for Medical Informatics, Statistics and Epidemiology, University of Leipzig, Leipzig, Germany; ^2^Pfizer Pharma GmbH, Germany; ^3^Institute for Infectious Diseases and Infection Control, Jena University Hospital, Jena, Germany; ^4^Institute of Medical Microbiology, German National Reference Centre for Streptococci, University Hospital RWTH Aachen, Aachen, Germany

*Corresponding author: Dr. Matthias Horn, Institute for Medical Informatics, Statistics and Epidemiology, University of Leipzig, Haertelstrasse 16–18, 04107 Leipzig, Germany; e-mail: matthias.horn@imise.uni-leipzig.de; phone: +49 341 97 16193; fax: +49 341 97 16109

**Supporting Information**

**1. Model parameters**

Our mathematical model of pneumococcal serotype (ST) dynamics in Germany (a detailed description of model structure and assumptions is provided in the Material and Methods section of the main manuscript) consists of three submodels, (i) an epidemiological model (excluding vaccine effects), (ii) a pneumococcal conjugate vaccine (PCV) model, and (iii) a demographic model of population dynamics and its age structure in Germany. Model parameters required for (i) are given in S1 Table.

Due to parsimony assumptions the 400 age strata were summarized to eight different age groups representing different parameter settings, namely <2y, 2-4y, 5-15y, 16-44y, 45-59y, 60-74y, 75-84y, ≥85y. For example, with respect to the 56 transmission probabilities $\alpha$ given in S5 Table, the following equalities were assumed. (Here, $k=1,\ldots,7$denote the ST groups, while numbers in parentheses $\left( 1 \right),\ldots, \left( 8 \right)$ correspond to age groups <2y, …, ≥85y, respectively.)

$$\alpha_{\boldsymbol{Gk}_{(1)}}=\alpha_{\boldsymbol{Gk}_{1}}=\text{…}=\alpha_{\boldsymbol{Gk}_{8}}$$

$$\alpha_{\boldsymbol{Gk}_{(2)}}=\alpha_{\boldsymbol{Gk}_{9}}=\text{…}=\alpha_{\boldsymbol{Gk}_{20}}$$

$$\alpha_{\boldsymbol{Gk}_{(3)}}=\alpha_{\boldsymbol{Gk}_{21}}=\text{…}=\alpha_{\boldsymbol{Gk}_{64}}$$

$$\alpha_{\boldsymbol{Gk}_{(4)}}=\alpha_{\boldsymbol{Gk}_{65}}=\text{…}=\alpha_{\boldsymbol{Gk}_{180}}$$

$$\alpha_{\boldsymbol{Gk}_{(5)}}=\alpha_{\boldsymbol{Gk}_{181}}=\text{…}=\alpha_{\boldsymbol{Gk}_{240}}$$

$$\alpha_{\boldsymbol{Gk}_{(6)}}=\alpha_{\boldsymbol{Gk}_{241}}=\text{…}=\alpha_{\boldsymbol{Gk}_{300}}$$

$$\alpha_{\boldsymbol{Gk}_{(7)}}=\alpha_{\boldsymbol{Gk}_{301}}=\text{…}=\alpha_{\boldsymbol{Gk}_{340}}$$

$$\alpha_{\boldsymbol{Gk}_{(8)}}=\alpha_{\boldsymbol{Gk}_{341}}=\text{…}=\alpha_{\boldsymbol{Gk}_{400}}$$

The basic epidemiological model was amended by a vaccination model (ii), describing the impact of two different vaccines (PCV7 and PCV13) on ST carriage dynamics. In addition to the parameters given above, the vaccination model was described by age-dependent vaccination (denoted as $\pi$) and waning rates ($\omega$), respectively. Additionally, we introduced parameters ($\nu$), which describe the impact of a vaccine on carriage of a given ST group. An overview can be found in S2 Table.

Values of the model parameters are given in the main manuscript, except for rates of carriage loss $r_{i}$ (see S3 Table), social contact matrix $\beta_{ij}$(S4 Table), and transmission probabilities $\alpha$ (S5 Table).

The demographic submodel (iii) applied pre-calculated age distributions (irrespective of gender) in Germany as reported for each calendar year by the German Federal Office of Statistics (<https://www-genesis.destatis.de/genesis/online>). While past data could be used directly, several forecasts of future age distributions are available. These forecasts vary depending on different population parameters. We here assumed an increased life expectancy until the year 2060, namely 84.4 years for males and 88.1 years for females born in 2060. We also assumed a birth rate of 1.55 children per woman, and a migration net-balance of +221,000 on yearly average [1]. For example, the age distributions in the years 2006 and 2030 are depicted in S1 Fig. For calculating incidence rates (IRs) for a particular epidemiological year (e.g., 2006/07), age distributions of the first calendar year (e.g., 2006) were used.

| **Parameter** | **Description** | **Value(s)** |
| --- | --- | --- |
| $t$ | simulation time (days) | independent variable |
| $m$ | number of age strata | 400 |
| $a$ | number of age groups | 8 |
| $n$ | number of ST groups | 7 |
| $i$ | age stratum | $1,\ldots,m$ |
| $\boldsymbol{S}_{i}(t)$ | susceptibles (i.e., non-carriers) in age stratum $i$ at time $t$ | variable |
| ${\boldsymbol{G}\boldsymbol{1}}_{i}(t)$ | exclusive carriers of $\boldsymbol{G}\boldsymbol{1}$ STs in age stratum $i$ at time $t$ | variable |
| ${\boldsymbol{G}\boldsymbol{2}}_{i}(t)$ | exclusive carriers of $\boldsymbol{G}\boldsymbol{2}$ STs in age stratum $i$ at time $t$ | variable |
| ${\boldsymbol{G}\boldsymbol{3}}_{i}(t)$ | exclusive carriers of $\boldsymbol{G}\boldsymbol{3}$ STs in age stratum $i$ at time $t$ | variable |
| ${\boldsymbol{G}\boldsymbol{4}}_{i}(t)$ | exclusive carriers of $\boldsymbol{G}\boldsymbol{4}$ STs in age stratum $i$ at time $t$ | variable |
| ${\boldsymbol{G}\boldsymbol{5}}_{i}(t)$ | exclusive carriers of $\boldsymbol{G}\boldsymbol{5}$ STs in age stratum $i$ at time $t$ | variable |
| ${\boldsymbol{G}\boldsymbol{6}}_{i}(t)$ | exclusive carriers of $\boldsymbol{G}\boldsymbol{6}$ STs in age stratum $i$ at time $t$ | variable |
| ${\boldsymbol{G}\boldsymbol{7}}_{i}(t)$ | exclusive carriers of $\boldsymbol{G}\boldsymbol{7}$ STs in age stratum $i$ at time $t$ | variable |
| ${\boldsymbol{G}\boldsymbol{1}\boldsymbol{Gk}}_{i}\left( t \right)$ | double carriers of STs of $\boldsymbol{G}\boldsymbol{1}$ and $\boldsymbol{Gk}, k=2,\ldots, 7$ in age stratum $i$ at time $t$ | variable |
| ${\boldsymbol{G}\boldsymbol{2}\boldsymbol{Gk}}_{i}\left( t \right)$ | double carriers of STs of $\boldsymbol{G}\boldsymbol{2}$ and $\boldsymbol{Gk}, k=3, \ldots, 7$in age stratum $i$ at time $t$ | variable |
| ${\boldsymbol{G}\boldsymbol{3}\boldsymbol{Gk}}_{i}\left( t \right)$ | double carriers of STs of $\boldsymbol{G}\boldsymbol{3}$ and $\boldsymbol{Gk}, k=4, \ldots, 7$ in age stratum $i$ at time $t$ | variable |
| ${\boldsymbol{G}\boldsymbol{4}\boldsymbol{Gk}}_{i}\left( t \right)$ | double carriers of STs of $\boldsymbol{G}\boldsymbol{4}$ and $\boldsymbol{Gk}, k=5, 6, 7$ in age stratum $i$ at time $t$ | variable |
| ${\boldsymbol{G}\boldsymbol{5Gk}}_{i}\left( t \right)$ | double carriers of STs of $\boldsymbol{G}\boldsymbol{5}$ and $\boldsymbol{Gk}, k=6, 7$ in age stratum $i$ at time $t$ | variable |
| ${\boldsymbol{G}\boldsymbol{6}\boldsymbol{G}\boldsymbol{7}}_{i}\left( t \right)$ | double carriers of STs of $\boldsymbol{G}\boldsymbol{6}$ and $\boldsymbol{G}\boldsymbol{7}$ in age stratum $i$ at time $t$ | variable |
| $r_{i}$ | rate of carriage loss for age stratum $i$ | S3 Table |
| $\alpha_{{\boldsymbol{G}\boldsymbol{1}}_{i}}$ | transmission probability of age stratum $i$ with respect to $\boldsymbol{G}\boldsymbol{1}$ | S5 Table |
| $\alpha_{{\boldsymbol{G}\boldsymbol{2}}_{i}}$ | transmission probability of age stratum $i$ with respect to $\boldsymbol{G}\boldsymbol{2}$ | S5 Table |
| $\alpha_{{\boldsymbol{G}\boldsymbol{3}}_{i}}$ | transmission probability of age stratum $i$ with respect to $\boldsymbol{G}\boldsymbol{3}$ | S5 Table |
| $\alpha_{{\boldsymbol{G}\boldsymbol{4}}_{i}}$ | transmission probability of age stratum $i$ with respect to $\boldsymbol{G}\boldsymbol{4}$ | S5 Table |
| $\alpha_{{\boldsymbol{G}\boldsymbol{5}}_{i}}$ | transmission probability of age stratum $i$ with respect to $\boldsymbol{G}\boldsymbol{5}$ | S5 Table |
| $\alpha_{{\boldsymbol{G}\boldsymbol{6}}_{i}}$ | transmission probability of age stratum $i$ with respect to $\boldsymbol{G}\boldsymbol{6}$ | S5 Table |
| $\alpha_{{\boldsymbol{G}\boldsymbol{7}}_{i}}$ | transmission probability of age stratum $i$ with respect to $\boldsymbol{G}\boldsymbol{7}$ | S5 Table |
| $\beta_{ij}$ | social contacts per day of age stratum $i$ with age stratum $j$ | S4 Table |
| $\lambda_{{\boldsymbol{G}\boldsymbol{1}}_{i}}(t)$ | transmission risk of age stratum $i$ with respect to $\boldsymbol{G}\boldsymbol{1}$ at time $t$ | variable |
| $\lambda_{{\boldsymbol{G}\boldsymbol{2}}_{i}}(t)$ | transmission risk of age stratum $i$ with respect to $\boldsymbol{G}\boldsymbol{2}$ at time $t$ | variable |
| $\lambda_{{\boldsymbol{G}\boldsymbol{3}}_{i}}(t)$ | transmission risk of age stratum $i$ with respect to $\boldsymbol{G}\boldsymbol{3}$ at time $t$ | variable |
| $\lambda_{{\boldsymbol{G}\boldsymbol{4}}_{i}}(t)$ | transmission risk of age stratum $i$ with respect to $\boldsymbol{G}\boldsymbol{4}$ at time $t$ | variable |
| $\lambda_{{\boldsymbol{G}\boldsymbol{5}}_{i}}(t)$ | transmission risk of age stratum $i$ with respect to $\boldsymbol{G}\boldsymbol{5}$ at time $t$ | variable |
| $\lambda_{{\boldsymbol{G}\boldsymbol{6}}_{i}}(t)$ | transmission risk of age stratum $i$ with respect to $\boldsymbol{G}\boldsymbol{6}$ at time $t$ | variable |
| $\lambda_{{\boldsymbol{G}\boldsymbol{7}}_{i}}(t)$ | transmission risk of age stratum $i$ with respect to $\boldsymbol{G}\boldsymbol{7}$ at time $t$ | variable |
| $c_{\boldsymbol{xy}}$ | competition parameters: reduction of transmission risk to be-come $\boldsymbol{Gx}$ carrier if already $\boldsymbol{Gy}$ carrier ($x, y=1,\ldots, n$; $x\neq y$) | 0.5 for all 42 $c$’s  (0 to 1, steps of 0.1) |

**S1 Table. Model parameters of the basic epidemiological model.** For a detailed description of the model see Materials and Methods section of the main manuscript. A description of ST groups $\boldsymbol{G}\boldsymbol{1}\boldsymbol{,\ldots, G}\boldsymbol{7}$ is provided in Table 1. Age strata (denoted by $i=1,\ldots,m$) were summarized to eight different age groups representing different parameter settings, namely <2y, 2-4y, 5-15y, 16-44y, 45-59y, 60-74y, 75-84y, ≥85y. Parameter values marked as “variable” were calculated at each time $t$. Note that transmission probabilities $\alpha$ (S5 Table) were obtained by fitting the model to the data.

| **Parameter** | **Description** | **Value(s)** |
| --- | --- | --- |
| ${\boldsymbol{S}\boldsymbol{v}\boldsymbol{1}}_{i}(t)$ | PCV7-vaccinated non-carriers in age stratum $i$ at time $t$ | variable |
| ${\boldsymbol{G}\boldsymbol{1}\boldsymbol{v}\boldsymbol{1}}_{i}(t)$ | PCV7-vaccinated carriers of $\boldsymbol{G}\boldsymbol{1}$ STs in age stratum $i$ at time $t$ | variable |
| ${\boldsymbol{G}\boldsymbol{2}\boldsymbol{v}\boldsymbol{1}}_{i}(t)$ | PCV7-vaccinated carriers of $\boldsymbol{G}\boldsymbol{2}$ STs in age stratum $i$ at time $t$ | variable |
| ${\boldsymbol{G}\boldsymbol{3}\boldsymbol{v}\boldsymbol{1}}_{i}(t)$ | PCV7-vaccinated carriers of $\boldsymbol{G}\boldsymbol{3}$ STs in age stratum $i$ at time $t$ | variable |
| ${\boldsymbol{G}\boldsymbol{4}\boldsymbol{v}\boldsymbol{1}}_{i}(t)$ | PCV7-vaccinated carriers of $\boldsymbol{G}\boldsymbol{4}$ STs in age stratum $i$ at time $t$ | variable |
| ${\boldsymbol{G}\boldsymbol{5}\boldsymbol{v}\boldsymbol{1}}_{i}(t)$ | PCV7-vaccinated carriers of $\boldsymbol{G}\boldsymbol{5}$ STs in age stratum $i$ at time $t$ | variable |
| ${\boldsymbol{G}\boldsymbol{6}\boldsymbol{v}\boldsymbol{1}}_{i}(t)$ | PCV7-vaccinated carriers of $\boldsymbol{G}\boldsymbol{6}$ STs in age stratum $i$ at time $t$ | variable |
| ${\boldsymbol{G}\boldsymbol{7}\boldsymbol{v}\boldsymbol{1}}_{i}(t)$ | PCV7-vaccinated carriers of $\boldsymbol{G}\boldsymbol{7}$ STs in age stratum $i$ at time $t$ | variable |
| ${\boldsymbol{G}\boldsymbol{1}\boldsymbol{Gkv}\boldsymbol{1}}_{i}\left( t \right)$ | PCV7-vaccinated double carriers of $\boldsymbol{G}\boldsymbol{1}$ and $\boldsymbol{Gk}, k=2,\ldots, 7$ in age stratum $i$ at time $t$ | variable |
| ${\boldsymbol{G}\boldsymbol{2}\boldsymbol{Gkv}\boldsymbol{1}}_{i}\left( t \right)$ | PCV7-vaccinated double carriers of $\boldsymbol{G}\boldsymbol{2}$ and $\boldsymbol{Gk}, k=3, \ldots, 7$ in age stratum $i$ at time $t$ | variable |
| ${\boldsymbol{G}\boldsymbol{3}\boldsymbol{Gkv}\boldsymbol{1}}_{i}\left( t \right)$ | PCV7-vaccinated double carriers of $\boldsymbol{G}\boldsymbol{3}$ and $\boldsymbol{Gk}, k=4, \ldots, 7$ in age stratum $i$ at time $t$ | variable |
| ${\boldsymbol{G}\boldsymbol{4}\boldsymbol{Gkv}\boldsymbol{1}}_{i}\left( t \right)$ | PCV7-vaccinated double carriers of $\boldsymbol{G}\boldsymbol{4}$ and $\boldsymbol{Gk}, k=5, 6, 7$ in age stratum $i$ at time $t$ | variable |
| ${\boldsymbol{G}\boldsymbol{5}\boldsymbol{Gkv}\boldsymbol{1}}_{i}\left( t \right)$ | PCV7-vaccinated double carriers of $\boldsymbol{G}\boldsymbol{5}$ and $\boldsymbol{Gk}, k=6, 7$ in age stratum $i$ at time $t$ | variable |
| ${\boldsymbol{G}\boldsymbol{6}\boldsymbol{G}\boldsymbol{7}\boldsymbol{v}\boldsymbol{1}}_{i}\left( t \right)$ | PCV7-vaccinated double carriers of $\boldsymbol{G}\boldsymbol{6}$ and $\boldsymbol{G}\boldsymbol{7}$ in age stratum $i$ at time $t$ | variable |
| ${\boldsymbol{S}\boldsymbol{v}\boldsymbol{2}}_{i}(t)$ | PCV13-vaccinated non-carriers in age stratum $i$ at time $t$ | variable |
| ${\boldsymbol{G}\boldsymbol{1}\boldsymbol{v}\boldsymbol{2}}_{i}(t)$ | PCV13-vaccinated carriers of $\boldsymbol{G}\boldsymbol{1}$ STs in age stratum $i$ at time $t$ | variable |
| ${\boldsymbol{G}\boldsymbol{2}\boldsymbol{v}\boldsymbol{2}}_{i}(t)$ | PCV13-vaccinated carriers of $\boldsymbol{G}\boldsymbol{2}$ STs in age stratum $i$ at time $t$ | variable |
| ${\boldsymbol{G}\boldsymbol{3}\boldsymbol{v}\boldsymbol{2}}_{i}(t)$ | PCV13-vaccinated carriers of $\boldsymbol{G}\boldsymbol{3}$ STs in age stratum $i$ at time $t$ | variable |
| ${\boldsymbol{G}\boldsymbol{4}\boldsymbol{v}\boldsymbol{2}}_{i}(t)$ | PCV13-vaccinated carriers of $\boldsymbol{G}\boldsymbol{4}$ STs in age stratum $i$ at time $t$ | variable |
| ${\boldsymbol{G}\boldsymbol{5}\boldsymbol{v}\boldsymbol{2}}_{i}(t)$ | PCV13-vaccinated carriers of $\boldsymbol{G}\boldsymbol{5}$ STs in age stratum $i$ at time $t$ | variable |
| ${\boldsymbol{G}\boldsymbol{6}\boldsymbol{v}\boldsymbol{2}}_{i}(t)$ | PCV13-vaccinated carriers of $\boldsymbol{G}\boldsymbol{6}$ STs in age stratum $i$ at time $t$ | variable |
| ${\boldsymbol{G}\boldsymbol{7}\boldsymbol{v}\boldsymbol{2}}_{i}(t)$ | PCV13-vaccinated carriers of $\boldsymbol{G}\boldsymbol{7}$ STs in age stratum $i$ at time $t$ | variable |
| ${\boldsymbol{G}\boldsymbol{1}\boldsymbol{Gkv}\boldsymbol{2}}_{i}\left( t \right)$ | PCV13-vaccinated double carriers of $\boldsymbol{G}\boldsymbol{1}$ and $\boldsymbol{Gk}, k=2,\ldots, 7$ in age stratum $i$ at time $t$ | variable |
| ${\boldsymbol{G}\boldsymbol{2}\boldsymbol{Gkv}\boldsymbol{2}}_{i}\left( t \right)$ | PCV13-vaccinated double carriers of $\boldsymbol{G}\boldsymbol{2}$ and $\boldsymbol{Gk}, k=3, \ldots, 7$ in age stratum $i$ at time $t$ | variable |
| ${\boldsymbol{G}\boldsymbol{3}\boldsymbol{Gkv}\boldsymbol{2}}_{i}\left( t \right)$ | PCV13-vaccinated double carriers of $\boldsymbol{G}\boldsymbol{3}$ and $\boldsymbol{Gk}, k=4, \ldots, 7$ in age stratum $i$ at time $t$ | variable |
| ${\boldsymbol{G}\boldsymbol{4}\boldsymbol{Gkv}\boldsymbol{2}}_{i}\left( t \right)$ | PCV13-vaccinated double carriers of $\boldsymbol{G}\boldsymbol{4}$ and $\boldsymbol{Gk}, k=5, 6, 7$ in age stratum $i$ at time $t$ | variable |
| ${\boldsymbol{G}\boldsymbol{5}\boldsymbol{Gkv}\boldsymbol{2}}_{i}\left( t \right)$ | PCV13-vaccinated double carriers of $\boldsymbol{G}\boldsymbol{5}$ and $\boldsymbol{Gk}, k=6, 7$ in age stratum $i$ at time $t$ | variable |
| ${\boldsymbol{G}\boldsymbol{6}\boldsymbol{G}\boldsymbol{7}\boldsymbol{v}\boldsymbol{2}}_{i}\left( t \right)$ | PCV13-vaccin. double carriers of $\boldsymbol{G}\boldsymbol{6}$ and $\boldsymbol{G}\boldsymbol{7}$ in age stratum $i$ at time $t$ | variable |
| $\pi_{{v1}_{i}}(t)$ | PCV7 vaccination rate in age stratum $i$ at time $t$ | Table 2 |
| $\pi_{{v2}_{i}}(t)$ | PCV13 vaccination rate in age stratum $i$ at time $t$ | Table 2 |
| $\omega_{{v1}_{i}}$ | PCV7 waning rate in age stratum $i$ | Table 2 |
| $\omega_{{v2}_{i}}$ | PCV13 waning rate in age stratum $i$ | Table 2 |
| ${\nu1}_{\boldsymbol{G}\boldsymbol{1}}$ | impact of PCV7 vaccination on carriage of $\boldsymbol{G}\boldsymbol{1}$STs | Table 2 |
| ${\nu2}_{\boldsymbol{Gk}}$ | impact of PCV13 vaccination on carriage of $\boldsymbol{G}\boldsymbol{k}$STs, $k=1,\ldots,3$ | Table 2 |

**S2 Table. Model parameter of the amended vaccination model.** For a detailed description of the model see Materials and Methods section of the main manuscript. A description of ST groups $\boldsymbol{G}\boldsymbol{1}\boldsymbol{,\ldots, G}\boldsymbol{7}$ is provided in Table 1. Vaccines denoted as $\boldsymbol{v}\boldsymbol{1}$ and $\boldsymbol{v}\boldsymbol{2}$ represent PCV7 and PCV13, respectively. Parameter values marked as “variable” were calculated at each time point $t$. Note that all vaccination parameters given in Table 2 were obtained by fitting the model to the data.

| **Parameter** | **Age** | **Parsimony (age strata)** | **Value [**$\boldsymbol{d}^{\boldsymbol{-1}}$**]** |
| --- | --- | --- | --- |
| $r_{(1)}$ | <1y | $r_{1}=r_{2}=r_{3}=r_{4}$ | 1/74 |
| $r_{(2)}$ | 1-2y | $r_{5}=\text{…}=r_{12}$ | 1/47 |
| $r_{(3)}$ | 3-4y | $r_{13}=\text{…}=r_{20}$ | 1/34 |
| $r_{(4)}$ | 5-18y | $r_{21}=\text{…}=r_{76}$ | 1/26 |
| $r_{(5)}$ | >18y | $r_{77}=\text{…}=r_{400}$ | 1/25 |

**S3 Table. Rates of carriage loss for different age groups (given in per day) according to Högberg et al. [2].** The authors investigated the duration of nasopharyngeal carriage for six age groups (<1y, 1-2y, 3-4y, 5-6y, 7-18y, >18y). However, the same value ($1/26 d^{-1}$) was reported for age groups 5-6y and 7-18y. Note that these age groups differ from the definition used in our model, which we addressed by applying different parsimony assumptions (see third column). Subscripts correspond to the 400 age strata of 0.25 years each, e.g., $r_{1}$ represents newborns.

| **Age** | **0-4** | **5-9** | **10-14** | **15-19** | **20-24** | **25-29** | **30-34** | **35-39** | **40-44** | **45-49** | **50-54** | **55-59** | **60-64** | **65-69** | **≥70** |
| --- | --- | --- | --- | --- | --- | --- | --- | --- | --- | --- | --- | --- | --- | --- | --- |
| **0-4** | 1.90 | 0.36 | 0.09 | 0.04 | 0.08 | 0.27 | 0.46 | 0.20 | 0.20 | 0.09 | 0.19 | 0.07 | 0.10 | 0.03 | 0.07 |
| **5-9** | 0.81 | 2.25 | 0.35 | 0.18 | 0.03 | 0.17 | 0.26 | 0.49 | 0.23 | 0.09 | 0.20 | 0.11 | 0.14 | 0.11 | 0.27 |
| **10-14** | 0.34 | 0.61 | 3.58 | 0.69 | 0.12 | 0.16 | 0.14 | 0.62 | 0.67 | 0.35 | 0.16 | 0.07 | 0.07 | 0.18 | 0.34 |
| **15-19** | 0.42 | 0.42 | 0.93 | 4.19 | 0.90 | 0.28 | 0.19 | 0.46 | 1.11 | 1.21 | 0.27 | 0.26 | 0.11 | 0.15 | 0.49 |
| **20-24** | 0.56 | 0.24 | 0.25 | 1.29 | 2.59 | 1.43 | 0.44 | 0.22 | 0.57 | 0.45 | 0.47 | 0.33 | 0.19 | 0.15 | 0.30 |
| **25-29** | 0.62 | 0.43 | 0.25 | 0.36 | 1.14 | 1.79 | 0.98 | 0.30 | 0.38 | 0.57 | 0.60 | 0.45 | 0.32 | 0.19 | 0.24 |
| **30-34** | 1.20 | 0.66 | 0.34 | 0.22 | 0.45 | 1.09 | 1.49 | 0.74 | 0.57 | 0.59 | 0.54 | 0.61 | 0.31 | 0.32 | 0.41 |
| **35-39** | 0.87 | 0.77 | 0.65 | 0.60 | 0.45 | 0.77 | 1.18 | 1.43 | 0.95 | 0.70 | 0.58 | 0.57 | 0.36 | 0.42 | 0.50 |
| **40-44** | 0.49 | 0.72 | 0.97 | 0.69 | 0.47 | 0.60 | 0.98 | 1.03 | 1.23 | 1.05 | 0.71 | 0.59 | 0.49 | 0.62 | 0.52 |
| **45-49** | 0.40 | 0.38 | 0.44 | 0.68 | 0.63 | 0.77 | 0.32 | 0.59 | 0.93 | 1.23 | 0.99 | 0.62 | 0.44 | 0.38 | 0.37 |
| **50-54** | 0.38 | 0.23 | 0.37 | 0.39 | 0.41 | 0.59 | 0.67 | 0.36 | 0.47 | 0.82 | 1.18 | 0.79 | 0.40 | 0.45 | 0.38 |
| **55-59** | 0.26 | 0.26 | 0.15 | 0.26 | 0.26 | 0.59 | 0.35 | 0.28 | 0.22 | 0.41 | 0.79 | 1.12 | 0.75 | 0.35 | 0.31 |
| **60-64** | 0.27 | 0.22 | 0.19 | 0.12 | 0.11 | 0.49 | 0.42 | 0.36 | 0.32 | 0.28 | 0.42 | 0.54 | 0.85 | 0.80 | 0.43 |
| **65-69** | 0.19 | 0.18 | 0.19 | 0.15 | 0.08 | 0.23 | 0.19 | 0.30 | 0.20 | 0.15 | 0.18 | 0.30 | 0.52 | 0.91 | 0.44 |
| **≥70** | 0.38 | 0.63 | 0.24 | 0.14 | 0.19 | 0.28 | 0.21 | 0.22 | 0.40 | 0.26 | 0.28 | 0.34 | 0.37 | 1.30 | 0.76 |

**S4 Table. Contact matrix of all contacts (as opposed to physical contacts only) in Germany as reported by Mossong et al. [3] in a survey-based sociological contact study.** Shown is the average absolute number of contacts per day between an individual of age group $i$ (columns) with a contact person of age group $j$ (rows). Note that the age groups investigated by the authors differ from the definition of the 400 age strata used in our model, which we addressed by redistributing the contact frequencies evenly over all age strata within a given age group.

|  | ***G*1** | ***G*2** | ***G*3** | ***G*4** | ***G*5** | ***G*6** | ***G*7** |
| --- | --- | --- | --- | --- | --- | --- | --- |
| **<2y** | 6.07**·**10^-3^ | 3.26**·**10^-3^ | 9.45**·**10^-4^ | 3.04**·**10^-3^ | 2.82**·**10^-3^ | 3.18**·**10^-3^ | 4.81**·**10^-3^ |
| **2-4y** | 9.76**·**10^-3^ | 2.78**·**10^-3^ | 3.97**·**10^-3^ | 4.84**·**10^-3^ | 1.02**·**10^-2^ | 1.51**·**10^-2^ | 1.54**·**10^-2^ |
| **5-15y** | 7.90**·**10^-3^ | 7.49**·**10^-3^ | 4.69**·**10^-3^ | 2.63**·**10^-3^ | 1.24**·**10^-3^ | 8.09**·**10^-3^ | 6.29**·**10^-3^ |
| **16-44y** | 4.75**·**10^-3^ | 6.70**·**10^-3^ | 6.62**·**10^-3^ | 6.60**·**10^-3^ | 7.19**·**10^-3^ | 3.92**·**10^-3^ | 5.53**·**10^-3^ |
| **45-59y** | 3.96**·**10^-3^ | 1.75**·**10^-3^ | 2.45**·**10^-3^ | 3.23**·**10^-3^ | 1.69**·**10^-3^ | 4.70**·**10^-3^ | 1.49**·**10^-3^ |
| **60-74y** | 9.19**·**10^-3^ | 2.87**·**10^-3^ | 6.13**·**10^-3^ | 7.98**·**10^-3^ | 7.03**·**10^-3^ | 6.65**·**10^-3^ | 6.55**·**10^-3^ |
| **75-84y** | 6.47**·**10^-3^ | 3.09**·**10^-3^ | 7.71**·**10^-3^ | 5.05**·**10^-3^ | 2.71**·**10^-3^ | 1.08**·**10^-2^ | 6.45**·**10^-3^ |
| **≥85y** | 5.07**·**10^-3^ | 6.58**·**10^-4^ | 5.66**·**10^-3^ | 4.92**·**10^-3^ | 6.77**·**10^-3^ | 2.90**·**10^-3^ | 4.40**·**10^-3^ |

**S5 Table. Results of the estimation of transmission probabilities α.** These probabilities were assumed to be constant over time but to vary between different age and serotype groups. Details on the interpretation of the parameter values and on the fitting process itself are found in the Materials and Methods section. For a description of the serotype groups see Table 1 in the main manuscript.

| **Country** | **Year** | **References** | **Carriage prevalence** |
| --- | --- | --- | --- |
| England | 2001/02 | Flasche et al. (2011) [4]  van Hoek et al. (2014) [5] | <5y: 48.4%  5-20y: 21.1%  >20y: 7.6% |
| Portugal | 2001 | Sá-Leão et al. (2009) [6]  Davis et al. (2013) [7] | ≤6y: 64.9% |
| France | 2004 | Dunais et al. (2008) [8]  Félix et al. (2021) [9] | ≤3y: 54.5% |
| Germany | 2005/06 | this publication | <2y: 40.5%  2-4y: 48.4%  60-74y: 15.2% |

**S6 Table. Selected publications of carriage prevalence point estimates obtained from naso­pharyngeal swab studies before the introduction of pneumococcal conjugate vaccines in other European countries.** To facilitate comparison, we also provided carriage prevalences in Germany estimated for this paper. “Year” refers to the year where the measurements were conducted, not the year of publication. Estimates of carriage prevalences include all pneumococcal serotypes.


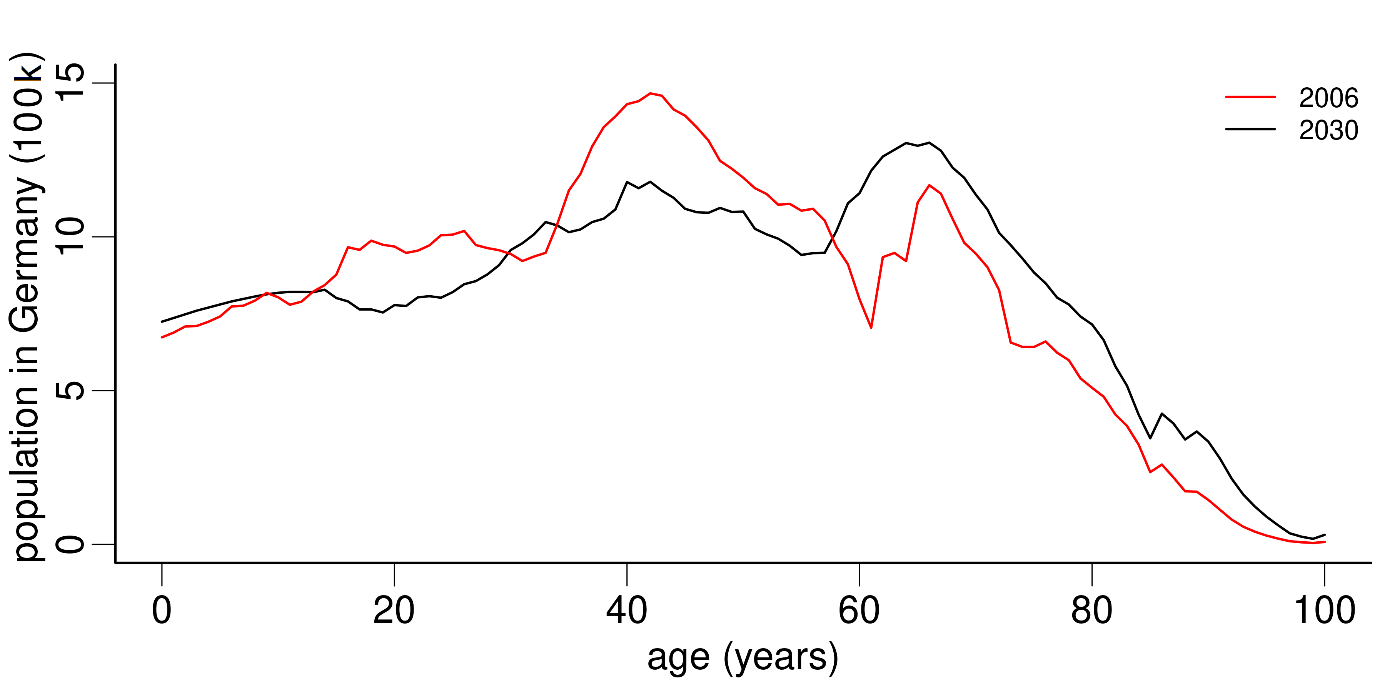


**S1 Fig. Age distributions in Germany for the years 2006 (red) and 2030 (black) as reported (2006) and forecast (2030) by the German Federal Office of Statistics.** For details on the applied forecast model see text. Note that an age of 100y denotes ≥100y, explaining the slight increase.

**2. Model equations (basic epidemiological model)**

**7 serotype groups (without vaccinations)**, $i=1,\ldots,m$ for $m=400$ age strata (3 months each)

29 compartments (non-carriers, 7 single carrier, and 21 double carrier compartments), 11.600 equations total

$$\frac{d\boldsymbol{S}_{i}(t)}{dt}=r_{i}\cdot\left( {\boldsymbol{G}\boldsymbol{1}}_{i}\left( t \right)+{\boldsymbol{G}\boldsymbol{2}}_{i}\left( t \right)+{\boldsymbol{G}\boldsymbol{3}}_{i}\left( t \right)+{\boldsymbol{G}\boldsymbol{4}}_{i}\left( t \right)+{\boldsymbol{G}\boldsymbol{5}}_{i}(t)+{\boldsymbol{G}\boldsymbol{6}}_{i}(t)+{\boldsymbol{G}\boldsymbol{7}}_{i}(t) \right)-\left( \lambda_{{\boldsymbol{G}\boldsymbol{1}}_{i}}\left( t \right)+\lambda_{{\boldsymbol{G}\boldsymbol{2}}_{i}}\left( t \right)+\lambda_{{\boldsymbol{G}\boldsymbol{3}}_{i}}\left( t \right)+\lambda_{{\boldsymbol{G}\boldsymbol{4}}_{i}}\left( t \right)+\lambda_{{\boldsymbol{G}\boldsymbol{5}}_{i}}(t)+\lambda_{{\boldsymbol{G}\boldsymbol{6}}_{i}}(t)+\lambda_{{\boldsymbol{G}\boldsymbol{7}}_{i}}(t) \right)\cdot\boldsymbol{S}_{i}(t)$$

$$\frac{d{\boldsymbol{G}\boldsymbol{1}}_{i}(t)}{dt}=\lambda_{{\boldsymbol{G}\boldsymbol{1}}_{i}}\left( t \right)\cdot\boldsymbol{S}_{i}\left( t \right)+r_{i}\cdot\left( {\boldsymbol{G}\boldsymbol{1}\boldsymbol{G}\boldsymbol{2}}_{i}\left( t \right)+{\boldsymbol{G}\boldsymbol{1}\boldsymbol{G}\boldsymbol{3}}_{i}\left( t \right)+{\boldsymbol{G}\boldsymbol{1}\boldsymbol{G}\boldsymbol{4}}_{i}\left( t \right)+{\boldsymbol{G}\boldsymbol{1}\boldsymbol{G}\boldsymbol{5}}_{i}(t)+{\boldsymbol{G}\boldsymbol{1}\boldsymbol{G}\boldsymbol{6}}_{i}(t)+{\boldsymbol{G}\boldsymbol{1}\boldsymbol{G}\boldsymbol{7}}_{i}(t) \right)-\left( c_{\boldsymbol{21}}\cdot\lambda_{{\boldsymbol{G}\boldsymbol{2}}_{i}}\left( t \right)+c_{\boldsymbol{31}}\cdot\lambda_{{\boldsymbol{G}\boldsymbol{3}}_{i}}\left( t \right)+c_{\boldsymbol{41}}\cdot\lambda_{{\boldsymbol{G}\boldsymbol{4}}_{i}}\left( t \right)+c_{\boldsymbol{51}}\cdot\lambda_{{\boldsymbol{G}\boldsymbol{5}}_{i}}\left( t \right)+c_{\boldsymbol{61}}\cdot\lambda_{{\boldsymbol{G}\boldsymbol{6}}_{i}}\left( t \right)+c_{\boldsymbol{71}}\cdot\lambda_{{\boldsymbol{G}\boldsymbol{7}}_{i}}\left( t \right)+r_{i} \right)\cdot{\boldsymbol{G}\boldsymbol{1}}_{i}(t)$$

$$\frac{d{\boldsymbol{G}\boldsymbol{2}}_{i}(t)}{dt}=\lambda_{{\boldsymbol{G}\boldsymbol{2}}_{i}}\left( t \right)\cdot\boldsymbol{S}_{i}\left( t \right)+r_{i}\cdot\left( {\boldsymbol{G}\boldsymbol{1}\boldsymbol{G}\boldsymbol{2}}_{i}\left( t \right)+{\boldsymbol{G}\boldsymbol{2}\boldsymbol{G}\boldsymbol{3}}_{i}\left( t \right)+{\boldsymbol{G2}\boldsymbol{G}\boldsymbol{4}}_{i}\left( t \right)+{\boldsymbol{G}\boldsymbol{2}\boldsymbol{G}\boldsymbol{5}}_{i}(t)+{\boldsymbol{G}\boldsymbol{2}\boldsymbol{G}\boldsymbol{6}}_{i}(t)+{\boldsymbol{G}\boldsymbol{2}\boldsymbol{G}\boldsymbol{7}}_{i}(t) \right)-\left( c_{\boldsymbol{12}}\cdot\lambda_{{\boldsymbol{G}\boldsymbol{1}}_{i}}\left( t \right)+c_{\boldsymbol{32}}\cdot\lambda_{{\boldsymbol{G}\boldsymbol{3}}_{i}}\left( t \right)+c_{\boldsymbol{42}}\cdot\lambda_{{\boldsymbol{G}\boldsymbol{4}}_{i}}\left( t \right)+c_{\boldsymbol{52}}\cdot\lambda_{{\boldsymbol{G}\boldsymbol{5}}_{i}}\left( t \right)+c_{\boldsymbol{62}}\cdot\lambda_{{\boldsymbol{G}\boldsymbol{6}}_{i}}\left( t \right)+c_{\boldsymbol{72}}\cdot\lambda_{{\boldsymbol{G}\boldsymbol{7}}_{i}}\left( t \right)+r_{i} \right)\cdot{\boldsymbol{G}\boldsymbol{2}}_{i}(t)$$

$$\frac{d{\boldsymbol{G}\boldsymbol{3}}_{i}(t)}{dt}=\lambda_{{\boldsymbol{G}\boldsymbol{3}}_{i}}\left( t \right)\cdot\boldsymbol{S}_{i}\left( t \right)+r_{i}\cdot\left( {\boldsymbol{G}\boldsymbol{1}\boldsymbol{G}\boldsymbol{3}}_{i}\left( t \right)+{\boldsymbol{G}\boldsymbol{2}\boldsymbol{G}\boldsymbol{3}}_{i}\left( t \right)+{\boldsymbol{G}\boldsymbol{3}\boldsymbol{G}\boldsymbol{4}}_{i}\left( t \right)+{\boldsymbol{G}\boldsymbol{3}\boldsymbol{G}\boldsymbol{5}}_{i}(t)+{\boldsymbol{G}\boldsymbol{3}\boldsymbol{G}\boldsymbol{6}}_{i}(t)+{\boldsymbol{G}\boldsymbol{3}\boldsymbol{G}\boldsymbol{7}}_{i}(t) \right)-\left( c_{\boldsymbol{13}}\cdot\lambda_{{\boldsymbol{G}\boldsymbol{1}}_{i}}\left( t \right)+c_{\boldsymbol{23}}\cdot\lambda_{{\boldsymbol{G}\boldsymbol{2}}_{i}}\left( t \right)+c_{\boldsymbol{43}}\cdot\lambda_{{\boldsymbol{G}\boldsymbol{4}}_{i}}\left( t \right)+c_{\boldsymbol{53}}\cdot\lambda_{{\boldsymbol{G}\boldsymbol{5}}_{i}}\left( t \right)+c_{\boldsymbol{63}}\cdot\lambda_{{\boldsymbol{G}\boldsymbol{6}}_{i}}\left( t \right)+c_{\boldsymbol{73}}\cdot\lambda_{{\boldsymbol{G}\boldsymbol{7}}_{i}}\left( t \right)+r_{i} \right)\cdot{\boldsymbol{G}\boldsymbol{3}}_{i}(t)$$

$$\frac{d{\boldsymbol{G}\boldsymbol{4}}_{i}(t)}{dt}=\lambda_{{\boldsymbol{G}\boldsymbol{4}}_{i}}\left( t \right)\cdot\boldsymbol{S}_{i}\left( t \right)+r_{i}\cdot\left( {\boldsymbol{G}\boldsymbol{1}\boldsymbol{G}\boldsymbol{4}}_{i}\left( t \right)+{\boldsymbol{G}\boldsymbol{2}\boldsymbol{G}\boldsymbol{4}}_{i}\left( t \right)+{\boldsymbol{G}\boldsymbol{3}\boldsymbol{G}\boldsymbol{4}}_{i}\left( t \right)+{\boldsymbol{G4}\boldsymbol{G}\boldsymbol{5}}_{i}(t)+{\boldsymbol{G}\boldsymbol{4}\boldsymbol{G}\boldsymbol{6}}_{i}(t)+{\boldsymbol{G}\boldsymbol{4}\boldsymbol{G}\boldsymbol{7}}_{i}(t) \right)-\left( c_{\boldsymbol{14}}\cdot\lambda_{{\boldsymbol{G}\boldsymbol{1}}_{i}}\left( t \right)+c_{\boldsymbol{24}}\cdot\lambda_{{\boldsymbol{G}\boldsymbol{2}}_{i}}\left( t \right)+c_{\boldsymbol{34}}\cdot\lambda_{{\boldsymbol{G}\boldsymbol{3}}_{i}}\left( t \right)+c_{\boldsymbol{54}}\cdot\lambda_{{\boldsymbol{G}\boldsymbol{5}}_{i}}\left( t \right)+c_{\boldsymbol{64}}\cdot\lambda_{{\boldsymbol{G}\boldsymbol{6}}_{i}}\left( t \right)+c_{\boldsymbol{74}}\cdot\lambda_{{\boldsymbol{G}\boldsymbol{7}}_{i}}\left( t \right)+r_{i} \right)\cdot{\boldsymbol{G}\boldsymbol{4}}_{i}(t)$$

$$\frac{d{\boldsymbol{G}\boldsymbol{5}}_{i}(t)}{dt}=\lambda_{{\boldsymbol{G}\boldsymbol{5}}_{i}}\left( t \right)\cdot\boldsymbol{S}_{i}\left( t \right)+r_{i}\cdot\left( {\boldsymbol{G}\boldsymbol{1}\boldsymbol{G}\boldsymbol{5}}_{i}\left( t \right)+{\boldsymbol{G}\boldsymbol{2}\boldsymbol{G}\boldsymbol{5}}_{i}\left( t \right)+{\boldsymbol{G}\boldsymbol{3}\boldsymbol{G}\boldsymbol{5}}_{i}\left( t \right)+{\boldsymbol{G}\boldsymbol{4}\boldsymbol{G}\boldsymbol{5}}_{i}(t)+{\boldsymbol{G}\boldsymbol{5}\boldsymbol{G}\boldsymbol{6}}_{i}(t)+{\boldsymbol{G}\boldsymbol{5}\boldsymbol{G}\boldsymbol{7}}_{i}(t) \right)-\left( c_{\boldsymbol{15}}\cdot\lambda_{{\boldsymbol{G}\boldsymbol{1}}_{i}}\left( t \right)+c_{\boldsymbol{25}}\cdot\lambda_{{\boldsymbol{G}\boldsymbol{2}}_{i}}\left( t \right)+c_{\boldsymbol{35}}\cdot\lambda_{{\boldsymbol{G}\boldsymbol{3}}_{i}}\left( t \right)+c_{\boldsymbol{45}}\cdot\lambda_{{\boldsymbol{G}\boldsymbol{4}}_{i}}\left( t \right)+c_{\boldsymbol{65}}\cdot\lambda_{{\boldsymbol{G}\boldsymbol{6}}_{i}}\left( t \right)+c_{\boldsymbol{75}}\cdot\lambda_{{\boldsymbol{G}\boldsymbol{7}}_{i}}\left( t \right)+r_{i} \right)\cdot{\boldsymbol{G}\boldsymbol{5}}_{i}(t)$$

$$\frac{d{\boldsymbol{G}\boldsymbol{6}}_{i}(t)}{dt}=\lambda_{{\boldsymbol{G}\boldsymbol{6}}_{i}}\left( t \right)\cdot\boldsymbol{S}_{i}\left( t \right)+r_{i}\cdot\left( {\boldsymbol{G}\boldsymbol{1}\boldsymbol{G}\boldsymbol{6}}_{i}\left( t \right)+{\boldsymbol{G}\boldsymbol{2}\boldsymbol{G}\boldsymbol{6}}_{i}\left( t \right)+{\boldsymbol{G}\boldsymbol{3}\boldsymbol{G}\boldsymbol{6}}_{i}\left( t \right)+{\boldsymbol{G}\boldsymbol{4}\boldsymbol{G}\boldsymbol{6}}_{i}(t)+{\boldsymbol{G5}\boldsymbol{G}\boldsymbol{6}}_{i}(t)+{\boldsymbol{G}\boldsymbol{6}\boldsymbol{G}\boldsymbol{7}}_{i}(t) \right)-\left( c_{\boldsymbol{16}}\cdot\lambda_{{\boldsymbol{G}\boldsymbol{1}}_{i}}\left( t \right)+c_{\boldsymbol{26}}\cdot\lambda_{{\boldsymbol{G}\boldsymbol{2}}_{i}}\left( t \right)+c_{\boldsymbol{36}}\cdot\lambda_{{\boldsymbol{G}\boldsymbol{3}}_{i}}\left( t \right)+c_{\boldsymbol{46}}\cdot\lambda_{{\boldsymbol{G}\boldsymbol{4}}_{i}}\left( t \right)+c_{\boldsymbol{56}}\cdot\lambda_{{\boldsymbol{G}\boldsymbol{5}}_{i}}\left( t \right)+c_{\boldsymbol{76}}\cdot\lambda_{{\boldsymbol{G}\boldsymbol{7}}_{i}}\left( t \right)+r_{i} \right)\cdot{\boldsymbol{G}\boldsymbol{6}}_{i}(t)$$

$$\frac{d{\boldsymbol{G}\boldsymbol{7}}_{i}(t)}{dt}=\lambda_{{\boldsymbol{G}\boldsymbol{7}}_{i}}\left( t \right)\cdot\boldsymbol{S}_{i}\left( t \right)+r_{i}\cdot\left( {\boldsymbol{G}\boldsymbol{1}\boldsymbol{G}\boldsymbol{7}}_{i}\left( t \right)+{\boldsymbol{G}\boldsymbol{2}\boldsymbol{G}\boldsymbol{7}}_{i}\left( t \right)+{\boldsymbol{G}\boldsymbol{3}\boldsymbol{G}\boldsymbol{7}}_{i}\left( t \right)+{\boldsymbol{G}\boldsymbol{4}\boldsymbol{G}\boldsymbol{7}}_{i}(t)+{\boldsymbol{G}\boldsymbol{5}\boldsymbol{G7}}_{i}(t)+{\boldsymbol{G}\boldsymbol{6}\boldsymbol{G}\boldsymbol{7}}_{i}(t) \right)-\left( c_{\boldsymbol{17}}\cdot\lambda_{{\boldsymbol{G}\boldsymbol{1}}_{i}}\left( t \right)+c_{\boldsymbol{27}}\cdot\lambda_{{\boldsymbol{G}\boldsymbol{2}}_{i}}\left( t \right)+c_{\boldsymbol{37}}\cdot\lambda_{{\boldsymbol{G}\boldsymbol{3}}_{i}}\left( t \right)+c_{\boldsymbol{47}}\cdot\lambda_{{\boldsymbol{G}\boldsymbol{4}}_{i}}\left( t \right)+c_{\boldsymbol{57}}\cdot\lambda_{{\boldsymbol{G}\boldsymbol{5}}_{i}}\left( t \right)+c_{\boldsymbol{67}}\cdot\lambda_{{\boldsymbol{G}\boldsymbol{6}}_{i}}\left( t \right)+r_{i} \right)\cdot{\boldsymbol{G}\boldsymbol{7}}_{i}(t)$$

$$\frac{d{\boldsymbol{G}\boldsymbol{1}\boldsymbol{G}\boldsymbol{2}}_{i}\left( t \right)}{dt}={c_{\boldsymbol{21}}\cdot\lambda_{{\boldsymbol{G}\boldsymbol{2}}_{i}}\left( t \right)\cdot{\boldsymbol{G}\boldsymbol{1}}_{i}\left( t \right)+c}_{\boldsymbol{12}}\cdot\lambda_{{\boldsymbol{G}\boldsymbol{1}}_{i}}\left( t \right)\cdot{\boldsymbol{G}\boldsymbol{2}}_{i}\left( t \right)-2\cdot r_{i}\cdot{\boldsymbol{G}\boldsymbol{1}\boldsymbol{G}\boldsymbol{2}}_{i}\left( t \right)$$

$$\frac{d{\boldsymbol{G}\boldsymbol{1}\boldsymbol{G}\boldsymbol{3}}_{i}\left( t \right)}{dt}={c_{\boldsymbol{31}}\cdot\lambda_{{\boldsymbol{G}\boldsymbol{3}}_{i}}\left( t \right)\cdot{\boldsymbol{G}\boldsymbol{1}}_{i}\left( t \right)+c}_{\boldsymbol{13}}\cdot\lambda_{{\boldsymbol{G}\boldsymbol{1}}_{i}}\left( t \right)\cdot{\boldsymbol{G}\boldsymbol{3}}_{i}\left( t \right)-2\cdot r_{i}\cdot{\boldsymbol{G}\boldsymbol{1}\boldsymbol{G}\boldsymbol{3}}_{i}\left( t \right)$$

$$\frac{d{\boldsymbol{G}\boldsymbol{1}\boldsymbol{G}\boldsymbol{4}}_{i}\left( t \right)}{dt}={c_{\boldsymbol{41}}\cdot\lambda_{{\boldsymbol{G}\boldsymbol{4}}_{i}}\left( t \right)\cdot{\boldsymbol{G}\boldsymbol{1}}_{i}\left( t \right)+c}_{\boldsymbol{14}}\cdot\lambda_{{\boldsymbol{G}\boldsymbol{1}}_{i}}\left( t \right)\cdot{\boldsymbol{G}\boldsymbol{4}}_{i}\left( t \right)-2\cdot r_{i}\cdot{\boldsymbol{G}\boldsymbol{1}\boldsymbol{G}\boldsymbol{4}}_{i}\left( t \right)$$

$$\frac{d{\boldsymbol{G}\boldsymbol{1}\boldsymbol{G}\boldsymbol{5}}_{i}\left( t \right)}{dt}={c_{\boldsymbol{51}}\cdot\lambda_{{\boldsymbol{G}\boldsymbol{5}}_{i}}\left( t \right)\cdot{\boldsymbol{G}\boldsymbol{1}}_{i}\left( t \right)+c}_{\boldsymbol{15}}\cdot\lambda_{{\boldsymbol{G}\boldsymbol{1}}_{i}}\left( t \right)\cdot{\boldsymbol{G}\boldsymbol{5}}_{i}\left( t \right)-2\cdot r_{i}\cdot{\boldsymbol{G1}\boldsymbol{G}\boldsymbol{5}}_{i}\left( t \right)$$

$$\frac{d{\boldsymbol{G}\boldsymbol{1}\boldsymbol{G}\boldsymbol{6}}_{i}\left( t \right)}{dt}={c_{\boldsymbol{61}}\cdot\lambda_{{\boldsymbol{G}\boldsymbol{6}}_{i}}\left( t \right)\cdot{\boldsymbol{G}\boldsymbol{1}}_{i}\left( t \right)+c}_{\boldsymbol{16}}\cdot\lambda_{{\boldsymbol{G}\boldsymbol{1}}_{i}}\left( t \right)\cdot{\boldsymbol{G}\boldsymbol{6}}_{i}\left( t \right)-2\cdot r_{i}\cdot{\boldsymbol{G}\boldsymbol{1}\boldsymbol{G}\boldsymbol{6}}_{i}\left( t \right)$$

$$\frac{d{\boldsymbol{G}\boldsymbol{1}\boldsymbol{G}\boldsymbol{7}}_{i}\left( t \right)}{dt}={c_{\boldsymbol{71}}\cdot\lambda_{{\boldsymbol{G}\boldsymbol{7}}_{i}}\left( t \right)\cdot{\boldsymbol{G}\boldsymbol{1}}_{i}\left( t \right)+c}_{\boldsymbol{17}}\cdot\lambda_{{\boldsymbol{G}\boldsymbol{1}}_{i}}\left( t \right)\cdot{\boldsymbol{G}\boldsymbol{7}}_{i}\left( t \right)-2\cdot r_{i}\cdot{\boldsymbol{G}\boldsymbol{1}\boldsymbol{G}\boldsymbol{7}}_{i}\left( t \right)$$

$$\frac{d{\boldsymbol{G}\boldsymbol{2}\boldsymbol{G}\boldsymbol{3}}_{i}(t)}{dt}={c_{\boldsymbol{32}}\cdot\lambda_{{\boldsymbol{G}\boldsymbol{3}}_{i}}\left( t \right)\cdot{\boldsymbol{G}\boldsymbol{2}}_{i}\left( t \right)+c}_{\boldsymbol{23}}\cdot\lambda_{{\boldsymbol{G}\boldsymbol{2}}_{i}}\left( t \right)\cdot{\boldsymbol{G}\boldsymbol{3}}_{i}\left( t \right)-2\cdot r_{i}\cdot{\boldsymbol{G}\boldsymbol{2}\boldsymbol{G}\boldsymbol{3}}_{i}(t)$$

$$\frac{d{\boldsymbol{G}\boldsymbol{2}\boldsymbol{G}\boldsymbol{4}}_{i}(t)}{dt}={c_{\boldsymbol{42}}\cdot\lambda_{{\boldsymbol{G}\boldsymbol{4}}_{i}}\left( t \right)\cdot{\boldsymbol{G}\boldsymbol{2}}_{i}\left( t \right)+c}_{\boldsymbol{24}}\cdot\lambda_{{\boldsymbol{G}\boldsymbol{2}}_{i}}\left( t \right)\cdot{\boldsymbol{G}\boldsymbol{4}}_{i}\left( t \right)-2\cdot r_{i}\cdot{\boldsymbol{G}\boldsymbol{2}\boldsymbol{G}\boldsymbol{4}}_{i}(t)$$

$$\frac{d{\boldsymbol{G}\boldsymbol{2}\boldsymbol{G}\boldsymbol{5}}_{i}(t)}{dt}={c_{\boldsymbol{52}}\cdot\lambda_{{\boldsymbol{G}\boldsymbol{5}}_{i}}\left( t \right)\cdot{\boldsymbol{G}\boldsymbol{2}}_{i}\left( t \right)+c}_{\boldsymbol{25}}\cdot\lambda_{{\boldsymbol{G}\boldsymbol{2}}_{i}}\left( t \right)\cdot{\boldsymbol{G}\boldsymbol{5}}_{i}\left( t \right)-2\cdot r_{i}\cdot{\boldsymbol{G}\boldsymbol{2}\boldsymbol{G}\boldsymbol{5}}_{i}(t)$$

$$\frac{d{\boldsymbol{G}\boldsymbol{2}\boldsymbol{G}\boldsymbol{6}}_{i}(t)}{dt}={c_{\boldsymbol{62}}\cdot\lambda_{{\boldsymbol{G}\boldsymbol{6}}_{i}}\left( t \right)\cdot{\boldsymbol{G}\boldsymbol{2}}_{i}\left( t \right)+c}_{\boldsymbol{26}}\cdot\lambda_{{\boldsymbol{G}\boldsymbol{2}}_{i}}\left( t \right)\cdot{\boldsymbol{G}\boldsymbol{6}}_{i}\left( t \right)-2\cdot r_{i}\cdot{\boldsymbol{G}\boldsymbol{2}\boldsymbol{G}\boldsymbol{6}}_{i}(t)$$

$$\frac{d{\boldsymbol{G}\boldsymbol{2}\boldsymbol{G}\boldsymbol{7}}_{i}(t)}{dt}={c_{\boldsymbol{72}}\cdot\lambda_{{\boldsymbol{G}\boldsymbol{7}}_{i}}\left( t \right)\cdot{\boldsymbol{G}\boldsymbol{2}}_{i}\left( t \right)+c}_{\boldsymbol{27}}\cdot\lambda_{{\boldsymbol{G}\boldsymbol{2}}_{i}}\left( t \right)\cdot{\boldsymbol{G}\boldsymbol{7}}_{i}\left( t \right)-2\cdot r_{i}\cdot{\boldsymbol{G}\boldsymbol{2}\boldsymbol{G}\boldsymbol{7}}_{i}(t)$$

$$\frac{d{\boldsymbol{G}\boldsymbol{3}\boldsymbol{G}\boldsymbol{4}}_{i}(t)}{dt}={c_{\boldsymbol{43}}\cdot\lambda_{{\boldsymbol{G}\boldsymbol{4}}_{i}}\left( t \right)\cdot{\boldsymbol{G}\boldsymbol{3}}_{i}\left( t \right)+c}_{\boldsymbol{34}}\cdot\lambda_{{\boldsymbol{G}\boldsymbol{3}}_{i}}\left( t \right)\cdot{\boldsymbol{G}\boldsymbol{4}}_{i}\left( t \right)-2\cdot r_{i}\cdot{\boldsymbol{G}\boldsymbol{3}\boldsymbol{G}\boldsymbol{4}}_{i}(t)$$

$$\frac{d{\boldsymbol{G}\boldsymbol{3}\boldsymbol{G}\boldsymbol{5}}_{i}(t)}{dt}={c_{\boldsymbol{53}}\cdot\lambda_{{\boldsymbol{G}\boldsymbol{5}}_{i}}\left( t \right)\cdot{\boldsymbol{G}\boldsymbol{3}}_{i}\left( t \right)+c}_{\boldsymbol{35}}\cdot\lambda_{{\boldsymbol{G}\boldsymbol{3}}_{i}}\left( t \right)\cdot{\boldsymbol{G}\boldsymbol{5}}_{i}\left( t \right)-2\cdot r_{i}\cdot{\boldsymbol{G}\boldsymbol{3}\boldsymbol{G}\boldsymbol{5}}_{i}(t)$$

$$\frac{d{\boldsymbol{G}\boldsymbol{3}\boldsymbol{G}\boldsymbol{6}}_{i}(t)}{dt}={c_{\boldsymbol{63}}\cdot\lambda_{{\boldsymbol{G}\boldsymbol{6}}_{i}}\left( t \right)\cdot{\boldsymbol{G}\boldsymbol{3}}_{i}\left( t \right)+c}_{\boldsymbol{36}}\cdot\lambda_{{\boldsymbol{G}\boldsymbol{3}}_{i}}\left( t \right)\cdot{\boldsymbol{G}\boldsymbol{6}}_{i}\left( t \right)-2\cdot r_{i}\cdot{\boldsymbol{G}\boldsymbol{3}\boldsymbol{G}\boldsymbol{6}}_{i}(t)$$

$$\frac{d{\boldsymbol{G}\boldsymbol{3}\boldsymbol{G}\boldsymbol{7}}_{i}(t)}{dt}={c_{\boldsymbol{73}}\cdot\lambda_{{\boldsymbol{G}\boldsymbol{7}}_{i}}\left( t \right)\cdot{\boldsymbol{G}\boldsymbol{3}}_{i}\left( t \right)+c}_{\boldsymbol{37}}\cdot\lambda_{{\boldsymbol{G}\boldsymbol{3}}_{i}}\left( t \right)\cdot{\boldsymbol{G}\boldsymbol{7}}_{i}\left( t \right)-2\cdot r_{i}\cdot{\boldsymbol{G}\boldsymbol{3}\boldsymbol{G}\boldsymbol{7}}_{i}(t)$$

$$\frac{d{\boldsymbol{G}\boldsymbol{4}\boldsymbol{G}\boldsymbol{5}}_{i}(t)}{dt}={c_{\boldsymbol{54}}\cdot\lambda_{{\boldsymbol{G}\boldsymbol{5}}_{i}}\left( t \right)\cdot{\boldsymbol{G}\boldsymbol{4}}_{i}\left( t \right)+c}_{\boldsymbol{45}}\cdot\lambda_{{\boldsymbol{G}\boldsymbol{4}}_{i}}\left( t \right)\cdot{\boldsymbol{G}\boldsymbol{5}}_{i}\left( t \right)-2\cdot r_{i}\cdot{\boldsymbol{G}\boldsymbol{4}\boldsymbol{G}\boldsymbol{5}}_{i}(t)$$

$$\frac{d{\boldsymbol{G}\boldsymbol{4}\boldsymbol{G}\boldsymbol{6}}_{i}(t)}{dt}={c_{\boldsymbol{64}}\cdot\lambda_{{\boldsymbol{G}\boldsymbol{6}}_{i}}\left( t \right)\cdot{\boldsymbol{G}\boldsymbol{4}}_{i}\left( t \right)+c}_{\boldsymbol{46}}\cdot\lambda_{{\boldsymbol{G}\boldsymbol{4}}_{i}}\left( t \right)\cdot{\boldsymbol{G}\boldsymbol{6}}_{i}\left( t \right)-2\cdot r_{i}\cdot{\boldsymbol{G}\boldsymbol{4}\boldsymbol{G}\boldsymbol{6}}_{i}(t)$$

$$\frac{d{\boldsymbol{G}\boldsymbol{4}\boldsymbol{G}\boldsymbol{7}}_{i}(t)}{dt}={c_{\boldsymbol{74}}\cdot\lambda_{{\boldsymbol{G}\boldsymbol{7}}_{i}}\left( t \right)\cdot{\boldsymbol{G}\boldsymbol{4}}_{i}\left( t \right)+c}_{\boldsymbol{47}}\cdot\lambda_{{\boldsymbol{G}\boldsymbol{4}}_{i}}\left( t \right)\cdot{\boldsymbol{G}\boldsymbol{7}}_{i}\left( t \right)-2\cdot r_{i}\cdot{\boldsymbol{G}\boldsymbol{4}\boldsymbol{G}\boldsymbol{7}}_{i}(t)$$

$$\frac{d{\boldsymbol{G}\boldsymbol{5}\boldsymbol{G}\boldsymbol{6}}_{i}(t)}{dt}={c_{\boldsymbol{65}}\cdot\lambda_{{\boldsymbol{G}\boldsymbol{6}}_{i}}\left( t \right)\cdot{\boldsymbol{G}\boldsymbol{5}}_{i}\left( t \right)+c}_{\boldsymbol{56}}\cdot\lambda_{{\boldsymbol{G}\boldsymbol{5}}_{i}}\left( t \right)\cdot{\boldsymbol{G}\boldsymbol{6}}_{i}\left( t \right)-2\cdot r_{i}\cdot{\boldsymbol{G}\boldsymbol{5}\boldsymbol{G}\boldsymbol{6}}_{i}(t)$$

$$\frac{d{\boldsymbol{G}\boldsymbol{5}\boldsymbol{G}\boldsymbol{7}}_{i}(t)}{dt}={c_{\boldsymbol{75}}\cdot\lambda_{{\boldsymbol{G}\boldsymbol{7}}_{i}}\left( t \right)\cdot{\boldsymbol{G}\boldsymbol{5}}_{i}\left( t \right)+c}_{\boldsymbol{57}}\cdot\lambda_{{\boldsymbol{G}\boldsymbol{5}}_{i}}\left( t \right)\cdot{\boldsymbol{G}\boldsymbol{7}}_{i}\left( t \right)-2\cdot r_{i}\cdot{\boldsymbol{G}\boldsymbol{5}\boldsymbol{G}\boldsymbol{7}}_{i}(t)$$

$$\frac{d{\boldsymbol{G}\boldsymbol{6}\boldsymbol{G}\boldsymbol{7}}_{i}(t)}{dt}={c_{\boldsymbol{76}}\cdot\lambda_{{\boldsymbol{G}\boldsymbol{7}}_{i}}\left( t \right)\cdot{\boldsymbol{G}\boldsymbol{6}}_{i}\left( t \right)+c}_{\boldsymbol{67}}\cdot\lambda_{{\boldsymbol{G}\boldsymbol{6}}_{i}}\left( t \right)\cdot{\boldsymbol{G}\boldsymbol{7}}_{i}\left( t \right)-2\cdot r_{i}\cdot{\boldsymbol{G}\boldsymbol{6}\boldsymbol{G}\boldsymbol{7}}_{i}(t)$$

with transmission risks as follows:

$$\lambda_{{\boldsymbol{G}\boldsymbol{1}}_{i}}\left( t \right)=\alpha_{{\boldsymbol{G}\boldsymbol{1}}_{i}}\cdot\sum_{j=1}^{m} \beta_{ij}\cdot\left( {\boldsymbol{G}\boldsymbol{1}}_{j}\left( t \right)+{\boldsymbol{G}\boldsymbol{1}\boldsymbol{G}\boldsymbol{2}}_{j}\left( t \right)+{\boldsymbol{G}\boldsymbol{1}\boldsymbol{G}\boldsymbol{3}}_{j}\left( t \right)+{\boldsymbol{G}\boldsymbol{1}\boldsymbol{G}\boldsymbol{4}}_{j}\left( t \right)+{\boldsymbol{G}\boldsymbol{1}\boldsymbol{G}\boldsymbol{5}}_{j}(t)+{\boldsymbol{G}\boldsymbol{1}\boldsymbol{G}\boldsymbol{6}}_{j}(t)+{\boldsymbol{G}\boldsymbol{1}\boldsymbol{G}\boldsymbol{7}}_{j}(t) \right)$$

$$\lambda_{{\boldsymbol{G}\boldsymbol{2}}_{i}}\left( t \right)=\alpha_{{\boldsymbol{G}\boldsymbol{2}}_{i}}\cdot\sum_{j=1}^{m} \beta_{ij}\cdot\left( {\boldsymbol{G}\boldsymbol{2}}_{j}\left( t \right)+{\boldsymbol{G}\boldsymbol{1}\boldsymbol{G}\boldsymbol{2}}_{j}\left( t \right)+{\boldsymbol{G}\boldsymbol{2}\boldsymbol{G}\boldsymbol{3}}_{j}\left( t \right)+{\boldsymbol{G}\boldsymbol{2}\boldsymbol{G}\boldsymbol{4}}_{j}\left( t \right)+{\boldsymbol{G}\boldsymbol{2}\boldsymbol{G}\boldsymbol{5}}_{j}(t)+{\boldsymbol{G}\boldsymbol{2}\boldsymbol{G}\boldsymbol{6}}_{j}(t)+{\boldsymbol{G}\boldsymbol{2}\boldsymbol{G}\boldsymbol{7}}_{j}(t) \right)$$

$$\lambda_{{\boldsymbol{G}\boldsymbol{3}}_{i}}\left( t \right)=\alpha_{{\boldsymbol{G}\boldsymbol{3}}_{i}}\cdot\sum_{j=1}^{m} \beta_{ij}\cdot\left( {\boldsymbol{G}\boldsymbol{3}}_{j}\left( t \right)+{\boldsymbol{G}\boldsymbol{1}\boldsymbol{G}\boldsymbol{3}}_{j}\left( t \right)+{\boldsymbol{G}\boldsymbol{2}\boldsymbol{G}\boldsymbol{3}}_{j}\left( t \right)+{\boldsymbol{G}\boldsymbol{3}\boldsymbol{G}\boldsymbol{4}}_{j}\left( t \right)+{\boldsymbol{G}\boldsymbol{3}\boldsymbol{G}\boldsymbol{5}}_{j}(t)+{\boldsymbol{G}\boldsymbol{3}\boldsymbol{G}\boldsymbol{6}}_{j}(t)+{\boldsymbol{G}\boldsymbol{3}\boldsymbol{G}\boldsymbol{7}}_{j}(t) \right)$$

$$\lambda_{{\boldsymbol{G}\boldsymbol{4}}_{i}}\left( t \right)=\alpha_{{\boldsymbol{G}\boldsymbol{4}}_{i}}\cdot\sum_{j=1}^{m} \beta_{ij}\cdot\left( {\boldsymbol{G}\boldsymbol{4}}_{j}\left( t \right)+{\boldsymbol{G}\boldsymbol{1}\boldsymbol{G}\boldsymbol{4}}_{j}\left( t \right)+{\boldsymbol{G}\boldsymbol{2}\boldsymbol{G}\boldsymbol{4}}_{j}\left( t \right)+{\boldsymbol{G}\boldsymbol{3}\boldsymbol{G}\boldsymbol{4}}_{j}\left( t \right)+{\boldsymbol{G}\boldsymbol{4}\boldsymbol{G}\boldsymbol{5}}_{j}(t)+{\boldsymbol{G}\boldsymbol{4}\boldsymbol{G}\boldsymbol{6}}_{j}(t)+{\boldsymbol{G}\boldsymbol{4}\boldsymbol{G}\boldsymbol{7}}_{j}(t) \right)$$

$$\lambda_{{\boldsymbol{G}\boldsymbol{5}}_{i}}\left( t \right)=\alpha_{{\boldsymbol{G}\boldsymbol{5}}_{i}}\cdot\sum_{j=1}^{m} \beta_{ij}\cdot\left( {\boldsymbol{G}\boldsymbol{5}}_{j}\left( t \right)+{\boldsymbol{G}\boldsymbol{1}\boldsymbol{G}\boldsymbol{5}}_{j}\left( t \right)+{\boldsymbol{G}\boldsymbol{2}\boldsymbol{G}\boldsymbol{5}}_{j}\left( t \right)+{\boldsymbol{G}\boldsymbol{3}\boldsymbol{G}\boldsymbol{5}}_{j}\left( t \right)+{\boldsymbol{G}\boldsymbol{4}\boldsymbol{G}\boldsymbol{5}}_{j}(t)+{\boldsymbol{G}\boldsymbol{5}\boldsymbol{G}\boldsymbol{6}}_{j}(t)+{\boldsymbol{G}\boldsymbol{5}\boldsymbol{G}\boldsymbol{7}}_{j}(t) \right)$$

$$\lambda_{{\boldsymbol{G}\boldsymbol{6}}_{i}}\left( t \right)=\alpha_{{\boldsymbol{G}\boldsymbol{6}}_{i}}\cdot\sum_{j=1}^{m} \beta_{ij}\cdot\left( {\boldsymbol{G}\boldsymbol{6}}_{j}\left( t \right)+{\boldsymbol{G}\boldsymbol{1}\boldsymbol{G}\boldsymbol{6}}_{j}\left( t \right)+{\boldsymbol{G}\boldsymbol{2}\boldsymbol{G}\boldsymbol{6}}_{j}\left( t \right)+{\boldsymbol{G}\boldsymbol{3}\boldsymbol{G}\boldsymbol{6}}_{j}\left( t \right)+{\boldsymbol{G}\boldsymbol{4}\boldsymbol{G}\boldsymbol{6}}_{j}(t)+{\boldsymbol{G}\boldsymbol{5}\boldsymbol{G}\boldsymbol{6}}_{j}(t)+{\boldsymbol{G}\boldsymbol{6}\boldsymbol{G}\boldsymbol{7}}_{j}(t) \right)$$

$$\lambda_{{\boldsymbol{G}\boldsymbol{7}}_{i}}\left( t \right)=\alpha_{{\boldsymbol{G}\boldsymbol{7}}_{i}}\cdot\sum_{j=1}^{m} \beta_{ij}\cdot\left( {\boldsymbol{G}\boldsymbol{7}}_{j}\left( t \right)+{\boldsymbol{G}\boldsymbol{1}\boldsymbol{G}\boldsymbol{7}}_{j}\left( t \right)+{\boldsymbol{G}\boldsymbol{2}\boldsymbol{G}\boldsymbol{7}}_{j}\left( t \right)+{\boldsymbol{G}\boldsymbol{3}\boldsymbol{G}\boldsymbol{7}}_{j}\left( t \right)+{\boldsymbol{G}\boldsymbol{4}\boldsymbol{G}\boldsymbol{7}}_{j}(t)+{\boldsymbol{G}\boldsymbol{5}\boldsymbol{G}\boldsymbol{7}}_{j}(t)+{\boldsymbol{G}\boldsymbol{6}\boldsymbol{G}\boldsymbol{7}}_{j}(t) \right)$$

and the following condition ($n=7$ serotype groups):

$$\boldsymbol{S}_{i}\left( t \right)+\sum_{k=1}^{n} \boldsymbol{Gk}_{i}\left( t \right)+\sum_{k=1}^{n-1} \sum_{l=k+1}^{n} \boldsymbol{GkGl}_{i}\left( t \right)=1 \text{∀}i=1,\ldots,m$$

**3. Model equations (vaccination model)**

**7 serotype groups (with vaccines** $\boldsymbol{v}\boldsymbol{1}$ **[PCV7] and** $\boldsymbol{v}\boldsymbol{2}$ **[PCV13])**, $i=1,\ldots,m$ for $m=400$ age strata (3 months each)

87 compartments (non-carriers, 7 single carrier, and 21 double carrier compartments for 3 vaccination states, respectively), 34.800 equations total

$$\frac{d\boldsymbol{S}_{i}(t)}{dt}=r_{i}\cdot\left( {\boldsymbol{G}\boldsymbol{1}}_{i}\left( t \right)+{\boldsymbol{G}\boldsymbol{2}}_{i}\left( t \right)+{\boldsymbol{G}\boldsymbol{3}}_{i}\left( t \right)+{\boldsymbol{G}\boldsymbol{4}}_{i}\left( t \right)+{\boldsymbol{G}\boldsymbol{5}}_{i}\left( t \right)+{\boldsymbol{G}\boldsymbol{6}}_{i}\left( t \right)+{\boldsymbol{G}\boldsymbol{7}}_{i}\left( t \right) \right)-\left( \lambda_{{\boldsymbol{G}\boldsymbol{1}}_{i}}\left( t \right)+\lambda_{{\boldsymbol{G}\boldsymbol{2}}_{i}}\left( t \right)+\lambda_{{\boldsymbol{G}\boldsymbol{3}}_{i}}\left( t \right)+\lambda_{{\boldsymbol{G}\boldsymbol{4}}_{i}}\left( t \right)+\lambda_{{\boldsymbol{G}\boldsymbol{5}}_{i}}\left( t \right)+\lambda_{{\boldsymbol{G}\boldsymbol{6}}_{i}}\left( t \right)+\lambda_{{\boldsymbol{G}\boldsymbol{7}}_{i}}\left( t \right)+\pi_{{v1}_{i}}\left( t \right)+\pi_{{v2}_{i}}\left( t \right) \right)\cdot\boldsymbol{S}_{i}\left( t \right)+\omega_{{v1}_{i}}\cdot{\boldsymbol{Sv}\boldsymbol{1}}_{i}\left( t \right)+\omega_{{v2}_{i}}\cdot{\boldsymbol{Sv}\boldsymbol{2}}_{i}\left( t \right)$$

$$\frac{d{\boldsymbol{Sv}\boldsymbol{1}}_{i}(t)}{dt}=r_{i}\cdot\left( {\boldsymbol{G}\boldsymbol{1}\boldsymbol{v}\boldsymbol{1}}_{i}\left( t \right)+{\boldsymbol{G}\boldsymbol{2}\boldsymbol{v}\boldsymbol{1}}_{i}\left( t \right)+{\boldsymbol{G}\boldsymbol{3}\boldsymbol{v}\boldsymbol{1}}_{i}\left( t \right)+{\boldsymbol{G}\boldsymbol{4}\boldsymbol{v}\boldsymbol{1}}_{i}\left( t \right)+{\boldsymbol{G5}\boldsymbol{v}\boldsymbol{1}}_{i}\left( t \right)+{\boldsymbol{G}\boldsymbol{6}\boldsymbol{v}\boldsymbol{1}}_{i}\left( t \right)+{\boldsymbol{G}\boldsymbol{7}\boldsymbol{v}\boldsymbol{1}}_{i}\left( t \right) \right)-\left( \left( 1-{v1}_{\boldsymbol{G}\boldsymbol{1}} \right)\cdot\lambda_{{\boldsymbol{G}\boldsymbol{1}}_{i}}\left( t \right)+\lambda_{{\boldsymbol{G}\boldsymbol{2}}_{i}}\left( t \right)+\lambda_{{\boldsymbol{G}\boldsymbol{3}}_{i}}\left( t \right)+\lambda_{{\boldsymbol{G}\boldsymbol{4}}_{i}}\left( t \right)+\lambda_{{\boldsymbol{G}\boldsymbol{5}}_{i}}\left( t \right)+\lambda_{{\boldsymbol{G}\boldsymbol{6}}_{i}}\left( t \right)+\lambda_{{\boldsymbol{G}\boldsymbol{7}}_{i}}\left( t \right)+\omega_{{v1}_{i}} \right)\cdot{\boldsymbol{Sv}\boldsymbol{1}}_{i}\left( t \right)+\pi_{{v1}_{i}}(t)\cdot\boldsymbol{S}_{i}(t)$$

$$\frac{d{\boldsymbol{Sv}\boldsymbol{2}}_{i}(t)}{dt}=r_{i}\cdot\left( {\boldsymbol{G}\boldsymbol{1}\boldsymbol{v}\boldsymbol{2}}_{i}\left( t \right)+{\boldsymbol{G}\boldsymbol{2}\boldsymbol{v}\boldsymbol{2}}_{i}\left( t \right)+{\boldsymbol{G}\boldsymbol{3}\boldsymbol{v}\boldsymbol{2}}_{i}\left( t \right)+{\boldsymbol{G}\boldsymbol{4}\boldsymbol{v}\boldsymbol{2}}_{i}\left( t \right)+{\boldsymbol{G}\boldsymbol{5}\boldsymbol{v}\boldsymbol{2}}_{i}\left( t \right)+{\boldsymbol{G}\boldsymbol{6}\boldsymbol{v}\boldsymbol{2}}_{i}\left( t \right)+{\boldsymbol{G}\boldsymbol{7}\boldsymbol{v}\boldsymbol{2}}_{i}\left( t \right) \right)-\left( \left( 1-{v2}_{\boldsymbol{G}\boldsymbol{1}} \right)\cdot\lambda_{{\boldsymbol{G}\boldsymbol{1}}_{i}}\left( t \right)+(1-{v2}_{\boldsymbol{G}\boldsymbol{2}})\cdot\lambda_{{\boldsymbol{G}\boldsymbol{2}}_{i}}\left( t \right)+{(1-{v2}_{\boldsymbol{G}\boldsymbol{3}})\cdot\lambda}_{{\boldsymbol{G}\boldsymbol{3}}_{i}}\left( t \right)+\lambda_{{\boldsymbol{G}\boldsymbol{4}}_{i}}\left( t \right)+\lambda_{{\boldsymbol{G}\boldsymbol{5}}_{i}}\left( t \right)+\lambda_{{\boldsymbol{G}\boldsymbol{6}}_{i}}\left( t \right)+\lambda_{{\boldsymbol{G}\boldsymbol{7}}_{i}}\left( t \right)+\omega_{{v2}_{i}} \right)\cdot{\boldsymbol{Sv}\boldsymbol{2}}_{i}\left( t \right)+\pi_{{v2}_{i}}(t)\cdot\boldsymbol{S}_{i}(t)$$

$$\frac{d{\boldsymbol{G}\boldsymbol{1}}_{i}(t)}{dt}=\lambda_{{\boldsymbol{G}\boldsymbol{1}}_{i}}\left( t \right)\cdot\boldsymbol{S}_{i}\left( t \right)+r_{i}\cdot\left( {\boldsymbol{G}\boldsymbol{1}\boldsymbol{G}\boldsymbol{2}}_{i}\left( t \right)+{\boldsymbol{G}\boldsymbol{1}\boldsymbol{G}\boldsymbol{3}}_{i}\left( t \right)+{\boldsymbol{G}\boldsymbol{1}\boldsymbol{G}\boldsymbol{4}}_{i}\left( t \right)+{\boldsymbol{G}\boldsymbol{1}\boldsymbol{G}\boldsymbol{5}}_{i}\left( t \right)+{\boldsymbol{G}\boldsymbol{1}\boldsymbol{G}\boldsymbol{6}}_{i}\left( t \right)+{\boldsymbol{G}\boldsymbol{1}\boldsymbol{G}\boldsymbol{7}}_{i}\left( t \right) \right)-\left( c_{\boldsymbol{21}}\cdot\lambda_{{\boldsymbol{G}\boldsymbol{2}}_{i}}\left( t \right)+c_{\boldsymbol{31}}\cdot\lambda_{{\boldsymbol{G}\boldsymbol{3}}_{i}}\left( t \right)+c_{\boldsymbol{41}}\cdot\lambda_{{\boldsymbol{G}\boldsymbol{4}}_{i}}\left( t \right)+c_{\boldsymbol{51}}\cdot\lambda_{{\boldsymbol{G}\boldsymbol{5}}_{i}}\left( t \right)+c_{\boldsymbol{61}}\cdot\lambda_{{\boldsymbol{G}\boldsymbol{6}}_{i}}\left( t \right)+c_{\boldsymbol{71}}\cdot\lambda_{{\boldsymbol{G}\boldsymbol{7}}_{i}}\left( t \right)+r_{i}+\pi_{{v1}_{i}}\left( t \right)+\pi_{{v2}_{i}}\left( t \right) \right)\cdot{\boldsymbol{G}\boldsymbol{1}}_{i}\left( t \right)+\omega_{{v1}_{i}}\cdot{\boldsymbol{G}\boldsymbol{1}\boldsymbol{v}\boldsymbol{1}}_{i}\left( t \right)+\omega_{{v2}_{i}}\cdot{\boldsymbol{G}\boldsymbol{1}\boldsymbol{v}\boldsymbol{2}}_{i}\left( t \right)$$

$$\frac{d{\boldsymbol{G}\boldsymbol{1}\boldsymbol{v}\boldsymbol{1}}_{i}(t)}{dt}=(1-{v1}_{\boldsymbol{G}\boldsymbol{1}})\cdot\lambda_{{\boldsymbol{G}\boldsymbol{1}}_{i}}\left( t \right)\cdot{\boldsymbol{Sv}\boldsymbol{1}}_{i}\left( t \right)+r_{i}\cdot\left( {\boldsymbol{G}\boldsymbol{1}\boldsymbol{G}\boldsymbol{2}\boldsymbol{v}\boldsymbol{1}}_{i}\left( t \right)+{\boldsymbol{G}\boldsymbol{1}\boldsymbol{G}\boldsymbol{3}\boldsymbol{v}\boldsymbol{1}}_{i}\left( t \right)+{\boldsymbol{G}\boldsymbol{1}\boldsymbol{G}\boldsymbol{4}\boldsymbol{v}\boldsymbol{1}}_{i}\left( t \right)+{\boldsymbol{G}\boldsymbol{1}\boldsymbol{G}\boldsymbol{5}\boldsymbol{v}\boldsymbol{1}}_{i}\left( t \right)+{\boldsymbol{G}\boldsymbol{1}\boldsymbol{G}\boldsymbol{6}\boldsymbol{v}\boldsymbol{1}}_{i}\left( t \right)+{\boldsymbol{G}\boldsymbol{1}\boldsymbol{G}\boldsymbol{7}\boldsymbol{v}\boldsymbol{1}}_{i}\left( t \right) \right)-\left( c_{\boldsymbol{21}}\cdot\lambda_{{\boldsymbol{G}\boldsymbol{2}}_{i}}\left( t \right)+c_{\boldsymbol{31}}\cdot\lambda_{{\boldsymbol{G}\boldsymbol{3}}_{i}}\left( t \right)+c_{\boldsymbol{41}}\cdot\lambda_{{\boldsymbol{G}\boldsymbol{4}}_{i}}\left( t \right)+c_{\boldsymbol{51}}\cdot\lambda_{{\boldsymbol{G}\boldsymbol{5}}_{i}}\left( t \right)+c_{\boldsymbol{61}}\cdot\lambda_{{\boldsymbol{G}\boldsymbol{6}}_{i}}\left( t \right)+c_{\boldsymbol{71}}\cdot\lambda_{{\boldsymbol{G}\boldsymbol{7}}_{i}}\left( t \right)+r_{i}+\omega_{{v1}_{i}} \right)\cdot{\boldsymbol{G}\boldsymbol{1v}\boldsymbol{1}}_{i}\left( t \right)+\pi_{{v1}_{i}}(t)\cdot{\boldsymbol{G}\boldsymbol{1}}_{i}(t)$$

$$\frac{d{\boldsymbol{G}\boldsymbol{1}\boldsymbol{v}\boldsymbol{2}}_{i}(t)}{dt}=(1-{v2}_{\boldsymbol{G}\boldsymbol{1}})\cdot\lambda_{{\boldsymbol{G}\boldsymbol{1}}_{i}}\left( t \right)\cdot{\boldsymbol{Sv}\boldsymbol{2}}_{i}\left( t \right)+r_{i}\cdot\left( {\boldsymbol{G}\boldsymbol{1}\boldsymbol{G}\boldsymbol{2}\boldsymbol{v}\boldsymbol{2}}_{i}\left( t \right)+{\boldsymbol{G}\boldsymbol{1}\boldsymbol{G}\boldsymbol{3}\boldsymbol{v}\boldsymbol{2}}_{i}\left( t \right)+{\boldsymbol{G}\boldsymbol{1}\boldsymbol{G}\boldsymbol{4}\boldsymbol{v}\boldsymbol{2}}_{i}\left( t \right)+{\boldsymbol{G}\boldsymbol{1}\boldsymbol{G}\boldsymbol{5}\boldsymbol{v}\boldsymbol{2}}_{i}\left( t \right)+{\boldsymbol{G}\boldsymbol{1}\boldsymbol{G}\boldsymbol{6}\boldsymbol{v}\boldsymbol{2}}_{i}\left( t \right)+{\boldsymbol{G}\boldsymbol{1}\boldsymbol{G}\boldsymbol{7}\boldsymbol{v}\boldsymbol{2}}_{i}\left( t \right) \right)-\left( c_{\boldsymbol{21}}\cdot{\left( 1-{v2}_{\boldsymbol{G}\boldsymbol{2}} \right)\cdot\lambda}_{{\boldsymbol{G}\boldsymbol{2}}_{i}}\left( t \right)+c_{\boldsymbol{31}}\cdot(1-{v2}_{\boldsymbol{G}\boldsymbol{3}})\cdot\lambda_{{\boldsymbol{G}\boldsymbol{3}}_{i}}\left( t \right)+c_{\boldsymbol{41}}\cdot\lambda_{{\boldsymbol{G}\boldsymbol{4}}_{i}}\left( t \right)+c_{\boldsymbol{51}}\cdot\lambda_{{\boldsymbol{G}\boldsymbol{5}}_{i}}\left( t \right)+c_{\boldsymbol{61}}\cdot\lambda_{{\boldsymbol{G}\boldsymbol{6}}_{i}}\left( t \right)+c_{\boldsymbol{71}}\cdot\lambda_{{\boldsymbol{G}\boldsymbol{7}}_{i}}\left( t \right)+r_{i}+\omega_{{v2}_{i}} \right)\cdot{\boldsymbol{G}\boldsymbol{1}\boldsymbol{v}\boldsymbol{2}}_{i}\left( t \right)+\pi_{{v2}_{i}}(t)\cdot{\boldsymbol{G}\boldsymbol{1}}_{i}(t)$$

$$\frac{d{\boldsymbol{G}\boldsymbol{2}}_{i}(t)}{dt}=\lambda_{{\boldsymbol{G}\boldsymbol{2}}_{i}}\left( t \right)\cdot\boldsymbol{S}_{i}\left( t \right)+r_{i}\cdot\left( {\boldsymbol{G}\boldsymbol{1}\boldsymbol{G}\boldsymbol{2}}_{i}\left( t \right)+{\boldsymbol{G}\boldsymbol{2}\boldsymbol{G}\boldsymbol{3}}_{i}\left( t \right)+{\boldsymbol{G}\boldsymbol{2}\boldsymbol{G}\boldsymbol{4}}_{i}\left( t \right)+{\boldsymbol{G}\boldsymbol{2}\boldsymbol{G}\boldsymbol{5}}_{i}(t)+{\boldsymbol{G}\boldsymbol{2}\boldsymbol{G}\boldsymbol{6}}_{i}\left( t \right)+{\boldsymbol{G}\boldsymbol{2}\boldsymbol{G}\boldsymbol{7}}_{i}\left( t \right) \right)-\left( c_{\boldsymbol{12}}\cdot\lambda_{{\boldsymbol{G}\boldsymbol{1}}_{i}}\left( t \right)+c_{\boldsymbol{32}}\cdot\lambda_{{\boldsymbol{G}\boldsymbol{3}}_{i}}\left( t \right)+c_{\boldsymbol{42}}\cdot\lambda_{{\boldsymbol{G}\boldsymbol{4}}_{i}}\left( t \right)+c_{\boldsymbol{52}}\cdot\lambda_{{\boldsymbol{G}\boldsymbol{5}}_{i}}\left( t \right)+c_{\boldsymbol{62}}\cdot\lambda_{{\boldsymbol{G}\boldsymbol{6}}_{i}}\left( t \right)+c_{\boldsymbol{72}}\cdot\lambda_{{\boldsymbol{G}\boldsymbol{7}}_{i}}\left( t \right)+r_{i}+\pi_{{v1}_{i}}\left( t \right)+\pi_{{v2}_{i}}\left( t \right) \right)\cdot{\boldsymbol{G}\boldsymbol{2}}_{i}(t)+\omega_{{v1}_{i}}\cdot{\boldsymbol{G}\boldsymbol{2}\boldsymbol{v}\boldsymbol{1}}_{i}\left( t \right)+\omega_{{v2}_{i}}\cdot{\boldsymbol{G}\boldsymbol{2}\boldsymbol{v}\boldsymbol{2}}_{i}\left( t \right)$$

$$\frac{d{\boldsymbol{G}\boldsymbol{2}\boldsymbol{v}\boldsymbol{1}}_{i}(t)}{dt}=\lambda_{{\boldsymbol{G}\boldsymbol{2}}_{i}}\left( t \right)\cdot{\boldsymbol{Sv}\boldsymbol{1}}_{i}\left( t \right)+r_{i}\cdot\left( {\boldsymbol{G}\boldsymbol{1}\boldsymbol{G}\boldsymbol{2}\boldsymbol{v}\boldsymbol{1}}_{i}\left( t \right)+{\boldsymbol{G}\boldsymbol{2}\boldsymbol{G}\boldsymbol{3}\boldsymbol{v}\boldsymbol{1}}_{i}\left( t \right)+{\boldsymbol{G2G}\boldsymbol{4}\boldsymbol{v}\boldsymbol{1}}_{i}\left( t \right)+{\boldsymbol{G}\boldsymbol{2}\boldsymbol{G}\boldsymbol{5}\boldsymbol{v}\boldsymbol{1}}_{i}\left( t \right)+{\boldsymbol{G}\boldsymbol{2}\boldsymbol{G}\boldsymbol{6}\boldsymbol{v}\boldsymbol{1}}_{i}\left( t \right)+{\boldsymbol{G}\boldsymbol{2}\boldsymbol{G}\boldsymbol{7}\boldsymbol{v}\boldsymbol{1}}_{i}\left( t \right) \right)-\left( c_{\boldsymbol{12}}\cdot\left( 1-{v1}_{\boldsymbol{G}\boldsymbol{1}} \right)\cdot\lambda_{{\boldsymbol{G}\boldsymbol{1}}_{i}}\left( t \right)+c_{\boldsymbol{32}}\cdot\lambda_{{\boldsymbol{G}\boldsymbol{3}}_{i}}\left( t \right)+c_{\boldsymbol{42}}\cdot\lambda_{{\boldsymbol{G}\boldsymbol{4}}_{i}}\left( t \right)+c_{\boldsymbol{52}}\cdot\lambda_{{\boldsymbol{G}\boldsymbol{5}}_{i}}\left( t \right)+c_{\boldsymbol{62}}\cdot\lambda_{{\boldsymbol{G}\boldsymbol{6}}_{i}}\left( t \right)+c_{\boldsymbol{72}}\cdot\lambda_{{\boldsymbol{G}\boldsymbol{7}}_{i}}\left( t \right)+r_{i}+\omega_{{v1}_{i}} \right)\cdot{\boldsymbol{G}\boldsymbol{2}\boldsymbol{v}\boldsymbol{1}}_{i}\left( t \right)+\pi_{{v1}_{i}}(t)\cdot{\boldsymbol{G}\boldsymbol{2}}_{i}(t)$$

$$\frac{d{\boldsymbol{G}\boldsymbol{2}\boldsymbol{v}\boldsymbol{2}}_{i}(t)}{dt}={(1-{v2}_{\boldsymbol{G}\boldsymbol{2}})\cdot\lambda}_{{\boldsymbol{G}\boldsymbol{2}}_{i}}\left( t \right)\cdot{\boldsymbol{Sv}\boldsymbol{2}}_{i}\left( t \right)+r_{i}\cdot\left( {\boldsymbol{G}\boldsymbol{1}\boldsymbol{G}\boldsymbol{2}\boldsymbol{v}\boldsymbol{2}}_{i}\left( t \right)+{\boldsymbol{G}\boldsymbol{2}\boldsymbol{G}\boldsymbol{3}\boldsymbol{v}\boldsymbol{2}}_{i}\left( t \right)+{\boldsymbol{G}\boldsymbol{2}\boldsymbol{G}\boldsymbol{4}\boldsymbol{v}\boldsymbol{2}}_{i}\left( t \right)+{\boldsymbol{G}\boldsymbol{2}\boldsymbol{G}\boldsymbol{5}\boldsymbol{v}\boldsymbol{2}}_{i}\left( t \right)+{\boldsymbol{G}\boldsymbol{2}\boldsymbol{G}\boldsymbol{6}\boldsymbol{v}\boldsymbol{2}}_{i}\left( t \right)+{\boldsymbol{G}\boldsymbol{2}\boldsymbol{G}\boldsymbol{7}\boldsymbol{v}\boldsymbol{2}}_{i}\left( t \right) \right)-\left( c_{\boldsymbol{12}}\cdot\left( 1-{v2}_{\boldsymbol{G}\boldsymbol{1}} \right)\cdot\lambda_{{\boldsymbol{G}\boldsymbol{1}}_{i}}\left( t \right)+c_{\boldsymbol{32}}\cdot{(1-{v2}_{\boldsymbol{G}\boldsymbol{3}})\cdot\lambda}_{{\boldsymbol{G}\boldsymbol{3}}_{i}}\left( t \right)+c_{\boldsymbol{42}}\cdot\lambda_{{\boldsymbol{G}\boldsymbol{4}}_{i}}\left( t \right)+c_{\boldsymbol{52}}\cdot\lambda_{{\boldsymbol{G}\boldsymbol{5}}_{i}}\left( t \right)+c_{\boldsymbol{62}}\cdot\lambda_{{\boldsymbol{G}\boldsymbol{6}}_{i}}\left( t \right)+c_{\boldsymbol{72}}\cdot\lambda_{{\boldsymbol{G}\boldsymbol{7}}_{i}}\left( t \right)+r_{i}+\omega_{{v2}_{i}} \right)\cdot{\boldsymbol{G}\boldsymbol{2}\boldsymbol{v}\boldsymbol{2}}_{i}\left( t \right)+\pi_{{v2}_{i}}(t)\cdot{\boldsymbol{G}\boldsymbol{2}}_{i}(t)$$

$$\frac{d{\boldsymbol{G}\boldsymbol{3}}_{i}(t)}{dt}=\lambda_{{\boldsymbol{G}\boldsymbol{3}}_{i}}\left( t \right)\cdot\boldsymbol{S}_{i}\left( t \right)+r_{i}\cdot\left( {\boldsymbol{G}\boldsymbol{1}\boldsymbol{G}\boldsymbol{3}}_{i}\left( t \right)+{\boldsymbol{G}\boldsymbol{2}\boldsymbol{G}\boldsymbol{3}}_{i}\left( t \right)+{\boldsymbol{G}\boldsymbol{3}\boldsymbol{G}\boldsymbol{4}}_{i}\left( t \right)+{\boldsymbol{G}\boldsymbol{3}\boldsymbol{G}\boldsymbol{5}}_{i}\left( t \right)+{\boldsymbol{G}\boldsymbol{3}\boldsymbol{G}\boldsymbol{6}}_{i}\left( t \right)+{\boldsymbol{G}\boldsymbol{3}\boldsymbol{G}\boldsymbol{7}}_{i}\left( t \right) \right)-\left( c_{\boldsymbol{13}}\cdot\lambda_{{\boldsymbol{G}\boldsymbol{1}}_{i}}\left( t \right)+c_{\boldsymbol{23}}\cdot\lambda_{{\boldsymbol{G}\boldsymbol{2}}_{i}}\left( t \right)+c_{\boldsymbol{43}}\cdot\lambda_{{\boldsymbol{G}\boldsymbol{4}}_{i}}\left( t \right)+c_{\boldsymbol{53}}\cdot\lambda_{{\boldsymbol{G}\boldsymbol{5}}_{i}}\left( t \right)+c_{\boldsymbol{63}}\cdot\lambda_{{\boldsymbol{G}\boldsymbol{6}}_{i}}\left( t \right)+c_{\boldsymbol{73}}\cdot\lambda_{{\boldsymbol{G}\boldsymbol{7}}_{i}}\left( t \right)+r_{i}+\pi_{{v1}_{i}}\left( t \right)+\pi_{{v2}_{i}}\left( t \right) \right)\cdot{\boldsymbol{G}\boldsymbol{3}}_{i}\left( t \right)+\omega_{{v1}_{i}}\cdot{\boldsymbol{G}\boldsymbol{3}\boldsymbol{v}\boldsymbol{1}}_{i}\left( t \right)+\omega_{{v2}_{i}}\cdot{\boldsymbol{G}\boldsymbol{3}\boldsymbol{v}\boldsymbol{2}}_{i}\left( t \right)$$

$$\frac{d{\boldsymbol{G}\boldsymbol{3}\boldsymbol{v}\boldsymbol{1}}_{i}(t)}{dt}=\lambda_{{\boldsymbol{G}\boldsymbol{3}}_{i}}\left( t \right)\cdot{\boldsymbol{Sv}\boldsymbol{1}}_{i}\left( t \right)+r_{i}\cdot\left( {\boldsymbol{G}\boldsymbol{1}\boldsymbol{G}\boldsymbol{3}\boldsymbol{v}\boldsymbol{1}}_{i}\left( t \right)+{\boldsymbol{G}\boldsymbol{2}\boldsymbol{G}\boldsymbol{3}\boldsymbol{v}\boldsymbol{1}}_{i}\left( t \right)+{\boldsymbol{G}\boldsymbol{3}\boldsymbol{G}\boldsymbol{4}\boldsymbol{v}\boldsymbol{1}}_{i}\left( t \right)+{\boldsymbol{G}\boldsymbol{3}\boldsymbol{G}\boldsymbol{5}\boldsymbol{v}\boldsymbol{1}}_{i}(t)+{\boldsymbol{G}\boldsymbol{3}\boldsymbol{G}\boldsymbol{6}\boldsymbol{v}\boldsymbol{1}}_{i}(t)+{\boldsymbol{G}\boldsymbol{3}\boldsymbol{G7v}\boldsymbol{1}}_{i}(t) \right)-\left( c_{\boldsymbol{13}}\cdot(1-{v1}_{\boldsymbol{G}\boldsymbol{1}})\cdot\lambda_{{\boldsymbol{G}\boldsymbol{1}}_{i}}\left( t \right)+c_{\boldsymbol{23}}\cdot\lambda_{{\boldsymbol{G}\boldsymbol{2}}_{i}}\left( t \right)+c_{\boldsymbol{43}}\cdot\lambda_{{\boldsymbol{G}\boldsymbol{4}}_{i}}\left( t \right)+c_{\boldsymbol{53}}\cdot\lambda_{{\boldsymbol{G}\boldsymbol{5}}_{i}}\left( t \right)+c_{\boldsymbol{63}}\cdot\lambda_{{\boldsymbol{G}\boldsymbol{6}}_{i}}\left( t \right)+c_{\boldsymbol{73}}\cdot\lambda_{{\boldsymbol{G}\boldsymbol{7}}_{i}}\left( t \right)+r_{i}+\omega_{{v1}_{i}} \right)\cdot{\boldsymbol{G}\boldsymbol{3}\boldsymbol{v}\boldsymbol{1}}_{i}(t)+\pi_{{v1}_{i}}(t)\cdot{\boldsymbol{G}\boldsymbol{3}}_{i}(t)$$

$$\frac{d{\boldsymbol{G}\boldsymbol{3}\boldsymbol{v}\boldsymbol{2}}_{i}(t)}{dt}={(1-{v2}_{\boldsymbol{G}\boldsymbol{3}})\cdot\lambda}_{{\boldsymbol{G}\boldsymbol{3}}_{i}}\left( t \right)\cdot{\boldsymbol{Sv}\boldsymbol{2}}_{i}\left( t \right)+r_{i}\cdot\left( {\boldsymbol{G}\boldsymbol{1}\boldsymbol{G}\boldsymbol{3}\boldsymbol{v2}}_{i}\left( t \right)+{\boldsymbol{G}\boldsymbol{2}\boldsymbol{G}\boldsymbol{3}\boldsymbol{v}\boldsymbol{2}}_{i}\left( t \right)+{\boldsymbol{G}\boldsymbol{3}\boldsymbol{G}\boldsymbol{4}\boldsymbol{v}\boldsymbol{2}}_{i}\left( t \right)+{\boldsymbol{G}\boldsymbol{3}\boldsymbol{G}\boldsymbol{5}\boldsymbol{v}\boldsymbol{2}}_{i}(t)+{\boldsymbol{G}\boldsymbol{3}\boldsymbol{G}\boldsymbol{6}\boldsymbol{v}\boldsymbol{2}}_{i}(t)+{\boldsymbol{G}\boldsymbol{3}\boldsymbol{G}\boldsymbol{7}\boldsymbol{v}\boldsymbol{2}}_{i}(t) \right)-\left( c_{\boldsymbol{13}}\cdot(1-{v2}_{\boldsymbol{G}\boldsymbol{1}})\cdot\lambda_{{\boldsymbol{G}\boldsymbol{1}}_{i}}\left( t \right)+c_{\boldsymbol{23}}\cdot{(1-{v2}_{\boldsymbol{G}\boldsymbol{2}})\cdot\lambda}_{{\boldsymbol{G}\boldsymbol{2}}_{i}}\left( t \right)+c_{\boldsymbol{43}}\cdot\lambda_{{\boldsymbol{G}\boldsymbol{4}}_{i}}\left( t \right)+c_{\boldsymbol{53}}\cdot\lambda_{{\boldsymbol{G}\boldsymbol{5}}_{i}}\left( t \right)+c_{\boldsymbol{63}}\cdot\lambda_{{\boldsymbol{G}\boldsymbol{6}}_{i}}\left( t \right)+c_{\boldsymbol{73}}\cdot\lambda_{{\boldsymbol{G}\boldsymbol{7}}_{i}}\left( t \right)+r_{i}+\omega_{{v2}_{i}} \right)\cdot{\boldsymbol{G}\boldsymbol{3}\boldsymbol{v}\boldsymbol{2}}_{i}(t)+\pi_{{v2}_{i}}(t)\cdot{\boldsymbol{G}\boldsymbol{3}}_{i}(t)$$

$$\frac{d{\boldsymbol{G}\boldsymbol{4}}_{i}(t)}{dt}=\lambda_{{\boldsymbol{G}\boldsymbol{4}}_{i}}\left( t \right)\cdot\boldsymbol{S}_{i}\left( t \right)+r_{i}\cdot\left( {\boldsymbol{G}\boldsymbol{1}\boldsymbol{G}\boldsymbol{4}}_{i}\left( t \right)+{\boldsymbol{G}\boldsymbol{2}\boldsymbol{G}\boldsymbol{4}}_{i}\left( t \right)+{\boldsymbol{G}\boldsymbol{3}\boldsymbol{G}\boldsymbol{4}}_{i}\left( t \right)+{\boldsymbol{G}\boldsymbol{4}\boldsymbol{G}\boldsymbol{5}}_{i}(t)+{\boldsymbol{G}\boldsymbol{4}\boldsymbol{G}\boldsymbol{6}}_{i}(t)+{\boldsymbol{G}\boldsymbol{4}\boldsymbol{G}\boldsymbol{7}}_{i}(t) \right)-\left( c_{\boldsymbol{14}}\cdot\lambda_{{\boldsymbol{G}\boldsymbol{1}}_{i}}\left( t \right)+c_{\boldsymbol{24}}\cdot\lambda_{{\boldsymbol{G}\boldsymbol{2}}_{i}}\left( t \right)+c_{\boldsymbol{34}}\cdot\lambda_{{\boldsymbol{G}\boldsymbol{3}}_{i}}\left( t \right)+c_{\boldsymbol{54}}\cdot\lambda_{{\boldsymbol{G}\boldsymbol{5}}_{i}}\left( t \right)+c_{\boldsymbol{64}}\cdot\lambda_{{\boldsymbol{G}\boldsymbol{6}}_{i}}\left( t \right)+c_{\boldsymbol{74}}\cdot\lambda_{{\boldsymbol{G}\boldsymbol{7}}_{i}}\left( t \right)+r_{i}+\pi_{{v1}_{i}}\left( t \right)+\pi_{{v2}_{i}}\left( t \right) \right)\cdot{\boldsymbol{G}\boldsymbol{4}}_{i}(t)+\omega_{{v1}_{i}}\cdot{\boldsymbol{G}\boldsymbol{4}\boldsymbol{v}\boldsymbol{1}}_{i}\left( t \right)+\omega_{{v2}_{i}}\cdot{\boldsymbol{G}\boldsymbol{4}\boldsymbol{v}\boldsymbol{2}}_{i}\left( t \right)$$

$$\frac{d{\boldsymbol{G}\boldsymbol{4}\boldsymbol{v}\boldsymbol{1}}_{i}(t)}{dt}=\lambda_{{\boldsymbol{G}\boldsymbol{4}}_{i}}\left( t \right)\cdot{\boldsymbol{Sv}\boldsymbol{1}}_{i}\left( t \right)+r_{i}\cdot\left( {\boldsymbol{G}\boldsymbol{1}\boldsymbol{G}\boldsymbol{4}\boldsymbol{v}\boldsymbol{1}}_{i}\left( t \right)+{\boldsymbol{G}\boldsymbol{2}\boldsymbol{G}\boldsymbol{4}\boldsymbol{v}\boldsymbol{1}}_{i}\left( t \right)+{\boldsymbol{G}\boldsymbol{3}\boldsymbol{G}\boldsymbol{4}\boldsymbol{v}\boldsymbol{1}}_{i}\left( t \right)+{\boldsymbol{G}\boldsymbol{4}\boldsymbol{G}\boldsymbol{5}\boldsymbol{v}\boldsymbol{1}}_{i}(t)+{\boldsymbol{G}\boldsymbol{4}\boldsymbol{G}\boldsymbol{6}\boldsymbol{v}\boldsymbol{1}}_{i}(t)+{\boldsymbol{G}\boldsymbol{4}\boldsymbol{G}\boldsymbol{7}\boldsymbol{v}\boldsymbol{1}}_{i}(t) \right)-\left( c_{\boldsymbol{14}}\cdot{(1-{v1}_{\boldsymbol{G}\boldsymbol{1}})\cdot\lambda}_{{\boldsymbol{G}\boldsymbol{1}}_{i}}\left( t \right)+c_{\boldsymbol{24}}\cdot\lambda_{{\boldsymbol{G}\boldsymbol{2}}_{i}}\left( t \right)+c_{\boldsymbol{34}}\cdot\lambda_{{\boldsymbol{G}\boldsymbol{3}}_{i}}\left( t \right)+c_{\boldsymbol{54}}\cdot\lambda_{{\boldsymbol{G}\boldsymbol{5}}_{i}}\left( t \right)+c_{\boldsymbol{64}}\cdot\lambda_{{\boldsymbol{G}\boldsymbol{6}}_{i}}\left( t \right)+c_{\boldsymbol{74}}\cdot\lambda_{{\boldsymbol{G}\boldsymbol{7}}_{i}}\left( t \right)+r_{i}+\omega_{{v1}_{i}} \right)\cdot{\boldsymbol{G}\boldsymbol{4}\boldsymbol{v}\boldsymbol{1}}_{i}(t)+\pi_{{v1}_{i}}(t)\cdot{\boldsymbol{G}\boldsymbol{4}}_{i}(t)$$

$$\frac{d{\boldsymbol{G}\boldsymbol{4}\boldsymbol{v}\boldsymbol{2}}_{i}(t)}{dt}=\lambda_{{\boldsymbol{G}\boldsymbol{4}}_{i}}\left( t \right)\cdot{\boldsymbol{Sv}\boldsymbol{2}}_{i}\left( t \right)+r_{i}\cdot\left( {\boldsymbol{G}\boldsymbol{1}\boldsymbol{G}\boldsymbol{4}\boldsymbol{v}\boldsymbol{2}}_{i}\left( t \right)+{\boldsymbol{G}\boldsymbol{2}\boldsymbol{G}\boldsymbol{4}\boldsymbol{v}\boldsymbol{2}}_{i}\left( t \right)+{\boldsymbol{G}\boldsymbol{3}\boldsymbol{G}\boldsymbol{4}\boldsymbol{v}\boldsymbol{2}}_{i}\left( t \right)+{\boldsymbol{G4G}\boldsymbol{5}\boldsymbol{v}\boldsymbol{2}}_{i}(t)+{\boldsymbol{G}\boldsymbol{4}\boldsymbol{G}\boldsymbol{6}\boldsymbol{v}\boldsymbol{2}}_{i}(t)+{\boldsymbol{G}\boldsymbol{4}\boldsymbol{G}\boldsymbol{7}\boldsymbol{v}\boldsymbol{2}}_{i}(t) \right)-\left( c_{\boldsymbol{14}}\cdot{(1-{v2}_{\boldsymbol{G}\boldsymbol{1}})\cdot\lambda}_{{\boldsymbol{G}\boldsymbol{1}}_{i}}\left( t \right)+c_{\boldsymbol{24}}\cdot{(1-{v2}_{\boldsymbol{G}\boldsymbol{2}})\cdot\lambda}_{{\boldsymbol{G}\boldsymbol{2}}_{i}}\left( t \right)+c_{\boldsymbol{34}}\cdot{(1-{v2}_{\boldsymbol{G}\boldsymbol{3}})\cdot\lambda}_{{\boldsymbol{G}\boldsymbol{3}}_{i}}\left( t \right)+c_{\boldsymbol{54}}\cdot\lambda_{{\boldsymbol{G}\boldsymbol{5}}_{i}}\left( t \right)+c_{\boldsymbol{64}}\cdot\lambda_{{\boldsymbol{G}\boldsymbol{6}}_{i}}\left( t \right)+c_{\boldsymbol{74}}\cdot\lambda_{{\boldsymbol{G}\boldsymbol{7}}_{i}}\left( t \right)+r_{i}+\omega_{{v2}_{i}} \right)\cdot{\boldsymbol{G}\boldsymbol{4}\boldsymbol{v}\boldsymbol{2}}_{i}(t)+\pi_{{v2}_{i}}(t)\cdot{\boldsymbol{G}\boldsymbol{4}}_{i}(t)$$

$$\frac{d\boldsymbol{G5}_{i}(t)}{dt}=\lambda_{{\boldsymbol{G}\boldsymbol{5}}_{i}}\left( t \right)\cdot\boldsymbol{S}_{i}\left( t \right)+r_{i}\cdot\left( {\boldsymbol{G}\boldsymbol{1}\boldsymbol{G}\boldsymbol{5}}_{i}\left( t \right)+{\boldsymbol{G}\boldsymbol{2}\boldsymbol{G}\boldsymbol{5}}_{i}\left( t \right)+{\boldsymbol{G}\boldsymbol{3}\boldsymbol{G}\boldsymbol{5}}_{i}\left( t \right)+{\boldsymbol{G}\boldsymbol{4}\boldsymbol{G}\boldsymbol{5}}_{i}(t)+{\boldsymbol{G}\boldsymbol{5}\boldsymbol{G}\boldsymbol{6}}_{i}(t)+{\boldsymbol{G}\boldsymbol{5}\boldsymbol{G}\boldsymbol{7}}_{i}(t) \right)-\left( c_{\boldsymbol{15}}\cdot\lambda_{{\boldsymbol{G}\boldsymbol{1}}_{i}}\left( t \right)+c_{\boldsymbol{25}}\cdot\lambda_{{\boldsymbol{G}\boldsymbol{2}}_{i}}\left( t \right)+c_{\boldsymbol{35}}\cdot\lambda_{{\boldsymbol{G}\boldsymbol{3}}_{i}}\left( t \right)+c_{\boldsymbol{45}}\cdot\lambda_{{\boldsymbol{G}\boldsymbol{4}}_{i}}\left( t \right)+c_{\boldsymbol{65}}\cdot\lambda_{{\boldsymbol{G}\boldsymbol{6}}_{i}}\left( t \right)+c_{\boldsymbol{75}}\cdot\lambda_{{\boldsymbol{G}\boldsymbol{7}}_{i}}\left( t \right)+r_{i}+\pi_{{v1}_{i}}\left( t \right)+\pi_{{v2}_{i}}\left( t \right) \right)\cdot\boldsymbol{G5}_{i}(t)+\omega_{{v1}_{i}}\cdot{\boldsymbol{G}\boldsymbol{5}\boldsymbol{v}\boldsymbol{1}}_{i}\left( t \right)+\omega_{{v2}_{i}}\cdot{\boldsymbol{G}\boldsymbol{5}\boldsymbol{v}\boldsymbol{2}}_{i}\left( t \right)$$

$$\frac{d{\boldsymbol{G}\boldsymbol{5}\boldsymbol{v}\boldsymbol{1}}_{i}(t)}{dt}=\lambda_{{\boldsymbol{G}\boldsymbol{5}}_{i}}\left( t \right)\cdot{\boldsymbol{Sv}\boldsymbol{1}}_{i}\left( t \right)+r_{i}\cdot\left( {\boldsymbol{G}\boldsymbol{1}\boldsymbol{G}\boldsymbol{5}\boldsymbol{v}\boldsymbol{1}}_{i}\left( t \right)+{\boldsymbol{G}\boldsymbol{2}\boldsymbol{G}\boldsymbol{5}\boldsymbol{v}\boldsymbol{1}}_{i}\left( t \right)+{\boldsymbol{G}\boldsymbol{3}\boldsymbol{G}\boldsymbol{5}\boldsymbol{v}\boldsymbol{1}}_{i}\left( t \right)+{\boldsymbol{G}\boldsymbol{4}\boldsymbol{G}\boldsymbol{5}\boldsymbol{v}\boldsymbol{1}}_{i}(t)+{\boldsymbol{G}\boldsymbol{5}\boldsymbol{G}\boldsymbol{6}\boldsymbol{v}\boldsymbol{1}}_{i}(t)+{\boldsymbol{G}\boldsymbol{5}\boldsymbol{G}\boldsymbol{7}\boldsymbol{v}\boldsymbol{1}}_{i}(t) \right)-\left( c_{\boldsymbol{15}}\cdot{(1-{v1}_{\boldsymbol{G}\boldsymbol{1}})\cdot\lambda}_{{\boldsymbol{G}\boldsymbol{1}}_{i}}\left( t \right)+c_{\boldsymbol{25}}\cdot\lambda_{{\boldsymbol{G}\boldsymbol{2}}_{i}}\left( t \right)+c_{\boldsymbol{35}}\cdot\lambda_{{\boldsymbol{G}\boldsymbol{3}}_{i}}\left( t \right)+c_{\boldsymbol{45}}\cdot\lambda_{{\boldsymbol{G}\boldsymbol{4}}_{i}}\left( t \right)+c_{\boldsymbol{65}}\cdot\lambda_{{\boldsymbol{G}\boldsymbol{6}}_{i}}\left( t \right)+c_{\boldsymbol{75}}\cdot\lambda_{{\boldsymbol{G}\boldsymbol{7}}_{i}}\left( t \right)+r_{i}+\omega_{{v1}_{i}} \right)\cdot{\boldsymbol{G}\boldsymbol{5}\boldsymbol{v}\boldsymbol{1}}_{i}(t)+\pi_{{v1}_{i}}(t)\cdot{\boldsymbol{G}\boldsymbol{5}}_{i}(t)$$

$$\frac{d{\boldsymbol{G}\boldsymbol{5}\boldsymbol{v}\boldsymbol{2}}_{i}(t)}{dt}=\lambda_{{\boldsymbol{G}\boldsymbol{5}}_{i}}\left( t \right)\cdot{\boldsymbol{Sv}\boldsymbol{2}}_{i}\left( t \right)+r_{i}\cdot\left( {\boldsymbol{G}\boldsymbol{1}\boldsymbol{G}\boldsymbol{5}\boldsymbol{v}\boldsymbol{2}}_{i}\left( t \right)+{\boldsymbol{G}\boldsymbol{2}\boldsymbol{G}\boldsymbol{5}\boldsymbol{v}\boldsymbol{2}}_{i}\left( t \right)+{\boldsymbol{G}\boldsymbol{3}\boldsymbol{G}\boldsymbol{5}\boldsymbol{v}\boldsymbol{2}}_{i}\left( t \right)+{\boldsymbol{G}\boldsymbol{4}\boldsymbol{G}\boldsymbol{5}\boldsymbol{v}\boldsymbol{2}}_{i}(t)+{\boldsymbol{G}\boldsymbol{5}\boldsymbol{G}\boldsymbol{6}\boldsymbol{v}\boldsymbol{2}}_{i}(t)+{\boldsymbol{G}\boldsymbol{5}\boldsymbol{G}\boldsymbol{7}\boldsymbol{v}\boldsymbol{2}}_{i}(t) \right)-\left( c_{\boldsymbol{15}}\cdot{(1-{v2}_{\boldsymbol{G}\boldsymbol{1}})\cdot\lambda}_{{\boldsymbol{G}\boldsymbol{1}}_{i}}\left( t \right)+c_{\boldsymbol{25}}\cdot{(1-{v2}_{\boldsymbol{G}\boldsymbol{2}})\cdot\lambda}_{{\boldsymbol{G}\boldsymbol{2}}_{i}}\left( t \right)+c_{\boldsymbol{35}}\cdot{(1-{v2}_{\boldsymbol{G}\boldsymbol{3}})\cdot\lambda}_{{\boldsymbol{G}\boldsymbol{3}}_{i}}\left( t \right)+c_{\boldsymbol{45}}\cdot\lambda_{{\boldsymbol{G}\boldsymbol{4}}_{i}}\left( t \right)+c_{\boldsymbol{65}}\cdot\lambda_{{\boldsymbol{G}\boldsymbol{6}}_{i}}\left( t \right)+c_{\boldsymbol{75}}\cdot\lambda_{{\boldsymbol{G}\boldsymbol{7}}_{i}}\left( t \right)+r_{i}+\omega_{{v2}_{i}} \right)\cdot{\boldsymbol{G}\boldsymbol{5}\boldsymbol{v}\boldsymbol{2}}_{i}(t)+\pi_{{v2}_{i}}(t)\cdot{\boldsymbol{G}\boldsymbol{5}}_{i}(t)$$

$$\frac{d{\boldsymbol{G}\boldsymbol{6}}_{i}(t)}{dt}=\lambda_{{\boldsymbol{G}\boldsymbol{6}}_{i}}\left( t \right)\cdot\boldsymbol{S}_{i}\left( t \right)+r_{i}\cdot\left( {\boldsymbol{G}\boldsymbol{1}\boldsymbol{G}\boldsymbol{6}}_{i}\left( t \right)+{\boldsymbol{G}\boldsymbol{2}\boldsymbol{G}\boldsymbol{6}}_{i}\left( t \right)+{\boldsymbol{G}\boldsymbol{3}\boldsymbol{G}\boldsymbol{6}}_{i}\left( t \right)+{\boldsymbol{G}\boldsymbol{4}\boldsymbol{G}\boldsymbol{6}}_{i}(t)+{\boldsymbol{G}\boldsymbol{5}\boldsymbol{G}\boldsymbol{6}}_{i}(t)+{\boldsymbol{G}\boldsymbol{6}\boldsymbol{G}\boldsymbol{7}}_{i}(t) \right)-\left( c_{\boldsymbol{16}}\cdot\lambda_{{\boldsymbol{G}\boldsymbol{1}}_{i}}\left( t \right)+c_{\boldsymbol{26}}\cdot\lambda_{{\boldsymbol{G}\boldsymbol{2}}_{i}}\left( t \right)+c_{\boldsymbol{36}}\cdot\lambda_{{\boldsymbol{G}\boldsymbol{3}}_{i}}\left( t \right)+c_{\boldsymbol{46}}\cdot\lambda_{{\boldsymbol{G}\boldsymbol{4}}_{i}}\left( t \right)+c_{\boldsymbol{56}}\cdot\lambda_{{\boldsymbol{G}\boldsymbol{5}}_{i}}\left( t \right)+c_{\boldsymbol{76}}\cdot\lambda_{{\boldsymbol{G}\boldsymbol{7}}_{i}}\left( t \right)+r_{i}+\pi_{{v1}_{i}}\left( t \right)+\pi_{{v2}_{i}}\left( t \right) \right)\cdot{\boldsymbol{G}\boldsymbol{6}}_{i}(t)+\omega_{{v1}_{i}}\cdot{\boldsymbol{G}\boldsymbol{6}\boldsymbol{v}\boldsymbol{1}}_{i}\left( t \right)+\omega_{{v2}_{i}}\cdot{\boldsymbol{G}\boldsymbol{6}\boldsymbol{v}\boldsymbol{2}}_{i}\left( t \right)$$

$$\frac{d{\boldsymbol{G}\boldsymbol{6}\boldsymbol{v}\boldsymbol{1}}_{i}(t)}{dt}=\lambda_{{\boldsymbol{G}\boldsymbol{6}}_{i}}\left( t \right)\cdot{\boldsymbol{Sv}\boldsymbol{1}}_{i}\left( t \right)+r_{i}\cdot\left( {\boldsymbol{G}\boldsymbol{1}\boldsymbol{G}\boldsymbol{6}\boldsymbol{v}\boldsymbol{1}}_{i}\left( t \right)+{\boldsymbol{G}\boldsymbol{2}\boldsymbol{G}\boldsymbol{6}\boldsymbol{v}\boldsymbol{1}}_{i}\left( t \right)+{\boldsymbol{G}\boldsymbol{3}\boldsymbol{G}\boldsymbol{6}\boldsymbol{v}\boldsymbol{1}}_{i}\left( t \right)+{\boldsymbol{G}\boldsymbol{4}\boldsymbol{G}\boldsymbol{6}\boldsymbol{v}\boldsymbol{1}}_{i}(t)+{\boldsymbol{G}\boldsymbol{5}\boldsymbol{G}\boldsymbol{6}\boldsymbol{v}\boldsymbol{1}}_{i}(t)+{\boldsymbol{G}\boldsymbol{6}\boldsymbol{G}\boldsymbol{7}\boldsymbol{v}\boldsymbol{1}}_{i}(t) \right)-\left( c_{\boldsymbol{16}}\cdot{(1-{v1}_{\boldsymbol{G}\boldsymbol{1}})\cdot\lambda}_{{\boldsymbol{G}\boldsymbol{1}}_{i}}\left( t \right)+c_{\boldsymbol{26}}\cdot\lambda_{{\boldsymbol{G}\boldsymbol{2}}_{i}}\left( t \right)+c_{\boldsymbol{36}}\cdot\lambda_{{\boldsymbol{G}\boldsymbol{3}}_{i}}\left( t \right)+c_{\boldsymbol{46}}\cdot\lambda_{{\boldsymbol{G}\boldsymbol{4}}_{i}}\left( t \right)+c_{\boldsymbol{56}}\cdot\lambda_{{\boldsymbol{G}\boldsymbol{5}}_{i}}\left( t \right)+c_{\boldsymbol{76}}\cdot\lambda_{{\boldsymbol{G}\boldsymbol{7}}_{i}}\left( t \right)+r_{i}+\omega_{{v1}_{i}} \right)\cdot{\boldsymbol{G}\boldsymbol{6}\boldsymbol{v}\boldsymbol{1}}_{i}(t)+\pi_{{v1}_{i}}(t)\cdot{\boldsymbol{G}\boldsymbol{6}}_{i}(t)$$

$$\frac{d{\boldsymbol{G}\boldsymbol{6}\boldsymbol{v}\boldsymbol{2}}_{i}(t)}{dt}=\lambda_{{\boldsymbol{G}\boldsymbol{6}}_{i}}\left( t \right)\cdot{\boldsymbol{Sv}\boldsymbol{2}}_{i}\left( t \right)+r_{i}\cdot\left( {\boldsymbol{G}\boldsymbol{1}\boldsymbol{G}\boldsymbol{6}\boldsymbol{v}\boldsymbol{2}}_{i}\left( t \right)+{\boldsymbol{G}\boldsymbol{2}\boldsymbol{G}\boldsymbol{6}\boldsymbol{v}\boldsymbol{2}}_{i}\left( t \right)+{\boldsymbol{G}\boldsymbol{3}\boldsymbol{G}\boldsymbol{6}\boldsymbol{v}\boldsymbol{2}}_{i}\left( t \right)+{\boldsymbol{G}\boldsymbol{4}\boldsymbol{G}\boldsymbol{6}\boldsymbol{v}\boldsymbol{2}}_{i}(t)+{\boldsymbol{G}\boldsymbol{5}\boldsymbol{G}\boldsymbol{6}\boldsymbol{v}\boldsymbol{2}}_{i}(t)+{\boldsymbol{G}\boldsymbol{6}\boldsymbol{G}\boldsymbol{7}\boldsymbol{v}\boldsymbol{2}}_{i}(t) \right)-\left( c_{\boldsymbol{16}}\cdot{(1-{v2}_{\boldsymbol{G}\boldsymbol{1}})\cdot\lambda}_{{\boldsymbol{G}\boldsymbol{1}}_{i}}\left( t \right)+c_{\boldsymbol{26}}\cdot{(1-{v2}_{\boldsymbol{G}\boldsymbol{2}})\cdot\lambda}_{{\boldsymbol{G}\boldsymbol{2}}_{i}}\left( t \right)+c_{\boldsymbol{36}}\cdot{(1-{v2}_{\boldsymbol{G}\boldsymbol{3}})\cdot\lambda}_{{\boldsymbol{G}\boldsymbol{3}}_{i}}\left( t \right)+c_{\boldsymbol{46}}\cdot\lambda_{{\boldsymbol{G}\boldsymbol{4}}_{i}}\left( t \right)+c_{\boldsymbol{56}}\cdot\lambda_{{\boldsymbol{G}\boldsymbol{5}}_{i}}\left( t \right)+c_{\boldsymbol{76}}\cdot\lambda_{{\boldsymbol{G}\boldsymbol{7}}_{i}}\left( t \right)+r_{i}+\omega_{{v2}_{i}} \right)\cdot{\boldsymbol{G}\boldsymbol{6}\boldsymbol{v}\boldsymbol{2}}_{i}(t)+\pi_{{v2}_{i}}(t)\cdot{\boldsymbol{G}\boldsymbol{6}}_{i}(t)$$

$$\frac{d{\boldsymbol{G}\boldsymbol{7}}_{i}(t)}{dt}=\lambda_{{\boldsymbol{G}\boldsymbol{7}}_{i}}\left( t \right)\cdot\boldsymbol{S}_{i}\left( t \right)+r_{i}\cdot\left( {\boldsymbol{G}\boldsymbol{1}\boldsymbol{G}\boldsymbol{7}}_{i}\left( t \right)+{\boldsymbol{G}\boldsymbol{2}\boldsymbol{G}\boldsymbol{7}}_{i}\left( t \right)+{\boldsymbol{G}\boldsymbol{3}\boldsymbol{G}\boldsymbol{7}}_{i}\left( t \right)+{\boldsymbol{G}\boldsymbol{4}\boldsymbol{G}\boldsymbol{7}}_{i}(t)+{\boldsymbol{G}\boldsymbol{5}\boldsymbol{G}\boldsymbol{7}}_{i}(t)+{\boldsymbol{G}\boldsymbol{6}\boldsymbol{G}\boldsymbol{7}}_{i}(t) \right)-\left( c_{\boldsymbol{17}}\cdot\lambda_{{\boldsymbol{G}\boldsymbol{1}}_{i}}\left( t \right)+c_{\boldsymbol{27}}\cdot\lambda_{{\boldsymbol{G}\boldsymbol{2}}_{i}}\left( t \right)+c_{\boldsymbol{37}}\cdot\lambda_{{\boldsymbol{G}\boldsymbol{3}}_{i}}\left( t \right)+c_{\boldsymbol{47}}\cdot\lambda_{{\boldsymbol{G}\boldsymbol{4}}_{i}}\left( t \right)+c_{\boldsymbol{57}}\cdot\lambda_{{\boldsymbol{G}\boldsymbol{5}}_{i}}\left( t \right)+c_{\boldsymbol{67}}\cdot\lambda_{{\boldsymbol{G}\boldsymbol{6}}_{i}}\left( t \right)+r_{i}+\pi_{{v1}_{i}}\left( t \right)+\pi_{{v2}_{i}}\left( t \right) \right)\cdot{\boldsymbol{G}\boldsymbol{7}}_{i}(t)+\omega_{{v1}_{i}}\cdot{\boldsymbol{G}\boldsymbol{7}\boldsymbol{v}\boldsymbol{1}}_{i}\left( t \right)+\omega_{{v2}_{i}}\cdot{\boldsymbol{G}\boldsymbol{7}\boldsymbol{v}\boldsymbol{2}}_{i}\left( t \right)$$

$$\frac{d{\boldsymbol{G}\boldsymbol{7}\boldsymbol{v}\boldsymbol{1}}_{i}(t)}{dt}=\lambda_{{\boldsymbol{G}\boldsymbol{7}}_{i}}\left( t \right)\cdot{\boldsymbol{Sv}\boldsymbol{1}}_{i}\left( t \right)+r_{i}\cdot\left( {\boldsymbol{G}\boldsymbol{1}\boldsymbol{G}\boldsymbol{7}\boldsymbol{v}\boldsymbol{1}}_{i}\left( t \right)+{\boldsymbol{G}\boldsymbol{2}\boldsymbol{G}\boldsymbol{7}\boldsymbol{v}\boldsymbol{1}}_{i}\left( t \right)+{\boldsymbol{G}\boldsymbol{3}\boldsymbol{G}\boldsymbol{7}\boldsymbol{v}\boldsymbol{1}}_{i}\left( t \right)+{\boldsymbol{G}\boldsymbol{4}\boldsymbol{G}\boldsymbol{7}\boldsymbol{v}\boldsymbol{1}}_{i}(t)+{\boldsymbol{G}\boldsymbol{5}\boldsymbol{G}\boldsymbol{7}\boldsymbol{v}\boldsymbol{1}}_{i}(t)+{\boldsymbol{G}\boldsymbol{6}\boldsymbol{G}\boldsymbol{7}\boldsymbol{v}\boldsymbol{1}}_{i}(t) \right)-\left( c_{\boldsymbol{17}}\cdot{(1-{v1}_{\boldsymbol{G}\boldsymbol{1}})\cdot\lambda}_{{\boldsymbol{G}\boldsymbol{1}}_{i}}\left( t \right)+c_{\boldsymbol{27}}\cdot\lambda_{{\boldsymbol{G}\boldsymbol{2}}_{i}}\left( t \right)+c_{\boldsymbol{37}}\cdot\lambda_{{\boldsymbol{G}\boldsymbol{3}}_{i}}\left( t \right)+c_{\boldsymbol{47}}\cdot\lambda_{{\boldsymbol{G}\boldsymbol{4}}_{i}}\left( t \right)+c_{\boldsymbol{57}}\cdot\lambda_{{\boldsymbol{G}\boldsymbol{5}}_{i}}\left( t \right)+c_{\boldsymbol{67}}\cdot\lambda_{{\boldsymbol{G}\boldsymbol{6}}_{i}}\left( t \right)+r_{i}+\omega_{{v1}_{i}} \right)\cdot{\boldsymbol{G}\boldsymbol{7}\boldsymbol{v}\boldsymbol{1}}_{i}(t)+\pi_{{v1}_{i}}(t)\cdot{\boldsymbol{G}\boldsymbol{7}}_{i}(t)$$

$$\frac{d{\boldsymbol{G}\boldsymbol{7}\boldsymbol{v}\boldsymbol{2}}_{i}(t)}{dt}=\lambda_{{\boldsymbol{G}\boldsymbol{7}}_{i}}\left( t \right)\cdot{\boldsymbol{Sv}\boldsymbol{2}}_{i}\left( t \right)+r_{i}\cdot\left( {\boldsymbol{G}\boldsymbol{1}\boldsymbol{G}\boldsymbol{7}\boldsymbol{v}\boldsymbol{2}}_{i}\left( t \right)+{\boldsymbol{G}\boldsymbol{2}\boldsymbol{G}\boldsymbol{7}\boldsymbol{v}\boldsymbol{2}}_{i}\left( t \right)+{\boldsymbol{G}\boldsymbol{3}\boldsymbol{G}\boldsymbol{7}\boldsymbol{v}\boldsymbol{2}}_{i}\left( t \right)+{\boldsymbol{G}\boldsymbol{4}\boldsymbol{G}\boldsymbol{7}\boldsymbol{v}\boldsymbol{2}}_{i}(t)+{\boldsymbol{G}\boldsymbol{5}\boldsymbol{G}\boldsymbol{7}\boldsymbol{v}\boldsymbol{2}}_{i}(t)+{\boldsymbol{G}\boldsymbol{6}\boldsymbol{G}\boldsymbol{7}\boldsymbol{v}\boldsymbol{2}}_{i}(t) \right)-\left( c_{\boldsymbol{17}}\cdot{(1-{v2}_{\boldsymbol{G}\boldsymbol{1}})\cdot\lambda}_{{\boldsymbol{G}\boldsymbol{1}}_{i}}\left( t \right)+c_{\boldsymbol{27}}\cdot{(1-{v2}_{\boldsymbol{G}\boldsymbol{2}})\cdot\lambda}_{{\boldsymbol{G}\boldsymbol{2}}_{i}}\left( t \right)+c_{\boldsymbol{37}}\cdot{(1-{v2}_{\boldsymbol{G}\boldsymbol{3}})\cdot\lambda}_{{\boldsymbol{G}\boldsymbol{3}}_{i}}\left( t \right)+c_{\boldsymbol{47}}\cdot\lambda_{{\boldsymbol{G}\boldsymbol{4}}_{i}}\left( t \right)+c_{\boldsymbol{57}}\cdot\lambda_{{\boldsymbol{G}\boldsymbol{5}}_{i}}\left( t \right)+c_{\boldsymbol{67}}\cdot\lambda_{{\boldsymbol{G}\boldsymbol{6}}_{i}}\left( t \right)+r_{i}+\omega_{{v2}_{i}} \right)\cdot{\boldsymbol{G}\boldsymbol{7}\boldsymbol{v}\boldsymbol{2}}_{i}(t)+\pi_{{v2}_{i}}(t)\cdot{\boldsymbol{G}\boldsymbol{7}}_{i}(t)$$

$$\frac{d{\boldsymbol{G}\boldsymbol{1}\boldsymbol{G}\boldsymbol{2}}_{i}\left( t \right)}{dt}={c_{\boldsymbol{21}}\cdot\lambda_{{\boldsymbol{G}\boldsymbol{2}}_{i}}\left( t \right)\cdot{\boldsymbol{G}\boldsymbol{1}}_{i}\left( t \right)+c}_{\boldsymbol{12}}\cdot\lambda_{{\boldsymbol{G}\boldsymbol{1}}_{i}}\left( t \right)\cdot{\boldsymbol{G}\boldsymbol{2}}_{i}\left( t \right)-\left( 2\cdot r_{i}+\pi_{{v1}_{i}}\left( t \right)+\pi_{{v2}_{i}}\left( t \right) \right)\cdot{\boldsymbol{G}\boldsymbol{1}\boldsymbol{G}\boldsymbol{2}}_{i}\left( t \right)+\omega_{{v1}_{i}}\cdot{\boldsymbol{G}\boldsymbol{1}\boldsymbol{G}\boldsymbol{2}\boldsymbol{v}\boldsymbol{1}}_{i}\left( t \right)+\omega_{{v2}_{i}}\cdot{\boldsymbol{G}\boldsymbol{1}\boldsymbol{G}\boldsymbol{2}\boldsymbol{v}\boldsymbol{2}}_{i}\left( t \right)$$

$$\frac{d{\boldsymbol{G}\boldsymbol{1}\boldsymbol{G}\boldsymbol{2}\boldsymbol{v}\boldsymbol{1}}_{i}\left( t \right)}{dt}={c_{\boldsymbol{21}}\cdot\lambda_{{\boldsymbol{G}\boldsymbol{2}}_{i}}\left( t \right)\cdot{\boldsymbol{G}\boldsymbol{1}\boldsymbol{v}\boldsymbol{1}}_{i}\left( t \right)+c}_{\boldsymbol{12}}\cdot\left( 1-{v1}_{\boldsymbol{G}\boldsymbol{1}} \right)\cdot\lambda_{{\boldsymbol{G}\boldsymbol{1}}_{i}}\left( t \right)\cdot{\boldsymbol{G}\boldsymbol{2}\boldsymbol{v}\boldsymbol{1}}_{i}\left( t \right)-\left( 2\cdot r_{i}+\omega_{{v1}_{i}} \right)\cdot{\boldsymbol{G}\boldsymbol{1}\boldsymbol{G}\boldsymbol{2}\boldsymbol{v}\boldsymbol{1}}_{i}\left( t \right)+\pi_{{v1}_{i}}(t)\cdot{\boldsymbol{G}\boldsymbol{1}\boldsymbol{G}\boldsymbol{2}}_{i}(t)$$

$$\frac{d{\boldsymbol{G}\boldsymbol{1}\boldsymbol{G}\boldsymbol{2}\boldsymbol{v}\boldsymbol{2}}_{i}\left( t \right)}{dt}={c_{\boldsymbol{21}}\cdot{(1-{v2}_{\boldsymbol{G}\boldsymbol{2}})\cdot\lambda}_{{\boldsymbol{G}\boldsymbol{2}}_{i}}\left( t \right)\cdot{\boldsymbol{G}\boldsymbol{1}\boldsymbol{v}\boldsymbol{2}}_{i}\left( t \right)+c}_{\boldsymbol{12}}\cdot\left( 1-{v2}_{\boldsymbol{G}\boldsymbol{1}} \right)\cdot\lambda_{{\boldsymbol{G}\boldsymbol{1}}_{i}}\left( t \right)\cdot{\boldsymbol{G}\boldsymbol{2}\boldsymbol{v}\boldsymbol{2}}_{i}\left( t \right)-\left( 2\cdot r_{i}+\omega_{{v2}_{i}} \right)\cdot{\boldsymbol{G}\boldsymbol{1}\boldsymbol{G}\boldsymbol{2}\boldsymbol{v}\boldsymbol{2}}_{i}\left( t \right)+\pi_{{v2}_{i}}(t)\cdot{\boldsymbol{G}\boldsymbol{1}\boldsymbol{G}\boldsymbol{2}}_{i}(t)$$

$$\frac{d{\boldsymbol{G}\boldsymbol{1}\boldsymbol{G}\boldsymbol{3}}_{i}\left( t \right)}{dt}={c_{\boldsymbol{31}}\cdot\lambda_{{\boldsymbol{G}\boldsymbol{3}}_{i}}\left( t \right)\cdot{\boldsymbol{G}\boldsymbol{1}}_{i}\left( t \right)+c}_{\boldsymbol{13}}\cdot\lambda_{{\boldsymbol{G}\boldsymbol{1}}_{i}}\left( t \right)\cdot{\boldsymbol{G}\boldsymbol{3}}_{i}\left( t \right)-\left( 2\cdot r_{i}+\pi_{{v1}_{i}}\left( t \right)+\pi_{{v2}_{i}}\left( t \right) \right)\cdot{\boldsymbol{G}\boldsymbol{1}\boldsymbol{G}\boldsymbol{3}}_{i}\left( t \right)+\omega_{{v1}_{i}}\cdot{\boldsymbol{G}\boldsymbol{1}\boldsymbol{G}\boldsymbol{3}\boldsymbol{v}\boldsymbol{1}}_{i}\left( t \right)+\omega_{{v2}_{i}}\cdot{\boldsymbol{G}\boldsymbol{1}\boldsymbol{G}\boldsymbol{3}\boldsymbol{v}\boldsymbol{2}}_{i}\left( t \right)$$

$$\frac{d{\boldsymbol{G}\boldsymbol{1}\boldsymbol{G}\boldsymbol{3}\boldsymbol{v}\boldsymbol{1}}_{i}\left( t \right)}{dt}={c_{\boldsymbol{31}}\cdot\lambda_{{\boldsymbol{G}\boldsymbol{3}}_{i}}\left( t \right)\cdot{\boldsymbol{G}\boldsymbol{1}\boldsymbol{v}\boldsymbol{1}}_{i}\left( t \right)+c}_{\boldsymbol{13}}\cdot{(1-{v1}_{\boldsymbol{G}\boldsymbol{1}})\cdot\lambda}_{{\boldsymbol{G}\boldsymbol{1}}_{i}}\left( t \right)\cdot{\boldsymbol{G}\boldsymbol{3}\boldsymbol{v}\boldsymbol{1}}_{i}\left( t \right)-\left( 2\cdot r_{i}+\omega_{{v1}_{i}} \right)\cdot{\boldsymbol{G}\boldsymbol{1}\boldsymbol{G}\boldsymbol{3}\boldsymbol{v}\boldsymbol{1}}_{i}\left( t \right)+\pi_{{v1}_{i}}(t)\cdot{\boldsymbol{G}\boldsymbol{1}\boldsymbol{G}\boldsymbol{3}}_{i}(t)$$

$$\frac{d{\boldsymbol{G}\boldsymbol{1}\boldsymbol{G}\boldsymbol{3}\boldsymbol{v}\boldsymbol{2}}_{i}\left( t \right)}{dt}={c_{\boldsymbol{31}}\cdot{(1-{v2}_{\boldsymbol{G}\boldsymbol{3}})\cdot\lambda}_{{\boldsymbol{G}\boldsymbol{3}}_{i}}\left( t \right)\cdot{\boldsymbol{G}\boldsymbol{1}\boldsymbol{v}\boldsymbol{2}}_{i}\left( t \right)+c}_{\boldsymbol{13}}\cdot{(1-{v2}_{\boldsymbol{G}\boldsymbol{1}})\cdot\lambda}_{{\boldsymbol{G}\boldsymbol{1}}_{i}}\left( t \right)\cdot{\boldsymbol{G}\boldsymbol{3}\boldsymbol{v}\boldsymbol{2}}_{i}\left( t \right)-\left( 2\cdot r_{i}+\omega_{{v2}_{i}} \right)\cdot{\boldsymbol{G}\boldsymbol{1}\boldsymbol{G}\boldsymbol{3}\boldsymbol{v}\boldsymbol{2}}_{i}\left( t \right)+\pi_{{v2}_{i}}(t)\cdot{\boldsymbol{G}\boldsymbol{1}\boldsymbol{G}\boldsymbol{3}}_{i}(t)$$

$$\frac{d{\boldsymbol{G}\boldsymbol{1}\boldsymbol{G}\boldsymbol{4}}_{i}\left( t \right)}{dt}={c_{\boldsymbol{41}}\cdot\lambda_{{\boldsymbol{G}\boldsymbol{4}}_{i}}\left( t \right)\cdot{\boldsymbol{G}\boldsymbol{1}}_{i}\left( t \right)+c}_{\boldsymbol{14}}\cdot\lambda_{{\boldsymbol{G}\boldsymbol{1}}_{i}}\left( t \right)\cdot{\boldsymbol{G}\boldsymbol{4}}_{i}\left( t \right)-\left( 2\cdot r_{i}+\pi_{{v1}_{i}}\left( t \right)+\pi_{{v2}_{i}}\left( t \right) \right)\cdot{\boldsymbol{G}\boldsymbol{1}\boldsymbol{G}\boldsymbol{4}}_{i}\left( t \right)+\omega_{{v1}_{i}}\cdot{\boldsymbol{G}\boldsymbol{1}\boldsymbol{G}\boldsymbol{4}\boldsymbol{v}\boldsymbol{1}}_{i}\left( t \right)+\omega_{{v2}_{i}}\cdot{\boldsymbol{G}\boldsymbol{1}\boldsymbol{G}\boldsymbol{4}\boldsymbol{v}\boldsymbol{2}}_{i}\left( t \right)$$

$$\frac{d{\boldsymbol{G}\boldsymbol{1}\boldsymbol{G}\boldsymbol{4}\boldsymbol{v}\boldsymbol{1}}_{i}\left( t \right)}{dt}={c_{\boldsymbol{41}}\cdot\lambda_{{\boldsymbol{G}\boldsymbol{4}}_{i}}\left( t \right)\cdot{\boldsymbol{G}\boldsymbol{1}\boldsymbol{v}\boldsymbol{1}}_{i}\left( t \right)+c}_{\boldsymbol{14}}\cdot{(1-{v1}_{\boldsymbol{G}\boldsymbol{1}})\cdot\lambda}_{{\boldsymbol{G}\boldsymbol{1}}_{i}}\left( t \right)\cdot{\boldsymbol{G}\boldsymbol{4}\boldsymbol{v}\boldsymbol{1}}_{i}\left( t \right)-\left( 2\cdot r_{i}+\omega_{{v1}_{i}} \right)\cdot{\boldsymbol{G}\boldsymbol{1}\boldsymbol{G}\boldsymbol{4}\boldsymbol{v}\boldsymbol{1}}_{i}\left( t \right)+\pi_{{v1}_{i}}(t)\cdot{\boldsymbol{G}\boldsymbol{1}\boldsymbol{G}\boldsymbol{4}}_{i}(t)$$

$$\frac{d{\boldsymbol{G}\boldsymbol{1}\boldsymbol{G}\boldsymbol{4}\boldsymbol{v}\boldsymbol{2}}_{i}\left( t \right)}{dt}={c_{\boldsymbol{41}}\cdot\lambda_{{\boldsymbol{G}\boldsymbol{4}}_{i}}\left( t \right)\cdot{\boldsymbol{G}\boldsymbol{1}\boldsymbol{v}\boldsymbol{2}}_{i}\left( t \right)+c}_{\boldsymbol{14}}\cdot{(1-{v2}_{\boldsymbol{G}\boldsymbol{1}})\cdot\lambda}_{{\boldsymbol{G}\boldsymbol{1}}_{i}}\left( t \right)\cdot{\boldsymbol{G}\boldsymbol{4}\boldsymbol{v}\boldsymbol{2}}_{i}\left( t \right)-\left( 2\cdot r_{i}+\omega_{{v2}_{i}} \right)\cdot{\boldsymbol{G}\boldsymbol{1}\boldsymbol{G}\boldsymbol{4}\boldsymbol{v}\boldsymbol{2}}_{i}\left( t \right)+\pi_{{v2}_{i}}(t)\cdot{\boldsymbol{G}\boldsymbol{1}\boldsymbol{G}\boldsymbol{4}}_{i}(t)$$

$$\frac{d{\boldsymbol{G}\boldsymbol{1}\boldsymbol{G}\boldsymbol{5}}_{i}\left( t \right)}{dt}={c_{\boldsymbol{51}}\cdot\lambda_{{\boldsymbol{G}\boldsymbol{5}}_{i}}\left( t \right)\cdot{\boldsymbol{G}\boldsymbol{1}}_{i}\left( t \right)+c}_{\boldsymbol{15}}\cdot\lambda_{{\boldsymbol{G}\boldsymbol{1}}_{i}}\left( t \right)\cdot{\boldsymbol{G}\boldsymbol{5}}_{i}\left( t \right)-\left( 2\cdot r_{i}+\pi_{{v1}_{i}}\left( t \right)+\pi_{{v2}_{i}}\left( t \right) \right)\cdot{\boldsymbol{G}\boldsymbol{1}\boldsymbol{G}\boldsymbol{5}}_{i}\left( t \right)+\omega_{{v1}_{i}}\cdot{\boldsymbol{G}\boldsymbol{1}\boldsymbol{G}\boldsymbol{5}\boldsymbol{v}\boldsymbol{1}}_{i}\left( t \right)+\omega_{{v2}_{i}}\cdot{\boldsymbol{G}\boldsymbol{1}\boldsymbol{G}\boldsymbol{5}\boldsymbol{v}\boldsymbol{2}}_{i}\left( t \right)$$

$$\frac{d{\boldsymbol{G}\boldsymbol{1}\boldsymbol{G5}\boldsymbol{v}\boldsymbol{1}}_{i}\left( t \right)}{dt}={c_{\boldsymbol{51}}\cdot\lambda_{{\boldsymbol{G}\boldsymbol{5}}_{i}}\left( t \right)\cdot{\boldsymbol{G}\boldsymbol{1}\boldsymbol{v}\boldsymbol{1}}_{i}\left( t \right)+c}_{\boldsymbol{15}}\cdot(1-{v1}_{\boldsymbol{G}\boldsymbol{1}})\cdot\lambda_{{\boldsymbol{G}\boldsymbol{1}}_{i}}\left( t \right)\cdot{\boldsymbol{G}\boldsymbol{5}\boldsymbol{v}\boldsymbol{1}}_{i}\left( t \right)-\left( 2\cdot r_{i}+\omega_{{v1}_{i}} \right)\cdot{\boldsymbol{G}\boldsymbol{1}\boldsymbol{G}\boldsymbol{5}\boldsymbol{v}\boldsymbol{1}}_{i}\left( t \right)+\pi_{{v1}_{i}}(t)\cdot{\boldsymbol{G}\boldsymbol{1}\boldsymbol{G}\boldsymbol{5}}_{i}(t)$$

$$\frac{d{\boldsymbol{G}\boldsymbol{1}\boldsymbol{G}\boldsymbol{5}\boldsymbol{v}\boldsymbol{2}}_{i}\left( t \right)}{dt}={c_{\boldsymbol{51}}\cdot\lambda_{{\boldsymbol{G}\boldsymbol{5}}_{i}}\left( t \right)\cdot{\boldsymbol{G}\boldsymbol{1}\boldsymbol{v}\boldsymbol{2}}_{i}\left( t \right)+c}_{\boldsymbol{15}}\cdot(1-{v2}_{\boldsymbol{G}\boldsymbol{1}})\cdot\lambda_{{\boldsymbol{G}\boldsymbol{1}}_{i}}\left( t \right)\cdot{\boldsymbol{G}\boldsymbol{5}\boldsymbol{v}\boldsymbol{2}}_{i}\left( t \right)-\left( 2\cdot r_{i}+\omega_{{v2}_{i}} \right)\cdot{\boldsymbol{G}\boldsymbol{1}\boldsymbol{G}\boldsymbol{5}\boldsymbol{v}\boldsymbol{2}}_{i}\left( t \right)+\pi_{{v2}_{i}}(t)\cdot{\boldsymbol{G}\boldsymbol{1}\boldsymbol{G}\boldsymbol{5}}_{i}(t)$$

$$\frac{d{\boldsymbol{G}\boldsymbol{1}\boldsymbol{G}\boldsymbol{6}}_{i}\left( t \right)}{dt}={c_{\boldsymbol{61}}\cdot\lambda_{{\boldsymbol{G}\boldsymbol{6}}_{i}}\left( t \right)\cdot{\boldsymbol{G}\boldsymbol{1}}_{i}\left( t \right)+c}_{\boldsymbol{16}}\cdot\lambda_{{\boldsymbol{G}\boldsymbol{1}}_{i}}\left( t \right)\cdot{\boldsymbol{G}\boldsymbol{6}}_{i}\left( t \right)-\left( 2\cdot r_{i}+\pi_{{v1}_{i}}\left( t \right)+\pi_{{v2}_{i}}\left( t \right) \right)\cdot{\boldsymbol{G}\boldsymbol{1}\boldsymbol{G}\boldsymbol{6}}_{i}\left( t \right)+\omega_{{v1}_{i}}\cdot{\boldsymbol{G}\boldsymbol{1}\boldsymbol{G}\boldsymbol{6}\boldsymbol{v}\boldsymbol{1}}_{i}\left( t \right)+\omega_{{v2}_{i}}\cdot{\boldsymbol{G}\boldsymbol{1}\boldsymbol{G}\boldsymbol{6}\boldsymbol{v}\boldsymbol{2}}_{i}\left( t \right)$$

$$\frac{d{\boldsymbol{G}\boldsymbol{1}\boldsymbol{G}\boldsymbol{6}\boldsymbol{v}\boldsymbol{1}}_{i}\left( t \right)}{dt}={c_{\boldsymbol{61}}\cdot\lambda_{{\boldsymbol{G}\boldsymbol{6}}_{i}}\left( t \right)\cdot{\boldsymbol{G1}\boldsymbol{v}\boldsymbol{1}}_{i}\left( t \right)+c}_{\boldsymbol{16}}\cdot(1-{v1}_{\boldsymbol{G}\boldsymbol{1}})\cdot\lambda_{{\boldsymbol{G}\boldsymbol{1}}_{i}}\left( t \right)\cdot{\boldsymbol{G}\boldsymbol{6}\boldsymbol{v}\boldsymbol{1}}_{i}\left( t \right)-\left( 2\cdot r_{i}+\omega_{{v1}_{i}} \right)\cdot{\boldsymbol{G}\boldsymbol{1}\boldsymbol{G}\boldsymbol{6}\boldsymbol{v}\boldsymbol{1}}_{i}\left( t \right)+\pi_{{v1}_{i}}(t)\cdot{\boldsymbol{G}\boldsymbol{1}\boldsymbol{G}\boldsymbol{6}}_{i}(t)$$

$$\frac{d{\boldsymbol{G}\boldsymbol{1}\boldsymbol{G}\boldsymbol{6}\boldsymbol{v}\boldsymbol{2}}_{i}\left( t \right)}{dt}={c_{\boldsymbol{61}}\cdot\lambda_{{\boldsymbol{G}\boldsymbol{6}}_{i}}\left( t \right)\cdot{\boldsymbol{G}\boldsymbol{1}\boldsymbol{v}\boldsymbol{2}}_{i}\left( t \right)+c}_{\boldsymbol{16}}\cdot(1-{v2}_{\boldsymbol{G}\boldsymbol{1}})\cdot\lambda_{{\boldsymbol{G}\boldsymbol{1}}_{i}}\left( t \right)\cdot{\boldsymbol{G}\boldsymbol{6}\boldsymbol{v}\boldsymbol{2}}_{i}\left( t \right)-\left( 2\cdot r_{i}+\omega_{{v2}_{i}} \right)\cdot{\boldsymbol{G}\boldsymbol{1}\boldsymbol{G}\boldsymbol{6}\boldsymbol{v}\boldsymbol{2}}_{i}\left( t \right)+\pi_{{v2}_{i}}(t)\cdot{\boldsymbol{G}\boldsymbol{1}\boldsymbol{G}\boldsymbol{6}}_{i}(t)$$

$$\frac{d{\boldsymbol{G}\boldsymbol{1}\boldsymbol{G}\boldsymbol{7}}_{i}\left( t \right)}{dt}={c_{\boldsymbol{71}}\cdot\lambda_{{\boldsymbol{G}\boldsymbol{7}}_{i}}\left( t \right)\cdot{\boldsymbol{G}\boldsymbol{1}}_{i}\left( t \right)+c}_{\boldsymbol{17}}\cdot\lambda_{{\boldsymbol{G}\boldsymbol{1}}_{i}}\left( t \right)\cdot{\boldsymbol{G}\boldsymbol{7}}_{i}\left( t \right)-\left( 2\cdot r_{i}+\pi_{{v1}_{i}}\left( t \right)+\pi_{{v2}_{i}}\left( t \right) \right)\cdot{\boldsymbol{G}\boldsymbol{1}\boldsymbol{G}\boldsymbol{7}}_{i}\left( t \right)+\omega_{{v1}_{i}}\cdot{\boldsymbol{G}\boldsymbol{1}\boldsymbol{G}\boldsymbol{7}\boldsymbol{v}\boldsymbol{1}}_{i}\left( t \right)+\omega_{{v2}_{i}}\cdot{\boldsymbol{G}\boldsymbol{1}\boldsymbol{G}\boldsymbol{7}\boldsymbol{v}\boldsymbol{2}}_{i}\left( t \right)$$

$$\frac{d{\boldsymbol{G}\boldsymbol{1}\boldsymbol{G}\boldsymbol{7}\boldsymbol{v}\boldsymbol{1}}_{i}\left( t \right)}{dt}={c_{\boldsymbol{71}}\cdot\lambda_{{\boldsymbol{G}\boldsymbol{7}}_{i}}\left( t \right)\cdot{\boldsymbol{G}\boldsymbol{1}\boldsymbol{v}\boldsymbol{1}}_{i}\left( t \right)+c}_{\boldsymbol{17}}\cdot(1-{v1}_{\boldsymbol{G}\boldsymbol{1}})\cdot\lambda_{{\boldsymbol{G}\boldsymbol{1}}_{i}}\left( t \right)\cdot{\boldsymbol{G}\boldsymbol{7}\boldsymbol{v}\boldsymbol{1}}_{i}\left( t \right)-\left( 2\cdot r_{i}+\omega_{{v1}_{i}} \right)\cdot{\boldsymbol{G}\boldsymbol{1}\boldsymbol{G}\boldsymbol{7}\boldsymbol{v}\boldsymbol{1}}_{i}\left( t \right)+\pi_{{v1}_{i}}(t)\cdot{\boldsymbol{G}\boldsymbol{1}\boldsymbol{G}\boldsymbol{7}}_{i}(t)$$

$$\frac{d{\boldsymbol{G}\boldsymbol{1}\boldsymbol{G}\boldsymbol{7}\boldsymbol{v}\boldsymbol{2}}_{i}\left( t \right)}{dt}={c_{\boldsymbol{71}}\cdot\lambda_{{\boldsymbol{G}\boldsymbol{7}}_{i}}\left( t \right)\cdot{\boldsymbol{G}\boldsymbol{1}\boldsymbol{v}\boldsymbol{2}}_{i}\left( t \right)+c}_{\boldsymbol{17}}\cdot(1-{v2}_{\boldsymbol{G}\boldsymbol{1}})\cdot\lambda_{{\boldsymbol{G}\boldsymbol{1}}_{i}}\left( t \right)\cdot{\boldsymbol{G}\boldsymbol{7}\boldsymbol{v}\boldsymbol{2}}_{i}\left( t \right)-\left( 2\cdot r_{i}+\omega_{{v2}_{i}} \right)\cdot{\boldsymbol{G}\boldsymbol{1}\boldsymbol{G}\boldsymbol{7}\boldsymbol{v}\boldsymbol{2}}_{i}\left( t \right)+\pi_{{v2}_{i}}(t)\cdot{\boldsymbol{G}\boldsymbol{1}\boldsymbol{G}\boldsymbol{7}}_{i}(t)$$

$$\frac{d{\boldsymbol{G}\boldsymbol{2}\boldsymbol{G}\boldsymbol{3}}_{i}\left( t \right)}{dt}={c_{\boldsymbol{32}}\cdot\lambda_{{\boldsymbol{G}\boldsymbol{3}}_{i}}\left( t \right)\cdot{\boldsymbol{G}\boldsymbol{2}}_{i}\left( t \right)+c}_{\boldsymbol{23}}\cdot\lambda_{{\boldsymbol{G}\boldsymbol{2}}_{i}}\left( t \right)\cdot{\boldsymbol{G}\boldsymbol{3}}_{i}\left( t \right)-\left( 2\cdot r_{i}+\pi_{{v1}_{i}}\left( t \right)+\pi_{{v2}_{i}}\left( t \right) \right)\cdot{\boldsymbol{G}\boldsymbol{2}\boldsymbol{G}\boldsymbol{3}}_{i}\left( t \right)+\omega_{{v1}_{i}}\cdot{\boldsymbol{G}\boldsymbol{2}\boldsymbol{G}\boldsymbol{3}\boldsymbol{v}\boldsymbol{1}}_{i}\left( t \right)+\omega_{{v2}_{i}}\cdot{\boldsymbol{G}\boldsymbol{2}\boldsymbol{G}\boldsymbol{3}\boldsymbol{v}\boldsymbol{2}}_{i}\left( t \right)$$

$$\frac{d{\boldsymbol{G}\boldsymbol{2}\boldsymbol{G}\boldsymbol{3}\boldsymbol{v}\boldsymbol{1}}_{i}\left( t \right)}{dt}={c_{\boldsymbol{32}}\cdot\lambda_{{\boldsymbol{G}\boldsymbol{3}}_{i}}\left( t \right)\cdot{\boldsymbol{G}\boldsymbol{2}\boldsymbol{v}\boldsymbol{1}}_{i}\left( t \right)+c}_{\boldsymbol{23}}\cdot\lambda_{{\boldsymbol{G}\boldsymbol{2}}_{i}}\left( t \right)\cdot{\boldsymbol{G}\boldsymbol{3}\boldsymbol{v}\boldsymbol{1}}_{i}\left( t \right)-\left( 2\cdot r_{i}+\omega_{{v1}_{i}} \right)\cdot{\boldsymbol{G}\boldsymbol{2}\boldsymbol{G}\boldsymbol{3v}\boldsymbol{1}}_{i}\left( t \right)+\pi_{{v1}_{i}}(t)\cdot{\boldsymbol{G}\boldsymbol{2}\boldsymbol{G}\boldsymbol{3}}_{i}(t)$$

$$\frac{d{\boldsymbol{G}\boldsymbol{2}\boldsymbol{G}\boldsymbol{3}\boldsymbol{v}\boldsymbol{2}}_{i}\left( t \right)}{dt}={c_{\boldsymbol{32}}\cdot{(1-{v2}_{\boldsymbol{G}\boldsymbol{3}})\cdot\lambda}_{{\boldsymbol{G}\boldsymbol{3}}_{i}}\left( t \right)\cdot{\boldsymbol{G}\boldsymbol{2}\boldsymbol{v}\boldsymbol{2}}_{i}\left( t \right)+c}_{\boldsymbol{23}}\cdot(1-{v2}_{\boldsymbol{G}\boldsymbol{2}})\cdot\lambda_{{\boldsymbol{G}\boldsymbol{2}}_{i}}\left( t \right)\cdot{\boldsymbol{G}\boldsymbol{3}\boldsymbol{v}\boldsymbol{2}}_{i}\left( t \right)-\left( 2\cdot r_{i}+\omega_{{v2}_{i}} \right)\cdot{\boldsymbol{G}\boldsymbol{2}\boldsymbol{G}\boldsymbol{3}\boldsymbol{v}\boldsymbol{2}}_{i}\left( t \right)+\pi_{{v2}_{i}}(t)\cdot{\boldsymbol{G}\boldsymbol{2}\boldsymbol{G}\boldsymbol{3}}_{i}(t)$$

$$\frac{d{\boldsymbol{G}\boldsymbol{2}\boldsymbol{G}\boldsymbol{4}}_{i}(t)}{dt}={c_{\boldsymbol{42}}\cdot\lambda_{{\boldsymbol{G}\boldsymbol{4}}_{i}}\left( t \right)\cdot{\boldsymbol{G}\boldsymbol{2}}_{i}\left( t \right)+c}_{\boldsymbol{24}}\cdot\lambda_{{\boldsymbol{G}\boldsymbol{2}}_{i}}\left( t \right)\cdot{\boldsymbol{G}\boldsymbol{4}}_{i}\left( t \right)-\left( 2\cdot r_{i}+\pi_{{v1}_{i}}\left( t \right)+\pi_{{v2}_{i}}\left( t \right) \right)\cdot{\boldsymbol{G}\boldsymbol{2}\boldsymbol{G}\boldsymbol{4}}_{i}\left( t \right)+\omega_{{v1}_{i}}\cdot{\boldsymbol{G}\boldsymbol{2}\boldsymbol{G}\boldsymbol{4}\boldsymbol{v}\boldsymbol{1}}_{i}\left( t \right)+\omega_{{v2}_{i}}\cdot{\boldsymbol{G}\boldsymbol{2}\boldsymbol{G}\boldsymbol{4}\boldsymbol{v}\boldsymbol{2}}_{i}\left( t \right)$$

$$\frac{d{\boldsymbol{G}\boldsymbol{2}\boldsymbol{G}\boldsymbol{4}\boldsymbol{v}\boldsymbol{1}}_{i}(t)}{dt}={c_{\boldsymbol{42}}\cdot\lambda_{{\boldsymbol{G}\boldsymbol{4}}_{i}}\left( t \right)\cdot{\boldsymbol{G}\boldsymbol{2}\boldsymbol{v}\boldsymbol{1}}_{i}\left( t \right)+c}_{\boldsymbol{24}}\cdot\lambda_{{\boldsymbol{G}\boldsymbol{2}}_{i}}\left( t \right)\cdot{\boldsymbol{G}\boldsymbol{4}\boldsymbol{v}\boldsymbol{1}}_{i}\left( t \right)-\left( 2\cdot r_{i}+\omega_{{v1}_{i}} \right)\cdot{\boldsymbol{G}\boldsymbol{2}\boldsymbol{G}\boldsymbol{4}\boldsymbol{v}\boldsymbol{1}}_{i}\left( t \right)+\pi_{{v1}_{i}}(t)\cdot{\boldsymbol{G}\boldsymbol{2}\boldsymbol{G4}}_{i}(t)$$

$$\frac{d{\boldsymbol{G}\boldsymbol{2}\boldsymbol{G}\boldsymbol{4}\boldsymbol{v}\boldsymbol{2}}_{i}(t)}{dt}={c_{\boldsymbol{42}}\cdot\lambda_{{\boldsymbol{G}\boldsymbol{4}}_{i}}\left( t \right)\cdot{\boldsymbol{G}\boldsymbol{2}\boldsymbol{v}\boldsymbol{2}}_{i}\left( t \right)+c}_{\boldsymbol{24}}\cdot{(1-{v2}_{\boldsymbol{G}\boldsymbol{2}})\cdot\lambda}_{{\boldsymbol{G}\boldsymbol{2}}_{i}}\left( t \right)\cdot{\boldsymbol{G}\boldsymbol{4}\boldsymbol{v}\boldsymbol{2}}_{i}\left( t \right)-\left( 2\cdot r_{i}+\omega_{{v2}_{i}} \right)\cdot{\boldsymbol{G}\boldsymbol{2}\boldsymbol{G}\boldsymbol{4}\boldsymbol{v}\boldsymbol{2}}_{i}\left( t \right)+\pi_{{v2}_{i}}(t)\cdot{\boldsymbol{G}\boldsymbol{2}\boldsymbol{G}\boldsymbol{4}}_{i}(t)$$

$$\frac{d{\boldsymbol{G}\boldsymbol{2}\boldsymbol{G}\boldsymbol{5}}_{i}(t)}{dt}={c_{\boldsymbol{52}}\cdot\lambda_{{\boldsymbol{G}\boldsymbol{5}}_{i}}\left( t \right)\cdot{\boldsymbol{G}\boldsymbol{2}}_{i}\left( t \right)+c}_{\boldsymbol{25}}\cdot\lambda_{{\boldsymbol{G}\boldsymbol{2}}_{i}}\left( t \right)\cdot{\boldsymbol{G}\boldsymbol{5}}_{i}\left( t \right)-\left( 2\cdot r_{i}+\pi_{{v1}_{i}}\left( t \right)+\pi_{{v2}_{i}}\left( t \right) \right)\cdot{\boldsymbol{G}\boldsymbol{2}\boldsymbol{G}\boldsymbol{5}}_{i}\left( t \right)+\omega_{{v1}_{i}}\cdot{\boldsymbol{G}\boldsymbol{2}\boldsymbol{G}\boldsymbol{5}\boldsymbol{v}\boldsymbol{1}}_{i}\left( t \right)+\omega_{{v2}_{i}}\cdot{\boldsymbol{G}\boldsymbol{2}\boldsymbol{G}\boldsymbol{5}\boldsymbol{v}\boldsymbol{2}}_{i}\left( t \right)$$

$$\frac{d{\boldsymbol{G}\boldsymbol{2}\boldsymbol{G}\boldsymbol{5}\boldsymbol{v}\boldsymbol{1}}_{i}(t)}{dt}={c_{\boldsymbol{52}}\cdot\lambda_{{\boldsymbol{G}\boldsymbol{5}}_{i}}\left( t \right)\cdot{\boldsymbol{G}\boldsymbol{2}\boldsymbol{v}\boldsymbol{1}}_{i}\left( t \right)+c}_{\boldsymbol{25}}\cdot\lambda_{{\boldsymbol{G}\boldsymbol{2}}_{i}}\left( t \right)\cdot{\boldsymbol{G}\boldsymbol{5}\boldsymbol{v}\boldsymbol{1}}_{i}\left( t \right)-\left( 2\cdot r_{i}+\omega_{{v1}_{i}} \right)\cdot{\boldsymbol{G}\boldsymbol{2}\boldsymbol{G}\boldsymbol{5}\boldsymbol{v}\boldsymbol{1}}_{i}\left( t \right)+\pi_{{v1}_{i}}(t)\cdot{\boldsymbol{G}\boldsymbol{2}\boldsymbol{G}\boldsymbol{5}}_{i}(t)$$

$$\frac{d{\boldsymbol{G}\boldsymbol{2}\boldsymbol{G}\boldsymbol{5}\boldsymbol{v}\boldsymbol{2}}_{i}(t)}{dt}={c_{\boldsymbol{52}}\cdot\lambda_{{\boldsymbol{G}\boldsymbol{5}}_{i}}\left( t \right)\cdot{\boldsymbol{G}\boldsymbol{2}\boldsymbol{v}\boldsymbol{2}}_{i}\left( t \right)+c}_{\boldsymbol{25}}\cdot{(1-{v2}_{\boldsymbol{G}\boldsymbol{2}})\cdot\lambda}_{{\boldsymbol{G}\boldsymbol{2}}_{i}}\left( t \right)\cdot{\boldsymbol{G}\boldsymbol{5}\boldsymbol{v}\boldsymbol{2}}_{i}\left( t \right)-\left( 2\cdot r_{i}+\omega_{{v2}_{i}} \right)\cdot{\boldsymbol{G}\boldsymbol{2}\boldsymbol{G}\boldsymbol{5}\boldsymbol{v}\boldsymbol{2}}_{i}\left( t \right)+\pi_{{v2}_{i}}(t)\cdot{\boldsymbol{G}\boldsymbol{2}\boldsymbol{G}\boldsymbol{5}}_{i}(t)$$

$$\frac{d{\boldsymbol{G}\boldsymbol{2}\boldsymbol{G}\boldsymbol{6}}_{i}(t)}{dt}={c_{\boldsymbol{62}}\cdot\lambda_{{\boldsymbol{G}\boldsymbol{6}}_{i}}\left( t \right)\cdot{\boldsymbol{G}\boldsymbol{2}}_{i}\left( t \right)+c}_{\boldsymbol{26}}\cdot\lambda_{{\boldsymbol{G}\boldsymbol{2}}_{i}}\left( t \right)\cdot{\boldsymbol{G}\boldsymbol{6}}_{i}\left( t \right)-\left( 2\cdot r_{i}+\pi_{{v1}_{i}}\left( t \right)+\pi_{{v2}_{i}}\left( t \right) \right)\cdot{\boldsymbol{G}\boldsymbol{2}\boldsymbol{G}\boldsymbol{6}}_{i}\left( t \right)+\omega_{{v1}_{i}}\cdot{\boldsymbol{G}\boldsymbol{2}\boldsymbol{G6v}\boldsymbol{1}}_{i}\left( t \right)+\omega_{{v2}_{i}}\cdot{\boldsymbol{G}\boldsymbol{2}\boldsymbol{G}\boldsymbol{6}\boldsymbol{v}\boldsymbol{2}}_{i}\left( t \right)$$

$$\frac{d{\boldsymbol{G}\boldsymbol{2}\boldsymbol{G}\boldsymbol{6}\boldsymbol{v}\boldsymbol{1}}_{i}(t)}{dt}={c_{\boldsymbol{62}}\cdot\lambda_{{\boldsymbol{G}\boldsymbol{6}}_{i}}\left( t \right)\cdot{\boldsymbol{G}\boldsymbol{2}\boldsymbol{v}\boldsymbol{1}}_{i}\left( t \right)+c}_{\boldsymbol{26}}\cdot\lambda_{{\boldsymbol{G}\boldsymbol{2}}_{i}}\left( t \right)\cdot{\boldsymbol{G}\boldsymbol{6}\boldsymbol{v}\boldsymbol{1}}_{i}\left( t \right)-\left( 2\cdot r_{i}+\omega_{{v1}_{i}} \right)\cdot{\boldsymbol{G}\boldsymbol{2}\boldsymbol{G}\boldsymbol{6}\boldsymbol{v}\boldsymbol{1}}_{i}\left( t \right)+\pi_{{v1}_{i}}(t)\cdot{\boldsymbol{G}\boldsymbol{2}\boldsymbol{G}\boldsymbol{6}}_{i}(t)$$

$$\frac{d{\boldsymbol{G}\boldsymbol{2}\boldsymbol{G}\boldsymbol{6}\boldsymbol{v}\boldsymbol{2}}_{i}(t)}{dt}={c_{\boldsymbol{62}}\cdot\lambda_{{\boldsymbol{G}\boldsymbol{6}}_{i}}\left( t \right)\cdot{\boldsymbol{G}\boldsymbol{2}\boldsymbol{v}\boldsymbol{2}}_{i}\left( t \right)+c}_{\boldsymbol{26}}\cdot{(1-{v2}_{\boldsymbol{G}\boldsymbol{2}})\cdot\lambda}_{{\boldsymbol{G}\boldsymbol{2}}_{i}}\left( t \right)\cdot{\boldsymbol{G}\boldsymbol{6}\boldsymbol{v}\boldsymbol{2}}_{i}\left( t \right)-\left( 2\cdot r_{i}+\omega_{{v2}_{i}} \right)\cdot{\boldsymbol{G}\boldsymbol{2}\boldsymbol{G}\boldsymbol{6}\boldsymbol{v}\boldsymbol{2}}_{i}\left( t \right)+\pi_{{v2}_{i}}(t)\cdot{\boldsymbol{G}\boldsymbol{2}\boldsymbol{G}\boldsymbol{6}}_{i}(t)$$

$$\frac{d{\boldsymbol{G}\boldsymbol{2}\boldsymbol{G}\boldsymbol{7}}_{i}(t)}{dt}={c_{\boldsymbol{72}}\cdot\lambda_{{\boldsymbol{G}\boldsymbol{7}}_{i}}\left( t \right)\cdot{\boldsymbol{G}\boldsymbol{2}}_{i}\left( t \right)+c}_{\boldsymbol{27}}\cdot\lambda_{{\boldsymbol{G}\boldsymbol{2}}_{i}}\left( t \right)\cdot{\boldsymbol{G}\boldsymbol{7}}_{i}\left( t \right)-\left( 2\cdot r_{i}+\pi_{{v1}_{i}}\left( t \right)+\pi_{{v2}_{i}}\left( t \right) \right)\cdot{\boldsymbol{G}\boldsymbol{2}\boldsymbol{G}\boldsymbol{7}}_{i}\left( t \right)+\omega_{{v1}_{i}}\cdot{\boldsymbol{G}\boldsymbol{2}\boldsymbol{G}\boldsymbol{7}\boldsymbol{v}\boldsymbol{1}}_{i}\left( t \right)+\omega_{{v2}_{i}}\cdot{\boldsymbol{G}\boldsymbol{2}\boldsymbol{G}\boldsymbol{7}\boldsymbol{v}\boldsymbol{2}}_{i}\left( t \right)$$

$$\frac{d{\boldsymbol{G}\boldsymbol{2G}\boldsymbol{7}\boldsymbol{v}\boldsymbol{1}}_{i}(t)}{dt}={c_{\boldsymbol{72}}\cdot\lambda_{{\boldsymbol{G}\boldsymbol{7}}_{i}}\left( t \right)\cdot{\boldsymbol{G}\boldsymbol{2}\boldsymbol{v}\boldsymbol{1}}_{i}\left( t \right)+c}_{\boldsymbol{27}}\cdot\lambda_{{\boldsymbol{G}\boldsymbol{2}}_{i}}\left( t \right)\cdot{\boldsymbol{G}\boldsymbol{7}\boldsymbol{v}\boldsymbol{1}}_{i}\left( t \right)-\left( 2\cdot r_{i}+\omega_{{v1}_{i}} \right)\cdot{\boldsymbol{G}\boldsymbol{2}\boldsymbol{G}\boldsymbol{7}\boldsymbol{v}\boldsymbol{1}}_{i}\left( t \right)+\pi_{{v1}_{i}}(t)\cdot{\boldsymbol{G}\boldsymbol{2}\boldsymbol{G}\boldsymbol{7}}_{i}(t)$$

$$\frac{d{\boldsymbol{G}\boldsymbol{2}\boldsymbol{G}\boldsymbol{7}\boldsymbol{v}\boldsymbol{2}}_{i}(t)}{dt}={c_{\boldsymbol{72}}\cdot\lambda_{{\boldsymbol{G}\boldsymbol{7}}_{i}}\left( t \right)\cdot{\boldsymbol{G}\boldsymbol{2}\boldsymbol{v}\boldsymbol{2}}_{i}\left( t \right)+c}_{\boldsymbol{27}}\cdot{(1-{v2}_{\boldsymbol{G}\boldsymbol{2}})\cdot\lambda}_{{\boldsymbol{G}\boldsymbol{2}}_{i}}\left( t \right)\cdot{\boldsymbol{G}\boldsymbol{7}\boldsymbol{v}\boldsymbol{2}}_{i}\left( t \right)-\left( 2\cdot r_{i}+\omega_{{v2}_{i}} \right)\cdot{\boldsymbol{G}\boldsymbol{2}\boldsymbol{G}\boldsymbol{7}\boldsymbol{v}\boldsymbol{2}}_{i}\left( t \right)+\pi_{{v2}_{i}}(t)\cdot{\boldsymbol{G}\boldsymbol{2}\boldsymbol{G}\boldsymbol{7}}_{i}(t)$$

$$\frac{d{\boldsymbol{G}\boldsymbol{3}\boldsymbol{G}\boldsymbol{4}}_{i}(t)}{dt}={c_{\boldsymbol{43}}\cdot\lambda_{{\boldsymbol{G}\boldsymbol{4}}_{i}}\left( t \right)\cdot{\boldsymbol{G}\boldsymbol{3}}_{i}\left( t \right)+c}_{\boldsymbol{34}}\cdot\lambda_{{\boldsymbol{G}\boldsymbol{3}}_{i}}\left( t \right)\cdot{\boldsymbol{G}\boldsymbol{4}}_{i}\left( t \right)-\left( 2\cdot r_{i}+\pi_{{v1}_{i}}\left( t \right)+\pi_{{v2}_{i}}\left( t \right) \right)\cdot{\boldsymbol{G}\boldsymbol{3}\boldsymbol{G}\boldsymbol{4}}_{i}(t)+\omega_{{v1}_{i}}\cdot{\boldsymbol{G}\boldsymbol{3}\boldsymbol{G}\boldsymbol{4}\boldsymbol{v}\boldsymbol{1}}_{i}\left( t \right)+\omega_{{v2}_{i}}\cdot{\boldsymbol{G}\boldsymbol{3}\boldsymbol{G}\boldsymbol{4}\boldsymbol{v}\boldsymbol{2}}_{i}\left( t \right)$$

$$\frac{d{\boldsymbol{G}\boldsymbol{3}\boldsymbol{G}\boldsymbol{4}\boldsymbol{v}\boldsymbol{1}}_{i}(t)}{dt}={c_{\boldsymbol{43}}\cdot\lambda_{{\boldsymbol{G}\boldsymbol{4}}_{i}}\left( t \right)\cdot{\boldsymbol{G3}\boldsymbol{v}\boldsymbol{1}}_{i}\left( t \right)+c}_{\boldsymbol{34}}\cdot\lambda_{{\boldsymbol{G}\boldsymbol{3}}_{i}}\left( t \right)\cdot{\boldsymbol{G}\boldsymbol{4}\boldsymbol{v}\boldsymbol{1}}_{i}\left( t \right)-\left( 2\cdot r_{i}+\omega_{{v1}_{i}} \right)\cdot{\boldsymbol{G}\boldsymbol{3}\boldsymbol{G}\boldsymbol{4}\boldsymbol{v}\boldsymbol{1}}_{i}(t)+\pi_{{v1}_{i}}(t)\cdot{\boldsymbol{G}\boldsymbol{3}\boldsymbol{G}\boldsymbol{4}}_{i}(t)$$

$$\frac{d{\boldsymbol{G}\boldsymbol{3}\boldsymbol{G}\boldsymbol{4}\boldsymbol{v}\boldsymbol{2}}_{i}(t)}{dt}={c_{\boldsymbol{43}}\cdot\lambda_{{\boldsymbol{G}\boldsymbol{4}}_{i}}\left( t \right)\cdot{\boldsymbol{G}\boldsymbol{3}\boldsymbol{v}\boldsymbol{2}}_{i}\left( t \right)+c}_{\boldsymbol{34}}\cdot{(1-{v2}_{\boldsymbol{G}\boldsymbol{3}})\cdot\lambda}_{{\boldsymbol{G}\boldsymbol{3}}_{i}}\left( t \right)\cdot{\boldsymbol{G}\boldsymbol{4}\boldsymbol{v}\boldsymbol{2}}_{i}\left( t \right)-\left( 2\cdot r_{i}+\omega_{{v2}_{i}} \right)\cdot{\boldsymbol{G}\boldsymbol{3}\boldsymbol{G}\boldsymbol{4}\boldsymbol{v}\boldsymbol{2}}_{i}(t)+\pi_{{v2}_{i}}(t)\cdot{\boldsymbol{G}\boldsymbol{3}\boldsymbol{G4}}_{i}(t)$$

$$\frac{d{\boldsymbol{G}\boldsymbol{3}\boldsymbol{G}\boldsymbol{5}}_{i}(t)}{dt}={c_{\boldsymbol{53}}\cdot\lambda_{{\boldsymbol{G}\boldsymbol{5}}_{i}}\left( t \right)\cdot{\boldsymbol{G}\boldsymbol{3}}_{i}\left( t \right)+c}_{\boldsymbol{35}}\cdot\lambda_{{\boldsymbol{G}\boldsymbol{3}}_{i}}\left( t \right)\cdot{\boldsymbol{G}\boldsymbol{5}}_{i}\left( t \right)-\left( 2\cdot r_{i}+\pi_{{v1}_{i}}\left( t \right)+\pi_{{v2}_{i}}\left( t \right) \right)\cdot{\boldsymbol{G}\boldsymbol{3}\boldsymbol{G}\boldsymbol{5}}_{i}(t)+\omega_{{v1}_{i}}\cdot{\boldsymbol{G}\boldsymbol{3}\boldsymbol{G}\boldsymbol{5}\boldsymbol{v}\boldsymbol{1}}_{i}\left( t \right)+\omega_{{v2}_{i}}\cdot{\boldsymbol{G}\boldsymbol{3}\boldsymbol{G}\boldsymbol{5}\boldsymbol{v}\boldsymbol{2}}_{i}\left( t \right)$$

$$\frac{d{\boldsymbol{G}\boldsymbol{3}\boldsymbol{G}\boldsymbol{5}\boldsymbol{v}\boldsymbol{1}}_{i}(t)}{dt}={c_{\boldsymbol{53}}\cdot\lambda_{{\boldsymbol{G}\boldsymbol{5}}_{i}}\left( t \right)\cdot{\boldsymbol{G}\boldsymbol{3}\boldsymbol{v}\boldsymbol{1}}_{i}\left( t \right)+c}_{\boldsymbol{35}}\cdot\lambda_{{\boldsymbol{G}\boldsymbol{3}}_{i}}\left( t \right)\cdot{\boldsymbol{G}\boldsymbol{5}\boldsymbol{v1}}_{i}\left( t \right)-\left( 2\cdot r_{i}+\omega_{{v1}_{i}} \right)\cdot{\boldsymbol{G}\boldsymbol{3}\boldsymbol{G}\boldsymbol{5}\boldsymbol{v}\boldsymbol{1}}_{i}(t)+\pi_{{v1}_{i}}(t)\cdot{\boldsymbol{G}\boldsymbol{3}\boldsymbol{G}\boldsymbol{5}}_{i}(t)$$

$$\frac{d{\boldsymbol{G}\boldsymbol{3}\boldsymbol{G}\boldsymbol{5}\boldsymbol{v}\boldsymbol{2}}_{i}(t)}{dt}={c_{\boldsymbol{53}}\cdot\lambda_{{\boldsymbol{G}\boldsymbol{5}}_{i}}\left( t \right)\cdot{\boldsymbol{G}\boldsymbol{3}\boldsymbol{v}\boldsymbol{2}}_{i}\left( t \right)+c}_{\boldsymbol{35}}\cdot{(1-{v2}_{\boldsymbol{G}\boldsymbol{3}})\cdot\lambda}_{{\boldsymbol{G}\boldsymbol{3}}_{i}}\left( t \right)\cdot{\boldsymbol{G}\boldsymbol{5}\boldsymbol{v}\boldsymbol{2}}_{i}\left( t \right)-\left( 2\cdot r_{i}+\omega_{{v2}_{i}} \right)\cdot{\boldsymbol{G}\boldsymbol{3}\boldsymbol{G}\boldsymbol{5}\boldsymbol{v}\boldsymbol{2}}_{i}(t)+\pi_{{v2}_{i}}(t)\cdot{\boldsymbol{G}\boldsymbol{3}\boldsymbol{G}\boldsymbol{5}}_{i}(t)$$

$$\frac{d{\boldsymbol{G}\boldsymbol{3}\boldsymbol{G}\boldsymbol{6}}_{i}(t)}{dt}={c_{\boldsymbol{63}}\cdot\lambda_{{\boldsymbol{G}\boldsymbol{6}}_{i}}\left( t \right)\cdot{\boldsymbol{G}\boldsymbol{3}}_{i}\left( t \right)+c}_{\boldsymbol{36}}\cdot\lambda_{{\boldsymbol{G}\boldsymbol{3}}_{i}}\left( t \right)\cdot{\boldsymbol{G}\boldsymbol{6}}_{i}\left( t \right)-\left( 2\cdot r_{i}+\pi_{{v1}_{i}}\left( t \right)+\pi_{{v2}_{i}}\left( t \right) \right)\cdot{\boldsymbol{G}\boldsymbol{3}\boldsymbol{G}\boldsymbol{6}}_{i}(t)+\omega_{{v1}_{i}}\cdot{\boldsymbol{G}\boldsymbol{3}\boldsymbol{G}\boldsymbol{6}\boldsymbol{v}\boldsymbol{1}}_{i}\left( t \right)+\omega_{{v2}_{i}}\cdot{\boldsymbol{G}\boldsymbol{3}\boldsymbol{G}\boldsymbol{6}\boldsymbol{v}\boldsymbol{2}}_{i}\left( t \right)$$

$$\frac{d{\boldsymbol{G}\boldsymbol{3}\boldsymbol{G}\boldsymbol{6}\boldsymbol{v}\boldsymbol{1}}_{i}(t)}{dt}={c_{\boldsymbol{63}}\cdot\lambda_{{\boldsymbol{G}\boldsymbol{6}}_{i}}\left( t \right)\cdot{\boldsymbol{G}\boldsymbol{3}\boldsymbol{v}\boldsymbol{1}}_{i}\left( t \right)+c}_{\boldsymbol{36}}\cdot\lambda_{{\boldsymbol{G}\boldsymbol{3}}_{i}}\left( t \right)\cdot{\boldsymbol{G}\boldsymbol{6}\boldsymbol{v}\boldsymbol{1}}_{i}\left( t \right)-\left( 2\cdot r_{i}+\omega_{{v1}_{i}} \right)\cdot{\boldsymbol{G}\boldsymbol{3}\boldsymbol{G}\boldsymbol{6}\boldsymbol{v1}}_{i}(t)+\pi_{{v1}_{i}}(t)\cdot{\boldsymbol{G}\boldsymbol{3}\boldsymbol{G}\boldsymbol{6}}_{i}(t)$$

$$\frac{d{\boldsymbol{G}\boldsymbol{3}\boldsymbol{G}\boldsymbol{6}\boldsymbol{v}\boldsymbol{2}}_{i}(t)}{dt}={c_{\boldsymbol{63}}\cdot\lambda_{{\boldsymbol{G}\boldsymbol{6}}_{i}}\left( t \right)\cdot{\boldsymbol{G}\boldsymbol{3}\boldsymbol{v}\boldsymbol{2}}_{i}\left( t \right)+c}_{\boldsymbol{36}}\cdot{(1-{v2}_{\boldsymbol{G}\boldsymbol{3}})\cdot\lambda}_{{\boldsymbol{G}\boldsymbol{3}}_{i}}\left( t \right)\cdot{\boldsymbol{G}\boldsymbol{6}\boldsymbol{v}\boldsymbol{2}}_{i}\left( t \right)-\left( 2\cdot r_{i}+\omega_{{v2}_{i}} \right)\cdot{\boldsymbol{G}\boldsymbol{3}\boldsymbol{G}\boldsymbol{6}\boldsymbol{v}\boldsymbol{2}}_{i}(t)+\pi_{{v2}_{i}}(t)\cdot{\boldsymbol{G}\boldsymbol{3}\boldsymbol{G}\boldsymbol{6}}_{i}(t)$$

$$\frac{d{\boldsymbol{G}\boldsymbol{3}\boldsymbol{G}\boldsymbol{7}}_{i}(t)}{dt}={c_{\boldsymbol{73}}\cdot\lambda_{{\boldsymbol{G}\boldsymbol{7}}_{i}}\left( t \right)\cdot{\boldsymbol{G}\boldsymbol{3}}_{i}\left( t \right)+c}_{\boldsymbol{37}}\cdot\lambda_{{\boldsymbol{G}\boldsymbol{3}}_{i}}\left( t \right)\cdot{\boldsymbol{G}\boldsymbol{7}}_{i}\left( t \right)-\left( 2\cdot r_{i}+\pi_{{v1}_{i}}\left( t \right)+\pi_{{v2}_{i}}\left( t \right) \right)\cdot{\boldsymbol{G}\boldsymbol{3}\boldsymbol{G}\boldsymbol{7}}_{i}(t)+\omega_{{v1}_{i}}\cdot{\boldsymbol{G}\boldsymbol{3}\boldsymbol{G}\boldsymbol{7}\boldsymbol{v}\boldsymbol{1}}_{i}\left( t \right)+\omega_{{v2}_{i}}\cdot{\boldsymbol{G}\boldsymbol{3}\boldsymbol{G}\boldsymbol{7}\boldsymbol{v}\boldsymbol{2}}_{i}\left( t \right)$$

$$\frac{d{\boldsymbol{G}\boldsymbol{3}\boldsymbol{G}\boldsymbol{7}\boldsymbol{v}\boldsymbol{1}}_{i}(t)}{dt}={c_{\boldsymbol{73}}\cdot\lambda_{{\boldsymbol{G}\boldsymbol{7}}_{i}}\left( t \right)\cdot{\boldsymbol{G}\boldsymbol{3}\boldsymbol{v}\boldsymbol{1}}_{i}\left( t \right)+c}_{\boldsymbol{37}}\cdot\lambda_{{\boldsymbol{G}\boldsymbol{3}}_{i}}\left( t \right)\cdot{\boldsymbol{G}\boldsymbol{7}\boldsymbol{v}\boldsymbol{1}}_{i}\left( t \right)-\left( 2\cdot r_{i}+\omega_{{v1}_{i}} \right)\cdot{\boldsymbol{G}\boldsymbol{3}\boldsymbol{G}\boldsymbol{7}\boldsymbol{v}\boldsymbol{1}}_{i}(t)+\pi_{{v1}_{i}}(t)\cdot{\boldsymbol{G}\boldsymbol{3}\boldsymbol{G}\boldsymbol{7}}_{i}(t)$$

$$\frac{d{\boldsymbol{G}\boldsymbol{3}\boldsymbol{G}\boldsymbol{7}\boldsymbol{v}\boldsymbol{2}}_{i}(t)}{dt}={c_{\boldsymbol{73}}\cdot\lambda_{{\boldsymbol{G}\boldsymbol{7}}_{i}}\left( t \right)\cdot{\boldsymbol{G}\boldsymbol{3}\boldsymbol{v}\boldsymbol{2}}_{i}\left( t \right)+c}_{\boldsymbol{37}}\cdot{(1-{v2}_{\boldsymbol{G}\boldsymbol{3}})\cdot\lambda}_{{\boldsymbol{G}\boldsymbol{3}}_{i}}\left( t \right)\cdot{\boldsymbol{G}\boldsymbol{7}\boldsymbol{v}\boldsymbol{2}}_{i}\left( t \right)-\left( 2\cdot r_{i}+\omega_{{v2}_{i}} \right)\cdot{\boldsymbol{G}\boldsymbol{3}\boldsymbol{G}\boldsymbol{7}\boldsymbol{v}\boldsymbol{2}}_{i}(t)+\pi_{{v2}_{i}}(t)\cdot{\boldsymbol{G}\boldsymbol{3}\boldsymbol{G}\boldsymbol{7}}_{i}(t)$$

$$\frac{d{\boldsymbol{G}\boldsymbol{4}\boldsymbol{G}\boldsymbol{5}}_{i}(t)}{dt}={c_{\boldsymbol{54}}\cdot\lambda_{{\boldsymbol{G}\boldsymbol{5}}_{i}}\left( t \right)\cdot{\boldsymbol{G}\boldsymbol{4}}_{i}\left( t \right)+c}_{\boldsymbol{45}}\cdot\lambda_{{\boldsymbol{G}\boldsymbol{4}}_{i}}\left( t \right)\cdot{\boldsymbol{G}\boldsymbol{5}}_{i}\left( t \right)-\left( 2\cdot r_{i}+\pi_{{v1}_{i}}\left( t \right)+\pi_{{v2}_{i}}\left( t \right) \right)\cdot{\boldsymbol{G}\boldsymbol{4}\boldsymbol{G}\boldsymbol{5}}_{i}(t)+\omega_{{v1}_{i}}\cdot{\boldsymbol{G}\boldsymbol{4}\boldsymbol{G}\boldsymbol{5}\boldsymbol{v}\boldsymbol{1}}_{i}\left( t \right)+\omega_{{v2}_{i}}\cdot{\boldsymbol{G}\boldsymbol{4}\boldsymbol{G}\boldsymbol{5}\boldsymbol{v}\boldsymbol{2}}_{i}\left( t \right)$$

$$\frac{d{\boldsymbol{G}\boldsymbol{4}\boldsymbol{G}\boldsymbol{5}\boldsymbol{v}\boldsymbol{1}}_{i}(t)}{dt}={c_{\boldsymbol{54}}\cdot\lambda_{{\boldsymbol{G}\boldsymbol{5}}_{i}}\left( t \right)\cdot{\boldsymbol{G}\boldsymbol{4}\boldsymbol{v}\boldsymbol{1}}_{i}\left( t \right)+c}_{\boldsymbol{45}}\cdot\lambda_{{\boldsymbol{G}\boldsymbol{4}}_{i}}\left( t \right)\cdot{\boldsymbol{G}\boldsymbol{5}\boldsymbol{v}\boldsymbol{1}}_{i}\left( t \right)-\left( 2\cdot r_{i}+\omega_{{v1}_{i}} \right)\cdot{\boldsymbol{G}\boldsymbol{4}\boldsymbol{G}\boldsymbol{5}\boldsymbol{v}\boldsymbol{1}}_{i}(t)+\pi_{{v1}_{i}}(t)\cdot{\boldsymbol{G}\boldsymbol{4}\boldsymbol{G}\boldsymbol{5}}_{i}(t)$$

$$\frac{d{\boldsymbol{G}\boldsymbol{4}\boldsymbol{G}\boldsymbol{5}\boldsymbol{v}\boldsymbol{2}}_{i}(t)}{dt}={c_{\boldsymbol{54}}\cdot\lambda_{{\boldsymbol{G}\boldsymbol{5}}_{i}}\left( t \right)\cdot{\boldsymbol{G}\boldsymbol{4}\boldsymbol{v}\boldsymbol{2}}_{i}\left( t \right)+c}_{\boldsymbol{45}}\cdot\lambda_{{\boldsymbol{G}\boldsymbol{4}}_{i}}\left( t \right)\cdot{\boldsymbol{G}\boldsymbol{5}\boldsymbol{v}\boldsymbol{2}}_{i}\left( t \right)-\left( 2\cdot r_{i}+\omega_{{v2}_{i}} \right)\cdot{\boldsymbol{G}\boldsymbol{4}\boldsymbol{G}\boldsymbol{5}\boldsymbol{v}\boldsymbol{2}}_{i}(t)+\pi_{{v2}_{i}}(t)\cdot{\boldsymbol{G}\boldsymbol{4}\boldsymbol{G}\boldsymbol{5}}_{i}(t)$$

$$\frac{d{\boldsymbol{G}\boldsymbol{4}\boldsymbol{G}\boldsymbol{6}}_{i}(t)}{dt}={c_{\boldsymbol{64}}\cdot\lambda_{{\boldsymbol{G}\boldsymbol{6}}_{i}}\left( t \right)\cdot{\boldsymbol{G}\boldsymbol{4}}_{i}\left( t \right)+c}_{\boldsymbol{46}}\cdot\lambda_{{\boldsymbol{G}\boldsymbol{4}}_{i}}\left( t \right)\cdot{\boldsymbol{G}\boldsymbol{6}}_{i}\left( t \right)-\left( 2\cdot r_{i}+\pi_{{v1}_{i}}\left( t \right)+\pi_{{v2}_{i}}\left( t \right) \right)\cdot{\boldsymbol{G}\boldsymbol{4}\boldsymbol{G}\boldsymbol{6}}_{i}(t)+\omega_{{v1}_{i}}\cdot{\boldsymbol{G}\boldsymbol{4}\boldsymbol{G}\boldsymbol{6}\boldsymbol{v}\boldsymbol{1}}_{i}\left( t \right)+\omega_{{v2}_{i}}\cdot{\boldsymbol{G}\boldsymbol{4}\boldsymbol{G}\boldsymbol{6}\boldsymbol{v}\boldsymbol{2}}_{i}\left( t \right)$$

$$\frac{d{\boldsymbol{G}\boldsymbol{4}\boldsymbol{G}\boldsymbol{6}\boldsymbol{v}\boldsymbol{1}}_{i}(t)}{dt}={c_{\boldsymbol{64}}\cdot\lambda_{{\boldsymbol{G}\boldsymbol{6}}_{i}}\left( t \right)\cdot{\boldsymbol{G}\boldsymbol{4}\boldsymbol{v}\boldsymbol{1}}_{i}\left( t \right)+c}_{\boldsymbol{46}}\cdot\lambda_{{\boldsymbol{G}\boldsymbol{4}}_{i}}\left( t \right)\cdot{\boldsymbol{G}\boldsymbol{6}\boldsymbol{v}\boldsymbol{1}}_{i}\left( t \right)-\left( 2\cdot r_{i}+\omega_{{v1}_{i}} \right)\cdot{\boldsymbol{G}\boldsymbol{4}\boldsymbol{G}\boldsymbol{6}\boldsymbol{v}\boldsymbol{1}}_{i}(t)+\pi_{{v1}_{i}}(t)\cdot{\boldsymbol{G}\boldsymbol{4}\boldsymbol{G}\boldsymbol{6}}_{i}(t)$$

$$\frac{d{\boldsymbol{G}\boldsymbol{4}\boldsymbol{G}\boldsymbol{6}\boldsymbol{v}\boldsymbol{2}}_{i}(t)}{dt}={c_{\boldsymbol{64}}\cdot\lambda_{{\boldsymbol{G}\boldsymbol{6}}_{i}}\left( t \right)\cdot{\boldsymbol{G}\boldsymbol{4}\boldsymbol{v}\boldsymbol{2}}_{i}\left( t \right)+c}_{\boldsymbol{46}}\cdot\lambda_{{\boldsymbol{G}\boldsymbol{4}}_{i}}\left( t \right)\cdot{\boldsymbol{G}\boldsymbol{6}\boldsymbol{v2}}_{i}\left( t \right)-\left( 2\cdot r_{i}+\omega_{{v2}_{i}} \right)\cdot{\boldsymbol{G}\boldsymbol{4}\boldsymbol{G}\boldsymbol{6}\boldsymbol{v}\boldsymbol{2}}_{i}(t)+\pi_{{v2}_{i}}(t)\cdot{\boldsymbol{G}\boldsymbol{4}\boldsymbol{G}\boldsymbol{6}}_{i}(t)$$

$$\frac{d{\boldsymbol{G}\boldsymbol{4}\boldsymbol{G}\boldsymbol{7}}_{i}(t)}{dt}={c_{\boldsymbol{74}}\cdot\lambda_{{\boldsymbol{G}\boldsymbol{7}}_{i}}\left( t \right)\cdot{\boldsymbol{G}\boldsymbol{4}}_{i}\left( t \right)+c}_{\boldsymbol{47}}\cdot\lambda_{{\boldsymbol{G}\boldsymbol{4}}_{i}}\left( t \right)\cdot{\boldsymbol{G}\boldsymbol{7}}_{i}\left( t \right)-\left( 2\cdot r_{i}+\pi_{{v1}_{i}}\left( t \right)+\pi_{{v2}_{i}}\left( t \right) \right)\cdot{\boldsymbol{G}\boldsymbol{4}\boldsymbol{G}\boldsymbol{7}}_{i}(t)+\omega_{{v1}_{i}}\cdot{\boldsymbol{G}\boldsymbol{4}\boldsymbol{G}\boldsymbol{7}\boldsymbol{v}\boldsymbol{1}}_{i}\left( t \right)+\omega_{{v2}_{i}}\cdot{\boldsymbol{G}\boldsymbol{4}\boldsymbol{G}\boldsymbol{7}\boldsymbol{v}\boldsymbol{2}}_{i}\left( t \right)$$

$$\frac{d{\boldsymbol{G}\boldsymbol{4}\boldsymbol{G}\boldsymbol{7}\boldsymbol{v}\boldsymbol{1}}_{i}(t)}{dt}={c_{\boldsymbol{74}}\cdot\lambda_{{\boldsymbol{G}\boldsymbol{7}}_{i}}\left( t \right)\cdot{\boldsymbol{G}\boldsymbol{4}\boldsymbol{v}\boldsymbol{1}}_{i}\left( t \right)+c}_{\boldsymbol{47}}\cdot\lambda_{{\boldsymbol{G}\boldsymbol{4}}_{i}}\left( t \right)\cdot{\boldsymbol{G}\boldsymbol{7}\boldsymbol{v}\boldsymbol{1}}_{i}\left( t \right)-\left( 2\cdot r_{i}+\omega_{{v1}_{i}} \right)\cdot{\boldsymbol{G}\boldsymbol{4}\boldsymbol{G}\boldsymbol{7}\boldsymbol{v}\boldsymbol{1}}_{i}(t)+\pi_{{v1}_{i}}(t)\cdot{\boldsymbol{G}\boldsymbol{4}\boldsymbol{G}\boldsymbol{7}}_{i}(t)$$

$$\frac{d{\boldsymbol{G}\boldsymbol{4}\boldsymbol{G}\boldsymbol{7}\boldsymbol{v}\boldsymbol{2}}_{i}(t)}{dt}={c_{\boldsymbol{74}}\cdot\lambda_{{\boldsymbol{G}\boldsymbol{7}}_{i}}\left( t \right)\cdot{\boldsymbol{G}\boldsymbol{4}\boldsymbol{v}\boldsymbol{2}}_{i}\left( t \right)+c}_{\boldsymbol{47}}\cdot\lambda_{{\boldsymbol{G}\boldsymbol{4}}_{i}}\left( t \right)\cdot{\boldsymbol{G}\boldsymbol{7}\boldsymbol{v}\boldsymbol{2}}_{i}\left( t \right)-\left( 2\cdot r_{i}+\omega_{{v2}_{i}} \right)\cdot{\boldsymbol{G}\boldsymbol{4}\boldsymbol{G}\boldsymbol{7}\boldsymbol{v}\boldsymbol{2}}_{i}(t)+\pi_{{v2}_{i}}(t)\cdot{\boldsymbol{G}\boldsymbol{4}\boldsymbol{G}\boldsymbol{7}}_{i}(t)$$

$$\frac{d{\boldsymbol{G}\boldsymbol{5}\boldsymbol{G}\boldsymbol{6}}_{i}(t)}{dt}={c_{\boldsymbol{65}}\cdot\lambda_{{\boldsymbol{G}\boldsymbol{6}}_{i}}\left( t \right)\cdot{\boldsymbol{G}\boldsymbol{5}}_{i}\left( t \right)+c}_{\boldsymbol{56}}\cdot\lambda_{{\boldsymbol{G}\boldsymbol{5}}_{i}}\left( t \right)\cdot{\boldsymbol{G}\boldsymbol{6}}_{i}\left( t \right)-\left( 2\cdot r_{i}+\pi_{{v1}_{i}}\left( t \right)+\pi_{{v2}_{i}}\left( t \right) \right)\cdot{\boldsymbol{G}\boldsymbol{5}\boldsymbol{G}\boldsymbol{6}}_{i}(t)+\omega_{{v1}_{i}}\cdot{\boldsymbol{G}\boldsymbol{5}\boldsymbol{G}\boldsymbol{6}\boldsymbol{v}\boldsymbol{1}}_{i}\left( t \right)+\omega_{{v2}_{i}}\cdot{\boldsymbol{G}\boldsymbol{5}\boldsymbol{G}\boldsymbol{6}\boldsymbol{v}\boldsymbol{2}}_{i}\left( t \right)$$

$$\frac{d{\boldsymbol{G}\boldsymbol{5}\boldsymbol{G}\boldsymbol{6}\boldsymbol{v}\boldsymbol{1}}_{i}(t)}{dt}={c_{\boldsymbol{65}}\cdot\lambda_{{\boldsymbol{G}\boldsymbol{6}}_{i}}\left( t \right)\cdot{\boldsymbol{G}\boldsymbol{5}\boldsymbol{v}\boldsymbol{1}}_{i}\left( t \right)+c}_{\boldsymbol{56}}\cdot\lambda_{\boldsymbol{G5}_{i}}\left( t \right)\cdot{\boldsymbol{G}\boldsymbol{6}\boldsymbol{v}\boldsymbol{1}}_{i}\left( t \right)-\left( 2\cdot r_{i}+\omega_{{v1}_{i}} \right)\cdot{\boldsymbol{G}\boldsymbol{5}\boldsymbol{G}\boldsymbol{6}\boldsymbol{v}\boldsymbol{1}}_{i}(t)+\pi_{{v1}_{i}}(t)\cdot{\boldsymbol{G}\boldsymbol{5}\boldsymbol{G}\boldsymbol{6}}_{i}(t)$$

$$\frac{d{\boldsymbol{G}\boldsymbol{5}\boldsymbol{G}\boldsymbol{6}\boldsymbol{v}\boldsymbol{2}}_{i}(t)}{dt}={c_{\boldsymbol{65}}\cdot\lambda_{{\boldsymbol{G}\boldsymbol{6}}_{i}}\left( t \right)\cdot{\boldsymbol{G}\boldsymbol{5}\boldsymbol{v}\boldsymbol{2}}_{i}\left( t \right)+c}_{\boldsymbol{56}}\cdot\lambda_{{\boldsymbol{G}\boldsymbol{5}}_{i}}\left( t \right)\cdot{\boldsymbol{G}\boldsymbol{6}\boldsymbol{v}\boldsymbol{2}}_{i}\left( t \right)-\left( 2\cdot r_{i}+\omega_{{v2}_{i}} \right)\cdot{\boldsymbol{G}\boldsymbol{5}\boldsymbol{G}\boldsymbol{6}\boldsymbol{v}\boldsymbol{2}}_{i}(t)+\pi_{{v2}_{i}}(t)\cdot{\boldsymbol{G}\boldsymbol{5}\boldsymbol{G}\boldsymbol{6}}_{i}(t)$$

$$\frac{d{\boldsymbol{G}\boldsymbol{5}\boldsymbol{G}\boldsymbol{7}}_{i}(t)}{dt}={c_{\boldsymbol{75}}\cdot\lambda_{{\boldsymbol{G}\boldsymbol{7}}_{i}}\left( t \right)\cdot{\boldsymbol{G}\boldsymbol{5}}_{i}\left( t \right)+c}_{\boldsymbol{57}}\cdot\lambda_{{\boldsymbol{G}\boldsymbol{5}}_{i}}\left( t \right)\cdot{\boldsymbol{G}\boldsymbol{7}}_{i}\left( t \right)-\left( 2\cdot r_{i}+\pi_{{v1}_{i}}\left( t \right)+\pi_{{v2}_{i}}\left( t \right) \right)\cdot{\boldsymbol{G}\boldsymbol{5}\boldsymbol{G}\boldsymbol{7}}_{i}(t)+\omega_{{v1}_{i}}\cdot{\boldsymbol{G}\boldsymbol{5}\boldsymbol{G}\boldsymbol{7}\boldsymbol{v}\boldsymbol{1}}_{i}\left( t \right)+\omega_{{v2}_{i}}\cdot{\boldsymbol{G}\boldsymbol{5}\boldsymbol{G}\boldsymbol{7}\boldsymbol{v}\boldsymbol{2}}_{i}\left( t \right)$$

$$\frac{d{\boldsymbol{G}\boldsymbol{5}\boldsymbol{G}\boldsymbol{7}\boldsymbol{v}\boldsymbol{1}}_{i}(t)}{dt}={c_{\boldsymbol{75}}\cdot\lambda_{{\boldsymbol{G}\boldsymbol{7}}_{i}}\left( t \right)\cdot{\boldsymbol{G}\boldsymbol{5}\boldsymbol{v}\boldsymbol{1}}_{i}\left( t \right)+c}_{\boldsymbol{57}}\cdot\lambda_{{\boldsymbol{G}\boldsymbol{5}}_{i}}\left( t \right)\cdot{\boldsymbol{G}\boldsymbol{7}\boldsymbol{v}\boldsymbol{1}}_{i}\left( t \right)-\left( 2\cdot r_{i}+\omega_{{v1}_{i}} \right)\cdot{\boldsymbol{G}\boldsymbol{5}\boldsymbol{G}\boldsymbol{7}\boldsymbol{v1}}_{i}(t)+\pi_{{v1}_{i}}(t)\cdot{\boldsymbol{G}\boldsymbol{5}\boldsymbol{G}\boldsymbol{7}}_{i}(t)$$

$$\frac{d{\boldsymbol{G}\boldsymbol{5}\boldsymbol{G}\boldsymbol{7}\boldsymbol{v}\boldsymbol{2}}_{i}(t)}{dt}={c_{\boldsymbol{75}}\cdot\lambda_{{\boldsymbol{G}\boldsymbol{7}}_{i}}\left( t \right)\cdot{\boldsymbol{G}\boldsymbol{5}\boldsymbol{v}\boldsymbol{2}}_{i}\left( t \right)+c}_{\boldsymbol{57}}\cdot\lambda_{{\boldsymbol{G}\boldsymbol{5}}_{i}}\left( t \right)\cdot{\boldsymbol{G}\boldsymbol{7}\boldsymbol{v}\boldsymbol{2}}_{i}\left( t \right)-\left( 2\cdot r_{i}+\omega_{{v2}_{i}} \right)\cdot{\boldsymbol{G}\boldsymbol{5}\boldsymbol{G}\boldsymbol{7}\boldsymbol{v}\boldsymbol{2}}_{i}(t)+\pi_{{v2}_{i}}(t)\cdot{\boldsymbol{G}\boldsymbol{5}\boldsymbol{G}\boldsymbol{7}}_{i}(t)$$

$$\frac{d{\boldsymbol{G}\boldsymbol{6}\boldsymbol{G}\boldsymbol{7}}_{i}(t)}{dt}={c_{\boldsymbol{76}}\cdot\lambda_{{\boldsymbol{G}\boldsymbol{7}}_{i}}\left( t \right)\cdot{\boldsymbol{G}\boldsymbol{6}}_{i}\left( t \right)+c}_{\boldsymbol{67}}\cdot\lambda_{{\boldsymbol{G}\boldsymbol{6}}_{i}}\left( t \right)\cdot{\boldsymbol{G}\boldsymbol{7}}_{i}\left( t \right)-\left( 2\cdot r_{i}+\pi_{{v1}_{i}}\left( t \right)+\pi_{{v2}_{i}}\left( t \right) \right)\cdot{\boldsymbol{G}\boldsymbol{6}\boldsymbol{G}\boldsymbol{7}}_{i}(t)+\omega_{{v1}_{i}}\cdot{\boldsymbol{G}\boldsymbol{6}\boldsymbol{G}\boldsymbol{7}\boldsymbol{v}\boldsymbol{1}}_{i}\left( t \right)+\omega_{{v2}_{i}}\cdot{\boldsymbol{G}\boldsymbol{6}\boldsymbol{G}\boldsymbol{7}\boldsymbol{v}\boldsymbol{2}}_{i}\left( t \right)$$

$$\frac{d{\boldsymbol{G}\boldsymbol{6}\boldsymbol{G}\boldsymbol{7}\boldsymbol{v}\boldsymbol{1}}_{i}(t)}{dt}={c_{\boldsymbol{76}}\cdot\lambda_{{\boldsymbol{G}\boldsymbol{7}}_{i}}\left( t \right)\cdot{\boldsymbol{G}\boldsymbol{6}\boldsymbol{v}\boldsymbol{1}}_{i}\left( t \right)+c}_{\boldsymbol{67}}\cdot\lambda_{{\boldsymbol{G}\boldsymbol{6}}_{i}}\left( t \right)\cdot{\boldsymbol{G}\boldsymbol{7}\boldsymbol{v}\boldsymbol{1}}_{i}\left( t \right)-\left( 2\cdot r_{i}+\omega_{{v1}_{i}} \right)\cdot{\boldsymbol{G}\boldsymbol{6}\boldsymbol{G}\boldsymbol{7}\boldsymbol{v}\boldsymbol{1}}_{i}(t)+\pi_{{v1}_{i}}(t)\cdot{\boldsymbol{G}\boldsymbol{6}\boldsymbol{G}\boldsymbol{7}}_{i}(t)$$

$$\frac{d{\boldsymbol{G}\boldsymbol{6}\boldsymbol{G}\boldsymbol{7v2}}_{i}(t)}{dt}={c_{\boldsymbol{76}}\cdot\lambda_{{\boldsymbol{G}\boldsymbol{7}}_{i}}\left( t \right)\cdot{\boldsymbol{G}\boldsymbol{6}\boldsymbol{v}\boldsymbol{2}}_{i}\left( t \right)+c}_{\boldsymbol{67}}\cdot\lambda_{{\boldsymbol{G}\boldsymbol{6}}_{i}}\left( t \right)\cdot{\boldsymbol{G}\boldsymbol{7}\boldsymbol{v}\boldsymbol{2}}_{i}\left( t \right)-\left( 2\cdot r_{i}+\omega_{{v2}_{i}} \right)\cdot{\boldsymbol{G}\boldsymbol{6}\boldsymbol{G}\boldsymbol{7}\boldsymbol{v}\boldsymbol{2}}_{i}(t)+\pi_{{v2}_{i}}(t)\cdot{\boldsymbol{G}\boldsymbol{6}\boldsymbol{G}\boldsymbol{7}}_{i}(t)$$

with transmission risks as follows:

$$\lambda_{{\boldsymbol{G}\boldsymbol{1}}_{i}}\left( t \right)=\alpha_{{\boldsymbol{G}\boldsymbol{1}}_{i}}\cdot\sum_{j=1}^{m} \beta_{ij}\cdot\left( {\boldsymbol{G}\boldsymbol{1}}_{j}\left( t \right)+{\boldsymbol{G}\boldsymbol{1}\boldsymbol{G}\boldsymbol{2}}_{j}\left( t \right)+{\boldsymbol{G}\boldsymbol{1}\boldsymbol{G}\boldsymbol{3}}_{j}\left( t \right)+{\boldsymbol{G}\boldsymbol{1}\boldsymbol{G}\boldsymbol{4}}_{j}\left( t \right)+{\boldsymbol{G}\boldsymbol{1}\boldsymbol{G}\boldsymbol{5}}_{j}\left( t \right)+{\boldsymbol{G}\boldsymbol{1}\boldsymbol{G}\boldsymbol{6}}_{j}\left( t \right)+{\boldsymbol{G}\boldsymbol{1}\boldsymbol{G}\boldsymbol{7}}_{j}\left( t \right)+\sum_{k=1}^{2} \left( {\boldsymbol{G}\boldsymbol{1}\boldsymbol{vk}}_{j}\left( t \right)+{\boldsymbol{G}\boldsymbol{1}\boldsymbol{G}\boldsymbol{2}\boldsymbol{vk}}_{j}\left( t \right)+{\boldsymbol{G}\boldsymbol{1}\boldsymbol{G}\boldsymbol{3}\boldsymbol{vk}}_{j}\left( t \right)+{\boldsymbol{G}\boldsymbol{1}\boldsymbol{G}\boldsymbol{4}\boldsymbol{vk}}_{j}\left( t \right)+{\boldsymbol{G}\boldsymbol{1}\boldsymbol{G}\boldsymbol{5}\boldsymbol{vk}}_{j}\left( t \right)+{\boldsymbol{G}\boldsymbol{1}\boldsymbol{G}\boldsymbol{6}\boldsymbol{vk}}_{j}\left( t \right)+{\boldsymbol{G}\boldsymbol{1}\boldsymbol{G}\boldsymbol{7}\boldsymbol{vk}}_{j}\left( t \right) \right) \right)$$

$$\lambda_{{\boldsymbol{G}\boldsymbol{2}}_{i}}\left( t \right)=\alpha_{{\boldsymbol{G}\boldsymbol{2}}_{i}}\cdot\sum_{j=1}^{m} \beta_{ij}\cdot\left( {\boldsymbol{G}\boldsymbol{2}}_{j}\left( t \right)+{\boldsymbol{G}\boldsymbol{1}\boldsymbol{G}\boldsymbol{2}}_{j}\left( t \right)+{\boldsymbol{G}\boldsymbol{2}\boldsymbol{G}\boldsymbol{3}}_{j}\left( t \right)+{\boldsymbol{G}\boldsymbol{2}\boldsymbol{G}\boldsymbol{4}}_{j}\left( t \right)+{\boldsymbol{G}\boldsymbol{2}\boldsymbol{G}\boldsymbol{5}}_{j}\left( t \right)+{\boldsymbol{G2G}\boldsymbol{6}}_{j}\left( t \right)+{\boldsymbol{G}\boldsymbol{2}\boldsymbol{G}\boldsymbol{7}}_{j}\left( t \right)+\sum_{k=1}^{2} \left( {\boldsymbol{G}\boldsymbol{2}\boldsymbol{vk}}_{j}\left( t \right)+{\boldsymbol{G}\boldsymbol{1}\boldsymbol{G}\boldsymbol{2}\boldsymbol{vk}}_{j}\left( t \right)+{\boldsymbol{G}\boldsymbol{2}\boldsymbol{G}\boldsymbol{3}\boldsymbol{vk}}_{j}\left( t \right)+{\boldsymbol{G}\boldsymbol{2}\boldsymbol{G}\boldsymbol{4}\boldsymbol{vk}}_{j}\left( t \right)+{\boldsymbol{G}\boldsymbol{2}\boldsymbol{G}\boldsymbol{5}\boldsymbol{vk}}_{j}\left( t \right)+{\boldsymbol{G}\boldsymbol{2}\boldsymbol{G}\boldsymbol{6}\boldsymbol{vk}}_{j}\left( t \right)+{\boldsymbol{G}\boldsymbol{2}\boldsymbol{G}\boldsymbol{7}\boldsymbol{vk}}_{j}\left( t \right) \right) \right)$$

$$\lambda_{{\boldsymbol{G}\boldsymbol{3}}_{i}}\left( t \right)=\alpha_{{\boldsymbol{G}\boldsymbol{3}}_{i}}\cdot\sum_{j=1}^{m} \beta_{ij}\cdot\left( {\boldsymbol{G}\boldsymbol{3}}_{j}\left( t \right)+{\boldsymbol{G}\boldsymbol{1}\boldsymbol{G}\boldsymbol{3}}_{j}\left( t \right)+{\boldsymbol{G}\boldsymbol{2}\boldsymbol{G}\boldsymbol{3}}_{j}\left( t \right)+{\boldsymbol{G}\boldsymbol{3}\boldsymbol{G}\boldsymbol{4}}_{j}\left( t \right)+{\boldsymbol{G}\boldsymbol{3}\boldsymbol{G}\boldsymbol{5}}_{j}\left( t \right)+{\boldsymbol{G}\boldsymbol{3}\boldsymbol{G}\boldsymbol{6}}_{j}\left( t \right)+{\boldsymbol{G}\boldsymbol{3G}\boldsymbol{7}}_{j}\left( t \right)+\sum_{k=1}^{2} \left( {\boldsymbol{G}\boldsymbol{3}\boldsymbol{vk}}_{j}\left( t \right)+{\boldsymbol{G}\boldsymbol{1}\boldsymbol{G}\boldsymbol{3}\boldsymbol{vk}}_{j}\left( t \right)+{\boldsymbol{G}\boldsymbol{2}\boldsymbol{G}\boldsymbol{3}\boldsymbol{vk}}_{j}\left( t \right)+{\boldsymbol{G}\boldsymbol{3}\boldsymbol{G}\boldsymbol{4}\boldsymbol{vk}}_{j}\left( t \right)+{\boldsymbol{G}\boldsymbol{3}\boldsymbol{G}\boldsymbol{5}\boldsymbol{vk}}_{j}\left( t \right)+{\boldsymbol{G}\boldsymbol{3}\boldsymbol{G}\boldsymbol{6}\boldsymbol{vk}}_{j}\left( t \right)+{\boldsymbol{G}\boldsymbol{3}\boldsymbol{G}\boldsymbol{7}\boldsymbol{vk}}_{j}\left( t \right) \right) \right)$$

$$\lambda_{{\boldsymbol{G}\boldsymbol{4}}_{i}}\left( t \right)=\alpha_{{\boldsymbol{G}\boldsymbol{4}}_{i}}\cdot\sum_{j=1}^{m} \beta_{ij}\cdot\left( {\boldsymbol{G}\boldsymbol{4}}_{j}\left( t \right)+{\boldsymbol{G}\boldsymbol{1}\boldsymbol{G}\boldsymbol{4}}_{j}\left( t \right)+{\boldsymbol{G}\boldsymbol{2}\boldsymbol{G}\boldsymbol{4}}_{j}\left( t \right)+{\boldsymbol{G}\boldsymbol{3}\boldsymbol{G}\boldsymbol{4}}_{j}\left( t \right)+{\boldsymbol{G}\boldsymbol{4}\boldsymbol{G}\boldsymbol{5}}_{j}\left( t \right)+{\boldsymbol{G}\boldsymbol{4}\boldsymbol{G}\boldsymbol{6}}_{j}\left( t \right)+{\boldsymbol{G}\boldsymbol{4}\boldsymbol{G}\boldsymbol{7}}_{j}\left( t \right)+\sum_{k=1}^{2} \left( {\boldsymbol{G4}\boldsymbol{vk}}_{j}\left( t \right)+{\boldsymbol{G}\boldsymbol{1}\boldsymbol{G}\boldsymbol{4}\boldsymbol{vk}}_{j}\left( t \right)+{\boldsymbol{G}\boldsymbol{2}\boldsymbol{G}\boldsymbol{4}\boldsymbol{vk}}_{j}\left( t \right)+{\boldsymbol{G}\boldsymbol{3}\boldsymbol{G}\boldsymbol{4}\boldsymbol{vk}}_{j}\left( t \right)+{\boldsymbol{G}\boldsymbol{4}\boldsymbol{G}\boldsymbol{5}\boldsymbol{vk}}_{j}\left( t \right)+{\boldsymbol{G}\boldsymbol{4}\boldsymbol{G}\boldsymbol{6}\boldsymbol{vk}}_{j}\left( t \right)+{\boldsymbol{G}\boldsymbol{4}\boldsymbol{G}\boldsymbol{7}\boldsymbol{vk}}_{j}\left( t \right) \right) \right)$$

$$\lambda_{{\boldsymbol{G}\boldsymbol{5}}_{i}}\left( t \right)=\alpha_{{\boldsymbol{G}\boldsymbol{5}}_{i}}\cdot\sum_{j=1}^{m} \beta_{ij}\cdot\left( {\boldsymbol{G}\boldsymbol{5}}_{j}\left( t \right)+{\boldsymbol{G}\boldsymbol{1}\boldsymbol{G}\boldsymbol{5}}_{j}\left( t \right)+{\boldsymbol{G}\boldsymbol{2}\boldsymbol{G}\boldsymbol{5}}_{j}\left( t \right)+{\boldsymbol{G}\boldsymbol{3}\boldsymbol{G}\boldsymbol{5}}_{j}\left( t \right)+{\boldsymbol{G}\boldsymbol{4}\boldsymbol{G}\boldsymbol{5}}_{j}\left( t \right)+{\boldsymbol{G}\boldsymbol{5}\boldsymbol{G}\boldsymbol{6}}_{j}\left( t \right)+{\boldsymbol{G}\boldsymbol{5}\boldsymbol{G}\boldsymbol{7}}_{j}\left( t \right)+\sum_{k=1}^{2} \left( {\boldsymbol{G}\boldsymbol{5}\boldsymbol{vk}}_{j}\left( t \right)+{\boldsymbol{G}\boldsymbol{1G5}\boldsymbol{vk}}_{j}\left( t \right)+{\boldsymbol{G}\boldsymbol{2}\boldsymbol{G}\boldsymbol{5}\boldsymbol{vk}}_{j}\left( t \right)+{\boldsymbol{G}\boldsymbol{3}\boldsymbol{G}\boldsymbol{5}\boldsymbol{vk}}_{j}\left( t \right)+{\boldsymbol{G}\boldsymbol{4}\boldsymbol{G}\boldsymbol{5}\boldsymbol{vk}}_{j}\left( t \right)+{\boldsymbol{G}\boldsymbol{5}\boldsymbol{G}\boldsymbol{6}\boldsymbol{vk}}_{j}\left( t \right)+{\boldsymbol{G}\boldsymbol{5}\boldsymbol{G}\boldsymbol{7}\boldsymbol{vk}}_{j}\left( t \right) \right) \right)$$

$$\lambda_{{\boldsymbol{G}\boldsymbol{6}}_{i}}\left( t \right)=\alpha_{{\boldsymbol{G}\boldsymbol{6}}_{i}}\cdot\sum_{j=1}^{m} \beta_{ij}\cdot\left( {\boldsymbol{G}\boldsymbol{6}}_{j}\left( t \right)+{\boldsymbol{G}\boldsymbol{1}\boldsymbol{G}\boldsymbol{6}}_{j}\left( t \right)+{\boldsymbol{G}\boldsymbol{2}\boldsymbol{G}\boldsymbol{6}}_{j}\left( t \right)+{\boldsymbol{G}\boldsymbol{3}\boldsymbol{G}\boldsymbol{6}}_{j}\left( t \right)+{\boldsymbol{G}\boldsymbol{4}\boldsymbol{G}\boldsymbol{6}}_{j}\left( t \right)+{\boldsymbol{G}\boldsymbol{5}\boldsymbol{G}\boldsymbol{6}}_{j}\left( t \right)+{\boldsymbol{G}\boldsymbol{6}\boldsymbol{G}\boldsymbol{7}}_{j}\left( t \right)+\sum_{k=1}^{2} \left( {\boldsymbol{G}\boldsymbol{6}\boldsymbol{vk}}_{j}\left( t \right)+{\boldsymbol{G}\boldsymbol{1}\boldsymbol{G}\boldsymbol{6}\boldsymbol{vk}}_{j}\left( t \right)+{\boldsymbol{G2}\boldsymbol{G}\boldsymbol{6}\boldsymbol{vk}}_{j}\left( t \right)+{\boldsymbol{G}\boldsymbol{3}\boldsymbol{G}\boldsymbol{6}\boldsymbol{vk}}_{j}\left( t \right)+{\boldsymbol{G}\boldsymbol{4}\boldsymbol{G}\boldsymbol{6}\boldsymbol{vk}}_{j}\left( t \right)+{\boldsymbol{G}\boldsymbol{5}\boldsymbol{G}\boldsymbol{6}\boldsymbol{vk}}_{j}\left( t \right)+{\boldsymbol{G}\boldsymbol{6}\boldsymbol{G}\boldsymbol{7}\boldsymbol{vk}}_{j}\left( t \right) \right) \right)$$

$$\lambda_{{\boldsymbol{G}\boldsymbol{7}}_{i}}\left( t \right)=\alpha_{{\boldsymbol{G}\boldsymbol{7}}_{i}}\cdot\sum_{j=1}^{m} \beta_{ij}\cdot\left( {\boldsymbol{G}\boldsymbol{7}}_{j}\left( t \right)+{\boldsymbol{G}\boldsymbol{1}\boldsymbol{G}\boldsymbol{7}}_{j}\left( t \right)+{\boldsymbol{G}\boldsymbol{2}\boldsymbol{G}\boldsymbol{7}}_{j}\left( t \right)+{\boldsymbol{G}\boldsymbol{3}\boldsymbol{G}\boldsymbol{7}}_{j}\left( t \right)+{\boldsymbol{G}\boldsymbol{4}\boldsymbol{G}\boldsymbol{7}}_{j}\left( t \right)+{\boldsymbol{G}\boldsymbol{5}\boldsymbol{G}\boldsymbol{7}}_{j}\left( t \right)+{\boldsymbol{G}\boldsymbol{6}\boldsymbol{G}\boldsymbol{7}}_{j}\left( t \right)+\sum_{k=1}^{2} \left( {\boldsymbol{G}\boldsymbol{7}\boldsymbol{vk}}_{j}\left( t \right)+{\boldsymbol{G}\boldsymbol{1}\boldsymbol{G}\boldsymbol{7}\boldsymbol{vk}}_{j}\left( t \right)+{\boldsymbol{G}\boldsymbol{2}\boldsymbol{G}\boldsymbol{7}\boldsymbol{vk}}_{j}\left( t \right)+{\boldsymbol{G3}\boldsymbol{G}\boldsymbol{7}\boldsymbol{vk}}_{j}\left( t \right)+{\boldsymbol{G}\boldsymbol{4}\boldsymbol{G}\boldsymbol{7}\boldsymbol{vk}}_{j}\left( t \right)+{\boldsymbol{G}\boldsymbol{5}\boldsymbol{G}\boldsymbol{7}\boldsymbol{vk}}_{j}\left( t \right)+{\boldsymbol{G}\boldsymbol{6}\boldsymbol{G}\boldsymbol{7}\boldsymbol{vk}}_{j}\left( t \right) \right) \right)$$

and the following condition ($n=7$ serotype groups):

$$\boldsymbol{S}_{i}\left( t \right)+\sum_{k=1}^{n} \left( \boldsymbol{Gk}_{i}\left( t \right)+{\boldsymbol{Gkv}\boldsymbol{1}}_{i}\left( t \right)+{\boldsymbol{Gkv}\boldsymbol{2}}_{i}\left( t \right) \right)+\sum_{k=1}^{n-1} \sum_{l=k+1}^{n} \left( \boldsymbol{GkGl}_{i}\left( t \right)+{\boldsymbol{GkGlv}\boldsymbol{1}}_{i}\left( t \right)+{\boldsymbol{GkGlv}\boldsymbol{2}}_{i}\left( t \right) \right)=1 \text{∀}i=1,\ldots,m$$

**4. Calculation of carriage prevalence**

Due to a lack of suitable pneumococcal carriage studies regarding the population in Germany, we estimated carriage for each age and ST group using German IPD incidence rates (IRs). As an example, the procedure is shown for the epidemiological year 2005/06, which was assumed representative for the pre-PCV vaccination steady-state of ST distributions:

For German IPD IRs we used two data sources: (i) IPD IRs taken from the literature (hereafter termed “adjusted IRs”), which have been estimated using the capture-recapture method [10–12], and (ii) raw data of potentially underreported IPD case counts (termed “reported IRs”) obtained courtesy of Mark van der Linden from the German National Reference Center for Streptococci (Aachen, Germany). For a given epidemiological year we applied the following algorithm:

From the raw data we calculated reported IPD IRs for each year separately for children (0-15y) and adults (≥16y). For these calculations we used age distributions reported by the German Federal Office of Statistics (see section on the demographic submodel). Comparing these reported IRs to the adjusted IPD IRs of children and adults, we calculated year-specific underreporting factors (UFs) separately for children and adults as well as corresponding 95% confidence intervals (CIs). This calculation was based on the assumption that UFs differ between children and adults but do not depend on ST.

UFs for children (and their CIs) can be estimated using the described method until 2015 [12]. For subsequent years, no IPD IRs have been reported for children in Germany. Hence, from 2016 we assumed the UF to have reached a steady-state of 1.5 (average of the years 2010-2015). The respective CI was assumed to be [1.0; 2.0], which approximately corresponds to the average CI width of the years 2010-2015. The situation is more complicated for adults. Adjusted IPD IRs for adults in Germany have only been reported for the pre-PCV era [10]. In order to estimate UFs after PCV introduction, we thus used UK data [13] as a reference point for our IR calculations. As IRs are not directly comparable between both countries, we only used relative IR changes. The last reported pre-PCV IR (year 2005) served as the respective baseline. We assumed a constant linear reduction of adult IPD IRs (0.3 per 100,000 per year) until 2013. Thereafter, a 40% increase within three years (until 2016) was assumed. These relative changes were applied to the German baseline to estimate adjusted IRs in Germany until 2016, which were then compared to the reported IRs. Based on these assumptions, an adult UF of 1.5 was estimated for 2016. As with children, we assumed this value constant for subsequent years, also motivated by the fact that for technical reasons (e.g., reorganized reporting system) UFs are no longer expected to differ between children and adults (see S2 Fig).

From the raw data we calculated reported IPD IRs for each ST and age group (56 values total per year; see S3 Fig). If zero cases had been reported for any of the subgroups for a given year, we assumed 0.5 cases for the respective groups to avoid numerical artifacts. These reported IRs were multiplied by the UFs estimated in the previous step, separately for children and adult age groups, to obtain adjusted IRs (and respective 95% CIs) for each subgroup (see S4 Fig).

As the main readout of the model are pneumococcal carriage prevalences, IPD IRs needed to be converted into carriage prevalences using IPD case-carrier ratios (CCRs). For this purpose, we used the age and ST group-specific adjusted IPD IRs calculated in the previous step and applied IPD CCRs obtained from the literature [14]. We assumed CCRs constant over time.

Choi et al. [14] performed CCR estimation for 3 different ST groups: PCV7 (STs 4, 6B, 9V, 14, 18C, 19F, 23F; hereafter “VT1”), PCV13 non-PCV7 -ST 1 (STs 3, 5, 6A, 7F, 19A; “VT2”), and all other STs (“NVT”). CCRs are visualized in S5 Fig. In order to apply this data to our seven ST groups, the following assumptions were made:

1. PCV7: use VT1 estimates
2. PCV13 non-PCV7 -ST 3 +ST 6C: use VT2 estimates
3. ST 3 only: use VT2 estimates (ST 3 is included in VT2 definition)
4. PCV15 non-PCV13: use NVT estimates (STs 22F and 33F are included in NVT definition)
5. PCV20 non-PCV15: use NVT estimates (STs 8, 10A, 11A, 12F, and 15B are included in NVT definition)
6. PPSV23 non-PCV20: use NVT estimates (STs 2, 9N, 17F, and 20 are included in NVT definition)
7. Other STs: use NVT estimates

Due to a lack of suitable data we were unable to estimate CCRs that are more closely associated with the ST group definitions in our model. Particularly, we applied the same estimates for all non-vaccine ST groups. We calculated carriage prevalences for each of the 56 age and ST subgroups as follows: IR divided by CCR. To obtain percentages, the resulting values were divided by 100,000. Carriage prevalences for the epidemiological year 2005/06 (pre-PCV era) are found in S6 Fig.


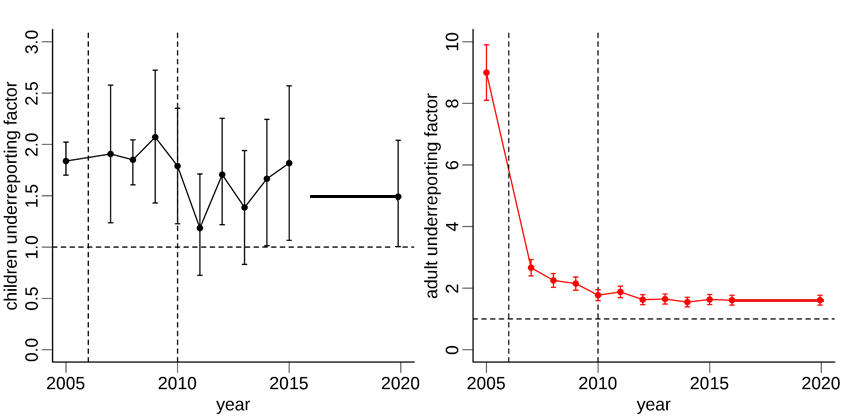


**S2 Fig. Estimated underreporting factors (UFs) for children (black) and adults (red), from the pre-PCV era (2005) to the latest available data (2020).** Shown are UF point estimates (for a detailed description see text) and 95% CIs. Note the assumption of constant UFs starting from 2016. Dashed vertical lines represent the start of PCV7 (2006) and PCV13 (2010) vaccinations, respectively.

**
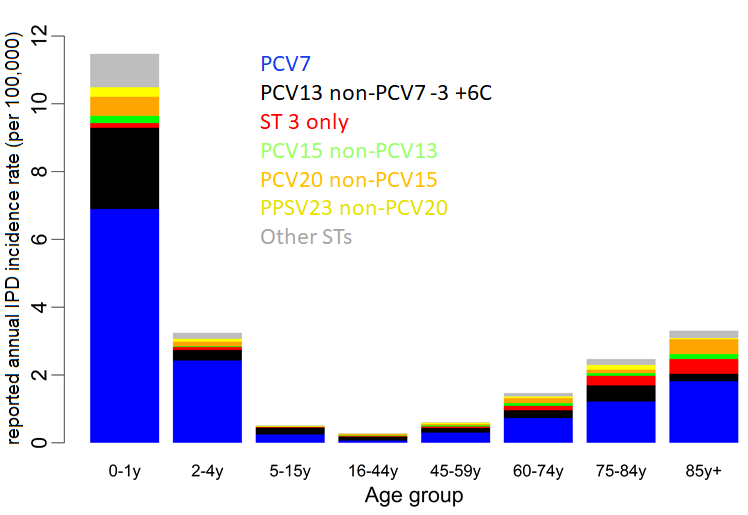
S3 Fig. Stacked bar-plot of reported IPD incidence rates (IRs) for the epidemiological year 2005 (pre-PCV vaccination era) in Germany, separately for the eight age and seven ST groups used in the model.** These IRs were calculated directly from the reported IPD case counts (German Reference Center for Streptococci) without accounting for potential underreporting/underdiagnosing and must not be confused with adjusted IPD IRs. For the latter see S4 Fig.


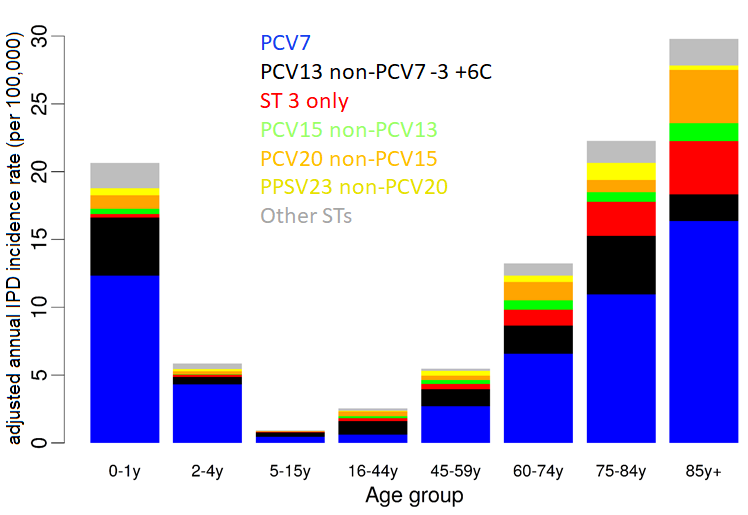
**S4 Fig. Stacked bar-plot of adjusted IPD incidence rates (IRs) for the epidemiological year 2005 (pre-PCV vaccination era) in Germany, separately for the eight age and seven ST groups used in the model.** These IRs were calculated from the reported IRs shown in S3 Fig applying the underreporting factors (UFs) estimated for 2005 (S2 Fig), namely UF = 1.8 (children age groups <2y, 2-4y, 5-15y) and UF = 9.0 (adult age groups 16-44y, 45-59y, 60-74y, 75-84y, ≥85y).


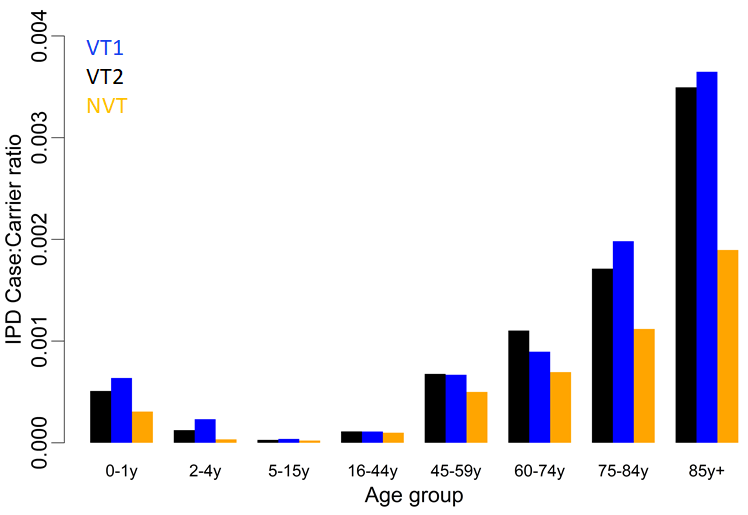
**S5 Fig. Bar-plot of IPD case-carrier ratios (CCRs) for three different ST groups.** VT1 (STs 4, 6B, 9V, 14, 18C, 19F, 23F; blue), VT2 (STs 3, 5, 6A, 7F, 19A; black), and NVT (all other STs; orange). The figure was adapted from Choi et al. [14] and aggregated into eight age groups as defined in our model. We applied this data to our seven ST groups (for a detailed description see text).


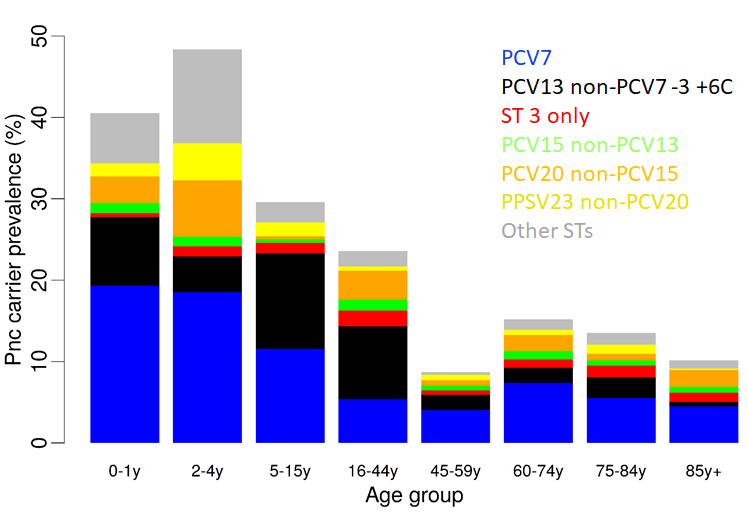
**S6 Fig. Stacked bar-plot of pneumococcal carriage prevalences (given in percent) for epidemiological year 2005 (pre-PCV vaccination era).** The data is shown separately for the eight age and seven ST groups defined in our model. Remaining percentages to 100% in the respective age groups represent non-carriers. Prevalences were calculated using adjusted IPD IRs from the same year (estimated for all subgroups) and IPD CCRs from the literature (see S5 Fig).


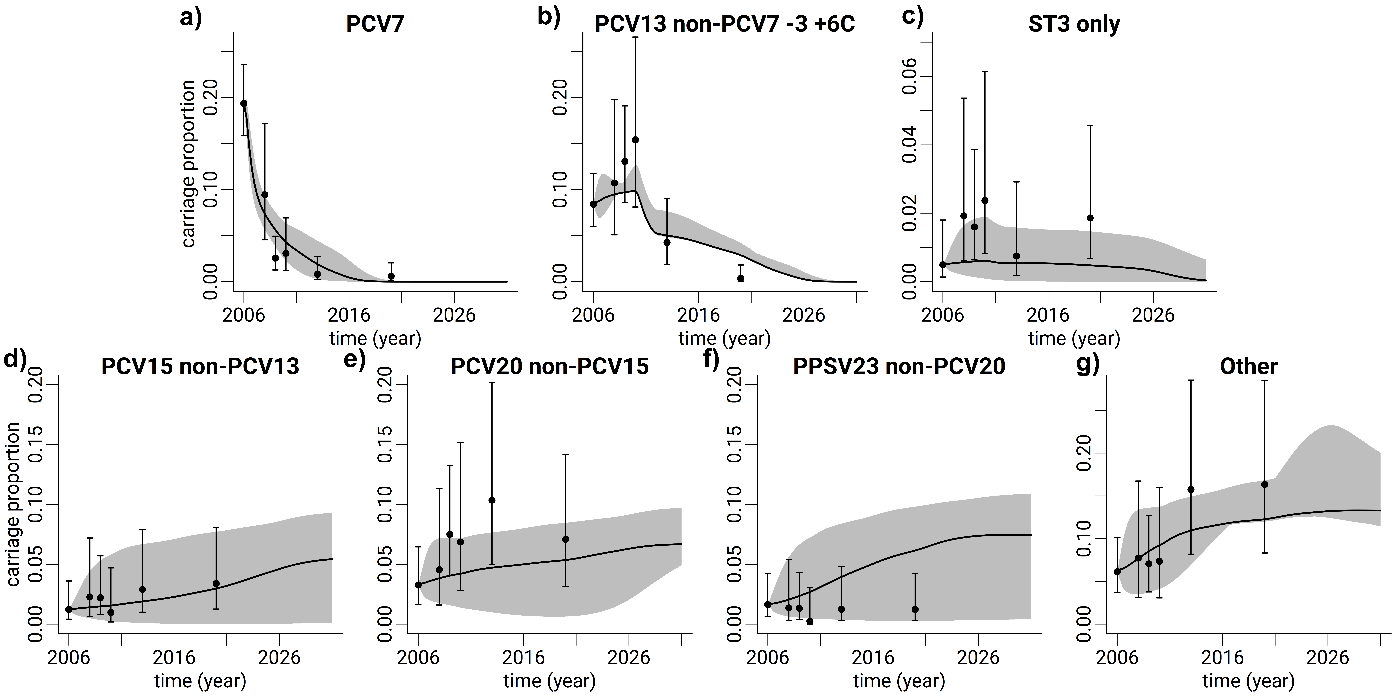


**S7 Fig. Impact of competition parameters on predictions of vaccination model for children <2y.** Shown are vaccine serotype groups (upper row) PCV7 (a), PCV13 non-PCV7 -3 +6C (b), serotype 3 (c), and non-vaccine serotype groups (lower row) PCV15 non-PCV13 (d), PCV20 non-PCV15 (e), PPSV23 non-PCV20 (f), and Other (g). Data points (pneumococcal carriage proportions regarding all individuals of the age group in Germany, including non-carriers) are shown as filled circles with 95% CIs. Data points were calculated from adjusted IPD incidence rates via CCRs. Simulation results are depicted by lines with shades representing the variation of competition parameters.


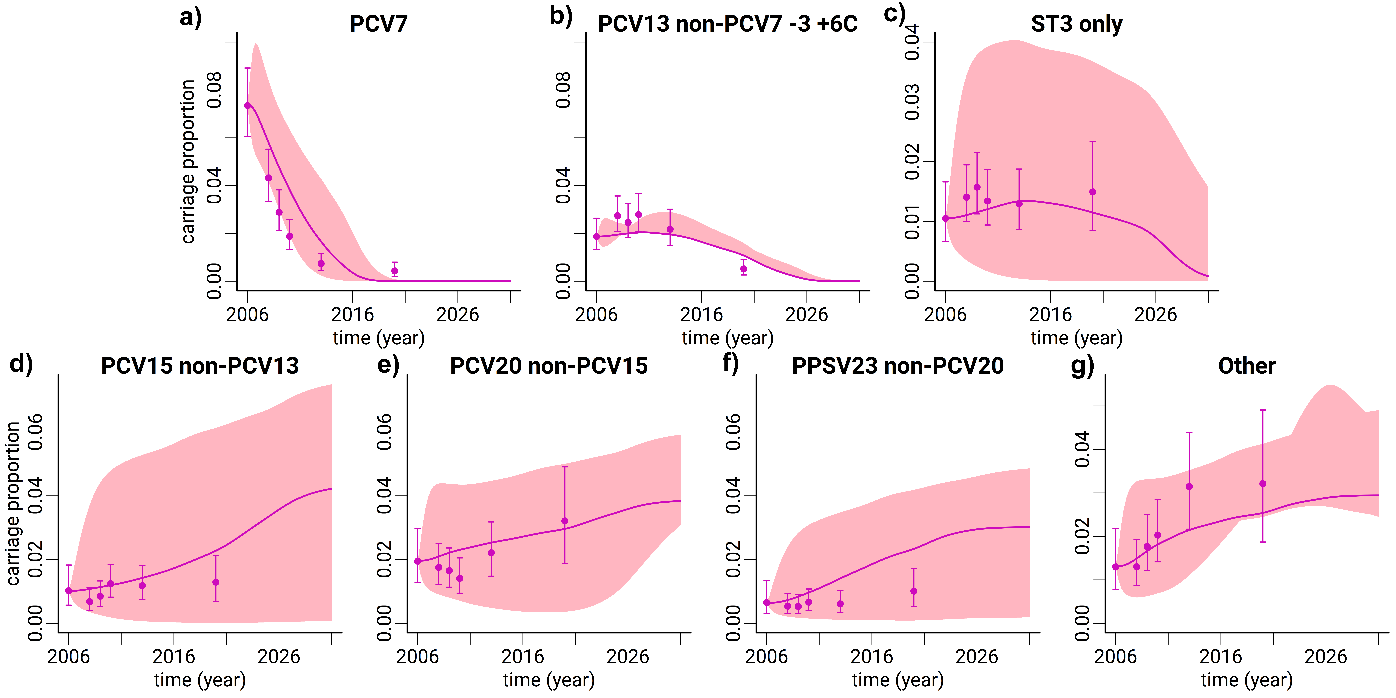


**S8 Fig. Impact of competition parameters on predictions of vaccination model for adults 60-74y.** Shown are vaccine serotype groups (upper row) PCV7 (a), PCV13 non-PCV7 -3 +6C (b), serotype 3 (c), and non-vaccine serotype groups (lower row) PCV15 non-PCV13 (d), PCV20 non-PCV15 (e), PPSV23 non-PCV20 (f), and Other (g). Data points (pneumococcal carriage proportions regarding to all individuals of the age group in Germany, including non-carriers) are shown as filled circles with 95% CIs. Data points were calculated from adjusted IPD incidence rates via CCRs. Simulation results are depicted by lines with shades representing the variation of competition parameters.


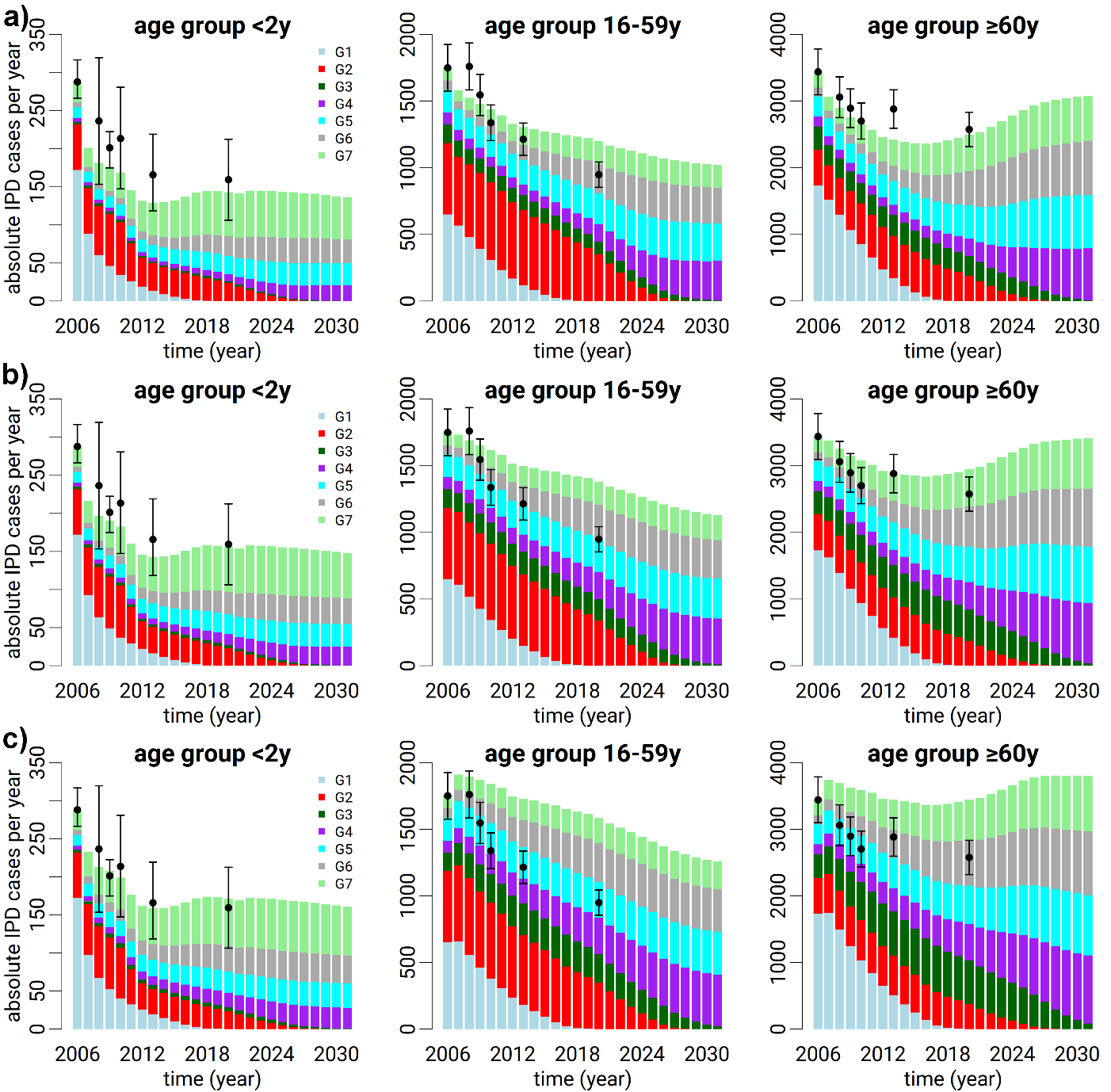


**S9 Fig. Stacked bar plots of predicted absolute IPD cases per year from 2006 to 2031 in Germany for three different age groups (<2y, 16-59y, ≥60y) and three different configurations of competition parameters (**$\boldsymbol{c=0.4}$ **[upper row],** $\boldsymbol{c=0.5}$ **[middle row],** $\boldsymbol{c=0.6}$ **[lower row]).** The stacked bars illustrate the serotype groups defined in Table 1 of the main manuscript: PCV7 (*G1*; light blue), PCV13 non-PCV7 -3 +6C (*G2*; red), serotype 3 only (*G3*; dark green), PCV15 non-PCV13 (*G4*; purple), PCV20 non-PCV15 (*G5*; cyan), PPSV23 non-PCV20 (*G6*; gray), Other serotypes (*G7*; light green). Heights of the individual bars correspond to the model prediction. Data points (reported IPD case numbers in Germany multiplied by an estimated year-specific underreporting factor) are shown as filled circles with 95% CIs. The middle row (b) is identical to Fig 6 of the main manuscript and is given here again to facilitate comparison to the other scenarios of serotype competition.

References

**1**. German Federal Office of Statistics. 14th coordinated population projection (base 2018). Available from: https://www.destatis.de/EN/Themes/Society-Environment/Population/Population-Projection/update-population-projection.html.

**2**. Högberg L, Geli P, Ringberg H, Melander E, Lipsitch M, Ekdahl K. Age- and serogroup-related differences in observed durations of nasopharyngeal carriage of penicillin-resistant pneumococci. Journal of Clinical Microbiology. 2007; 45:948–52. Epub 2007/01/03. doi: 10.1128/JCM.01913-06 PMID: 17202280.

**3**. Mossong J, Hens N, Jit M, Beutels P, Auranen K, Mikolajczyk R, et al. Social contacts and mixing patterns relevant to the spread of infectious diseases. PLoS Med. 2008; 5:e74. doi: 10.1371/journal.pmed.0050074 PMID: 18366252.

**4**. Flasche S, van Hoek AJ, Sheasby E, Waight P, Andrews N, Sheppard C, et al. Effect of pneumococcal conjugate vaccination on serotype-specific carriage and invasive disease in England: a cross-sectional study. PLoS Med. 2011; 8:e1001017. Epub 2011/04/05. doi: 10.1371/journal.pmed.1001017 PMID: 21483718.

**5**. van Hoek AJ, Sheppard CL, Andrews NJ, Waight PA, Slack MPE, Harrison TG, et al. Pneumococcal carriage in children and adults two years after introduction of the thirteen valent pneumococcal conjugate vaccine in England. Vaccine. 2014; 32:4349–55. Epub 2014/03/21. doi: 10.1016/j.vaccine.2014.03.017 PMID: 24657717.

**6**. Sá-Leão R, Nunes S, Brito-Avô A, Frazão N, Simões AS, Crisóstomo MI, et al. Changes in pneumococcal serotypes and antibiotypes carried by vaccinated and unvaccinated day-care centre attendees in Portugal, a country with widespread use of the seven-valent pneumococcal conjugate vaccine. Clin Microbiol Infect. 2009; 15:1002–7. Epub 2009/04/23. doi: 10.1111/j.1469-0691.2009.02775.x PMID: 19392883.

**7**. Davis SM, Deloria-Knoll M, Kassa HT, O'Brien KL. Impact of pneumococcal conjugate vaccines on nasopharyngeal carriage and invasive disease among unvaccinated people: review of evidence on indirect effects. Vaccine. 2013; 32:133–45. Epub 2013/05/16. doi: 10.1016/j.vaccine.2013.05.005 PMID: 23684824.

**8**. Dunais B, Bruno P, Carsenti-Dellamonica H, Touboul P, Dellamonica P, Pradier C. Trends in nasopharyngeal carriage of Streptococcus pneumoniae among children attending daycare centers in southeastern France from 1999 to 2006. Pediatr Infect Dis J. 2008; 27:1033–5. doi: 10.1097/INF.0b013e31817bb8cf PMID: 18955896.

**9**. Félix S, Handem S, Nunes S, Paulo AC, Candeias C, Valente C, et al. Impact of private use of the 13-valent pneumococcal conjugate vaccine (PCV13) on pneumococcal carriage among Portuguese children living in urban and rural regions. Vaccine. 2021; 39:4524–33. Epub 2021/06/25. doi: 10.1016/j.vaccine.2021.06.035 PMID: 34183206.

**10**. Reinert RR, Haupts S, van der Linden M, Heeg C, Cil MY, Al-Lahham A, et al. Invasive pneumococcal disease in adults in North-Rhine Westphalia, Germany, 2001-2003. Clin Microbiol Infect. 2005; 11:985–91. doi: 10.1111/j.1469-0691.2005.01282.x PMID: 16307552.

**11**. Rückinger S, van der Linden M, Reinert RR, Kries R von, Burckhardt F, Siedler A. Reduction in the incidence of invasive pneumococcal disease after general vaccination with 7-valent pneumococcal conjugate vaccine in Germany. Vaccine. 2009; 27:4136–41. Epub 2009/05/09. doi: 10.1016/j.vaccine.2009.04.057 PMID: 19406190.

**12**. Weinberger R, Kries R von, van der Linden M, Rieck T, Siedler A, Falkenhorst G. Invasive pneumococcal disease in children under 16 years of age: Incomplete rebound in incidence after the maximum effect of PCV13 in 2012/13 in Germany. Vaccine. 2018; 36:572–7. Epub 2017/12/16. doi: 10.1016/j.vaccine.2017.11.085 PMID: 29258705.

**13**. Ladhani SN, Collins S, Djennad A, Sheppard CL, Borrow R, Fry NK, et al. Rapid increase in non-vaccine serotypes causing invasive pneumococcal disease in England and Wales, 2000–17: a prospective national observational cohort study. The Lancet Infectious Diseases. 2018; 18:441–51. doi: 10.1016/S1473-3099(18)30052-5.

**14**. Choi YH, Jit M, Flasche S, Gay N, Miller E. Mathematical modelling long-term effects of replacing Prevnar7 with Prevnar13 on invasive pneumococcal diseases in England and Wales. PLoS One. 2012; 7:e39927. Epub 2012/07/13. doi: 10.1371/journal.pone.0039927 PMID: 22808073.
